# Genome-wide identification, phylogeny and expression analysis of GRAS gene family in tomato

**DOI:** 10.1186/s12870-015-0590-6

**Published:** 2015-08-25

**Authors:** Wei Huang, Zhiqiang Xian, Xia Kang, Ning Tang, Zhengguo Li

**Affiliations:** Genetic Engineering Research Center, School of Life Sciences, Chongqing University, Chongqing, 400044 People’s Republic China

## Abstract

**Background:**

GRAS transcription factors usually act as integrators of multiple growth regulatory and environmental signals, including axillary shoot meristem formation, root radial pattering, phytohormones, light signaling, and abiotic/biotic stress. However, little is known about this gene family in tomato (*Solanum lycopersicum*), the most important model plant for crop species with fleshy fruits.

**Results:**

In this study, 53 GRAS genes were identified and renamed based on tomato whole-genome sequence and their respective chromosome distribution except 19 members were kept as their already existed name. Multiple sequence alignment showed typical GRAS domain in these proteins. Phylogenetic analysis of GRAS proteins from tomato, *Arabidopsis*, *Populus*, *P.mume*, and Rice revealed that SlGRAS proteins could be divided into at least 13 subfamilies. *SlGRAS24* and *SlGRAS40* were identified as target genes of miR171 using5’-RACE (Rapid amplification of cDNA ends). qRT-PCR analysis revealed tissue-/organ- and development stage-specific expression patterns of *SlGRAS* genes. Moreover, their expression patterns in response to different hormone and abiotic stress treatments were also investigated.

**Conclusions:**

This study provides the first comprehensive analysis of GRAS gene family in the tomato genome. The data will undoubtedly be useful for better understanding the potential functions of GRAS genes, and their possible roles in mediating hormone cross-talk and abiotic stress in tomato as well as in some other relative species.

**Electronic supplementary material:**

The online version of this article (doi:10.1186/s12870-015-0590-6) contains supplementary material, which is available to authorized users.

## Background

Transcription factors (TFs) are important part of the functional genomics. Since the first transcription factor was found in maize [1], a large number of TFs have been proven to participate in various physiological processes and regulatory networks in higher plants. GRAS proteins are named after GAI, RGA and SCR [2–4], the first three functionally identified members in this family. Typically, proteins of this family exhibit considerable sequence homology to each other in their C-terminus, within which motifs including LHR I, VHIID, LHR II, PFYRE and SAW can be recognized in turn [5–7]. In contrast, N-terminus of GRAS family varies in length and sequence, which seems like the major contributor to the functional specificity of each gene [6, 8].

By far, GRAS gene family has been genome-wide explored in several plant species, including *Populus*, *Arabidopsis*, rice, Chinese cabbage, *Prunus mume*, and pine [9–12]. However, only small number of GRAS proteins were functionally characterized, including some members identified in *Zea mays*, *Petunia hybrida*, *Medicago truncatula*, *Lilium longiflorum* [13–16]. These genes play crucial roles in diverse fundamental processes of plant growth and development. For instance, the most widely known sub-branch of GRAS proteins, which share the amino acid sequence DELLA in their N-terminal region and thus are referred as DELLA proteins, function as repressors of gibberellin signaling [4]. The SCR and SHR, which belong to two different sub-branches of GRAS family, are both involved in radial organization of the root through forming a SCR/SHR complex [17]. Two independent studies demonstrated that endodermis-expressed SCL3 acted as an integrator downstream of the GA/DELLA and SCR/SHR pathways, mediating the GA-promoted cell elongation during root development [18, 19]. Another sub-branch, which contains 4 highly homologous in *Arabidopsis*, PAT1, SCL5, SCL13, and SCL21, are involved in light signaling pathways. Interestingly, PAT1, SCL5, SCL21 are positive regulators of phytochrome-A signal transduction while SCL13 is mainly participated in phytochrome-B signal transduction [20–22]. Two GRAS proteins, NSP1 and NSP2 can form a DNA binding complex which is essential for nodulation signaling in legumes [23]. MOC1, mainly expressed in the axillary buds, has a pivotal role in controlling rice tillering [24]. *Ls* and *LAS*, the homologous gene of MOC1 in tomato and *Arabidopsis*, also act in the axillary meristem initiation of tomato [25, 26]. In addition, LiSCL is a transcriptional activator of some meiosis-associated genes, participates in the microsporogenesis of the lily anther [16]. HAM mediates signals from differentiating cells for controlling shoot meristem maintenance in the *Petunia* [14]. And three *Arabidopsis* orthologs of *Petunia* HAM, SCL6/SCL6-IV, SCL22/SCL6-III and SCL27/SCL6-II, also known as targets of post-transcriptional degradation by miRNA170/171, have been demonstrated to play an important role in the proliferation of meristematic cells, polar organization and chlorophyll synthesis [27–29].

Tomato (*Solanum lycopersicum*) is an important crop because of its great nutritive and commercial value, and also a good model plant for fleshy fruit development. With the release of the whole genome sequence of tomato [30], it is very convenient to comprehensive analysis an entire gene family now. To date, transcription factor families like ERF, WRKY, SBP-box, IAA, ARF, and TCP have already been identified in tomato [31–36]. Here, considering the important role of GRAS proteins in plant growth regulation and the lack of information about this gene family in the crop, we describe on the first characterization of the entire GRAS gene family of transcription factors in tomato. The present work identified 53 putative *SlGRAS* genes, together with analyzing their gene classification, chromosome distribution, phylogenetic comparison and exon-intron organization. In addition, the expression profile analysis of *SlGRAS* genes by real time qPCR in different stages of vegetative and reproductive development were performed, and their transcript abundance in response to different hormones and abiotic stress treatments were also investigated. This study provides details of GRAS gene family and facilitates the further functional characterization of *GRAS* genes in tomato.

## Results

### Identification and multiple sequence analysis of *SlGRAS* genes

**Fig. 1:**
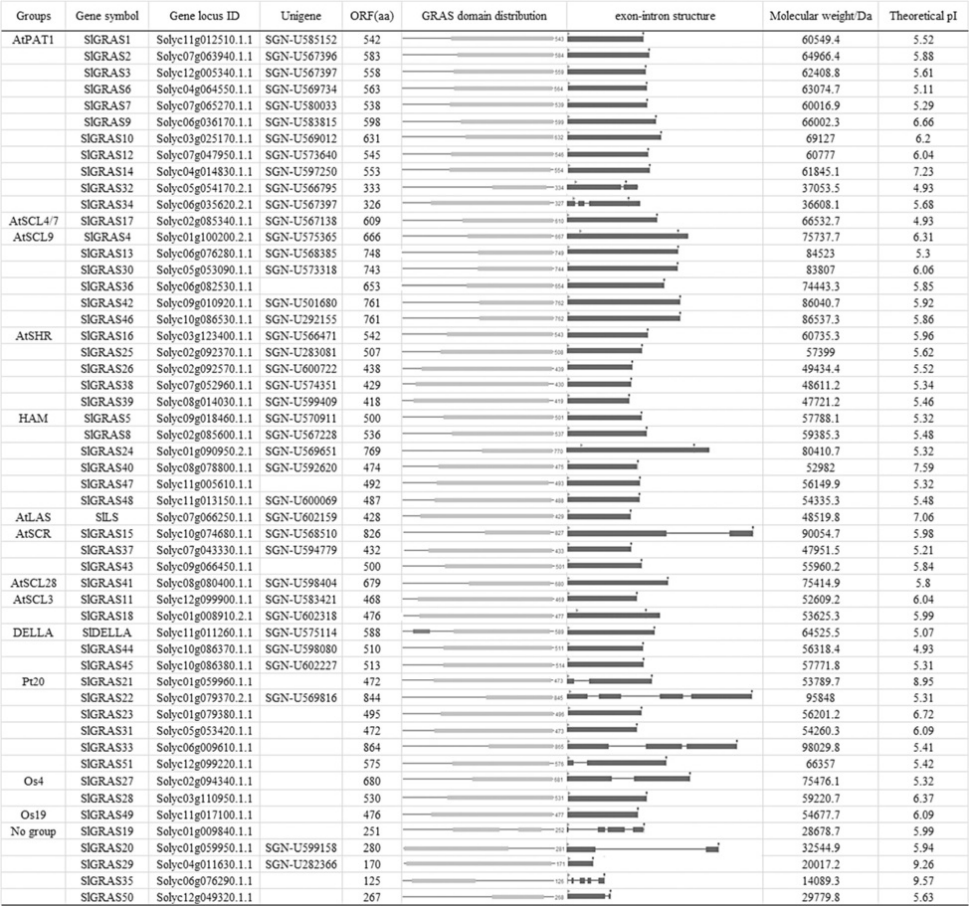
The information of 53 GRAS transcription factors identified in tomato genome. SlGRAS19, SlGRAS20, SlGRAS29, SlGRAS35, SlGRAS50, whose full amino acid length less than 300 were distributed to “No group” and were excluded from some of the following analyses

**Fig. 2:**
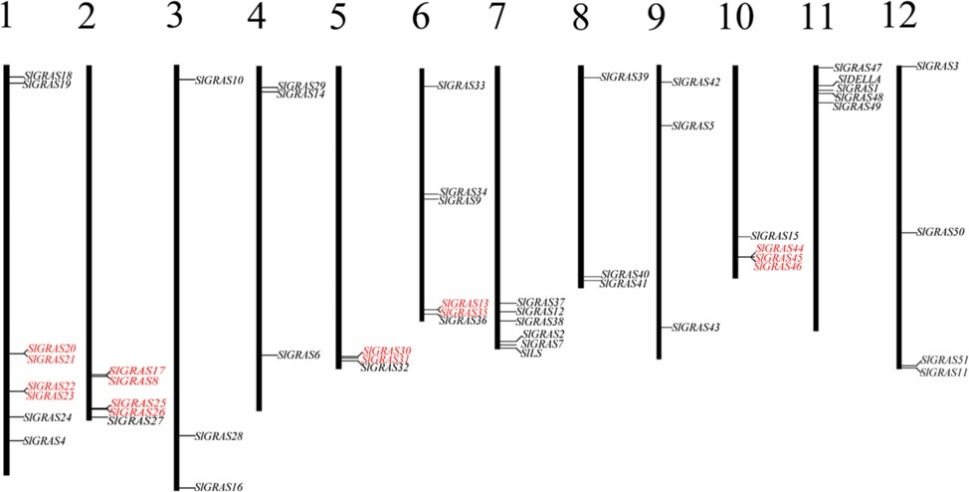
Positions of GRAS gene family members on the *Solanum lycopersicum* chromosomes. Tandemly duplicated genes were indicated in *red* colour

Phytozome Search Tools (http://www.phytozome.net/search.php) was performed using keywords search with “GRAS”, and 54 genes were found when searched against the pfam GRAS hidden-Markov model (PF03154). However, one of them, Solyc09g090830.2.1 was excluded because it represented only part of the GRAS domain and was annotated as an BolA-like protein in the Tomato Genome database (ITAG2.4 Release: genomics annotations). Meanwhile, BLASTP analysis using the amino acid (AA) sequences of characterized AtGRAS proteins as queries obtained 51 previously annotated GRAS members in tomato WGS Chromosomes (SL2.50), which were all included in the 53 *GRAS* genes identified above. Subsequently, online bioinformatics tools, ExPASy-PROSITE (http://prosite.expasy.org/) and TBLASTN of NCBI showed that all sequences contained a GRAS domain, thus further confirmed the authenticity of the identified *SlGRAS* genes. Taken together, a total of 53 distinct GRAS transcription factors were indentified in tomato genome (Fig. 1 and Additional file 1). All of the 53 tomato *GRAS* genes were mapped onto the 12 tomato chromosomes and then renamed based on their distributions and relative linear orders among the respective chromosome (Fig. 2), among which, *SlDELLA* and *SlLs* were kept as their already existed name, and so did the *SlGRAS1* to *SlGRAS17*, which were previously described by Mayrose et al. [37]. The tomato *GRAS* genes display uneven distributions across the chromosomes., Chr1 occupies the largest number of *GRAS* genes (n = 8), 4, 4, 5, 5, 6, and 6 *GRAS* genes were found on Chr10, Chr 12, Chr2, Chr11, Chr 6, and Chr7, respectively, and the other 5 chromosomes each have 3 *GRAS* genes. Besides, there are 15 *SlGRAS* genes (*SlGRAS20*, *SlGRAS21*, *SlGRAS22*, *SlGRAS23*, *SlGRAS17*, *SlGRAS8*, *SlGRAS25*, *SlGRAS26*, *SlGRAS30*, *SlGRAS31*, *SlGRAS13*, *SlGRAS35*, *SlGRAS44*, *SlGRAS45*, *SlGRAS46*) clustered into seven tandem duplication event regions on tomato chromosome 1 (2 clusters), 2 (2 clusters), 5 (1 cluster), 6 (1 cluster) and 10 (1 cluster) (Fig. 2 and Additional file 2). The size of the deduced GRAS proteins varies greatly, ranging from 125 amino acids (*SlGRAS35*) to 864 amino acids (*SlGRAS33*). The molecular weight varies from 14 to 98 kDa, and the predicted theoretical pI also varies from 4.93 to 9.57. These facts indicate that different SlGRAS proteins might function in different microenvironments. Most members possess a variable N-termianl and a single highly conserved C-terminal GRAS domain. However, three members (*SlGRAS20*, *SlGRAS29*, and *SlGRAS35*) present their GRAS domains in the N-terminal part, whereas *SlGRAS19*, contains two GRAS domains. Interestingly, 41 GRAS genes with only one exon were found, which seems like a widespread phenomenon of this gene family observed in many plant species [9–12]. The exon number of other *GRAS* genes ranged from two to five. More detailed information about each *GRAS* gene was shown in Fig. 1, including the *GRAS* gene group name, gene locus number, the length of coding sequences, the schematic plots of GRAS domain, the exon-intron structure, the molecular weight, and the theoretical pI information.

**Fig. 3:**
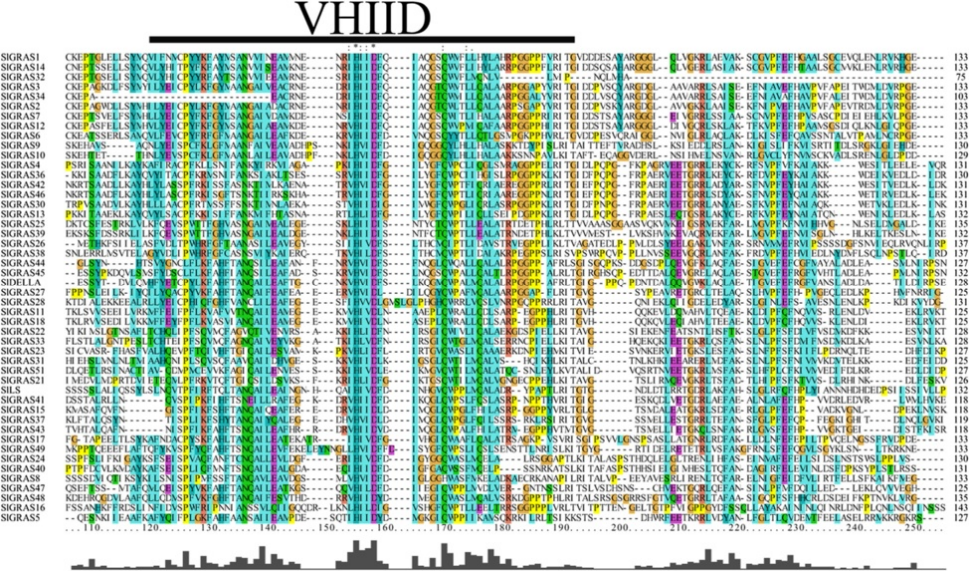
Multiple sequence alignment of the 48 GRAS domain from tomato *GRAS* genes obtained by ClustalX and manual correction. The most conserved motif of GRAS domain, VHIID, was underlined

From the alignment of predicted GRAS domain sequences we found members containing partial GRAS domains with missing motifs, some of which were severely truncated. In tomato, for instance, the GRAS domain of SlGRAS35 could be as short as 85 amino acids, while the typical GRAS domain had a minimum length of about 350 amino acids (e.g., At4g00150, SlGRAS38), thereby 5 non-canonical GRAS proteins (SlGRAS19, SlGRAS20, SlGRAS29, SlGRAS35, SlGRAS50) were excluded from some of the following analyses (multiple sequence alignment and phylogenetic analysis) because of the low reliability by incorporating these fragments. Furthermore, although the multiple sequence analysis showed a low overall identity among the 48 analyzed SlGRAS proteins, the 5 most prominent motifs, including leucine-rich region I (LR I), VHIID, leucine-rich region II (LR II), PFYRE, and SAW could be observed in their GRAS domains (Fig. 3 and Additional file 3).

### Phylogenetic analysis and classification of GRAS members from *Arabidopsis* and tomato

**Fig. 4:**
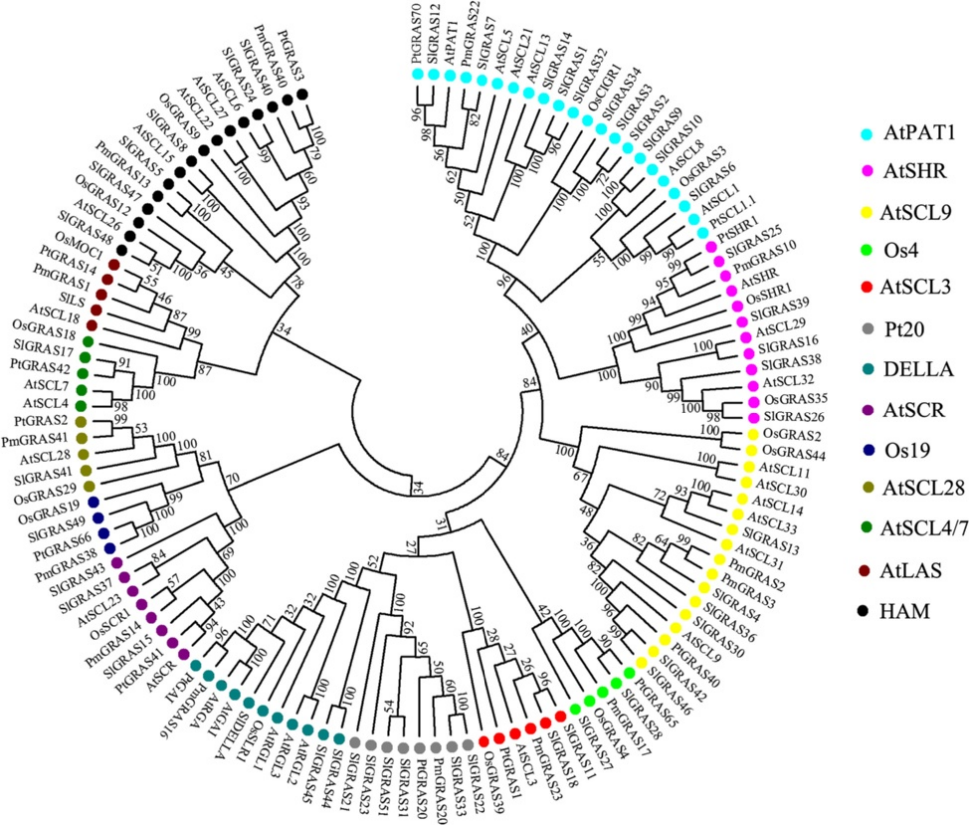
Phylogenetic analysis of GRAS proteins. The phylogenetic tree was generated by Neighbor-Joining method derived from ClustalX alignment of 48, 32, 14, 14, and 16GRAS amino acid sequences from tomato, *Arabidopsis*, *Populus*, *P.mume*, and rice, respectively. Members in the same sub-branch were marked by circle filled with same color

**Fig. 5:**
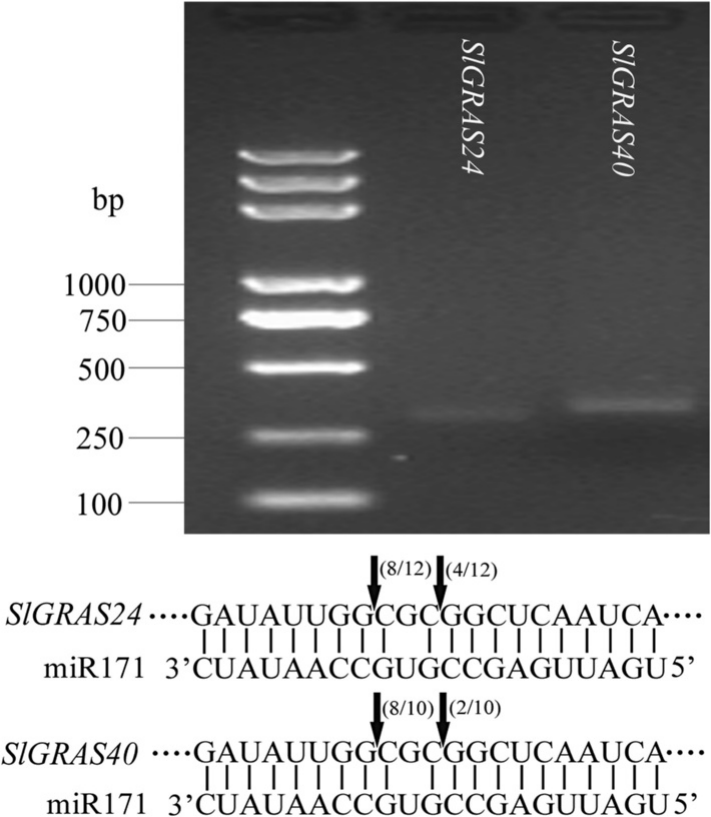
Cleavage sites of miR171 at complementary sequences of *SlGRAS24* and *SlGRAS40* determined by 5’-RACE. The electrophoretogram shows the PCR products representing the 3’-cleavage fragments that were cloned and sequenced for each gene. Both *SlGRAS24* and *SlGRAS40* were cleaved between 10^th^ and 11^th^, 13^th^ and 14^th^ nt of mature miR171 sequence (arrows)

To uncover the evolutionary history of the GRAS gene family in tomato and to help in their classification, a total of 124 GRAS proteins, comprising 32 from *Arabidopsis*, 48 from tomato, 14 from *Prunus mume*, 14 from *Populus*, and 16 from Rice, were performed to construct an unrooted phylogenetic tree usingNeighbor-Joining (NJ) method by MEGA6.0 (Fig. 4). Based on the phylogenetic tree, the GRAS proteins could be divided into 13 subfamilies: AtPAT1, AtSCL4/7, AtSCL9, AtSHR, HAM, AtLAS, AtSCR, AtSCL3, AtSCL28,DELLA, Pt20, Os4, and Os19, agree well with the tree made by Liu et al. [9]. It is noteworthy that some GRAS proteins considered to be species-specific in previous publications have homologs in tomato. For example, 6 tomato *SlGRAS* genes (*SlGRAS21*, *SlGRAS22*, *SlGRAS23*, *SlGRAS31*, *SlGRAS33*, *SlGRAS33*), together with *PmGRAS20* and *PtGRAS20*, belong to “Pt20” subfamily, which was previously regarded as *Populus*-specific group [9]. Two (*SlGRAS27*, *SlGRAS28*) and one (*SlGRAS49*) tomato *GRAS* genes, were clustered into “Os4” and “Os19” subfamily, respectively, which were previously reported as rice-specific protein groups [9]. These three subfamilies did not include any *Arabidopsis* genes, implying lineage-specific gene loss in *Arabidopsis*. The other 10 subfamilies harbor *GRAS* genes from each of the five species with one to eleven *SlGRAS* genes per group. To date, the functions of the SlLS and SlDELLA protein have been clearly illuminated in tomato [25, 38–40]. AtPAT1 subfamily includes 11 members from tomato, two SlGRAS proteins (SlGRAS7 and SlGRAS12) and three SlGRAS proteins (SlGRAS1, SlGRAS14, SlGRAS32) have high sequence similarity with AtPAT1 and AtSCL13, respectively, which are associated with phytochrome A and B signaling, respectively [22], suggesting that tomato GRAS homologs might have similar functions in the phytochrome signal transduction. Six proteins (SlGRAS4, SlGRAS13, SlGRAS30, SlGRAS36, and SlGRAS42) are clustered into AtSCL9 subfamily. Although the biological roles of *Arabidopsis* GRAS proteins in this group are largely unknown, a member of this group in *Lilium longiflorum* named LiSCL was identified as transcriptional regulator during microsporogenesis [16]. Five (SlGRAS16, SlGRAS25, SlGRAS26, SlGRAS38, and SlGRAS39) and three (SlGRAS15, SlGRAS37, and SlGRAS43) proteins belong to AtSHR and AtSCR subfamily, respectively. Considering the important role of AtSHR and AtSCR proteins in root and shoot radial patterning [17], we predict these homologous genes in tomato may be related to root and shoot development. Two proteins (SlGRAS11 and SlGRAS18) belong to AtSCL3 subfamily, which regulates root cell elongation by integrating multiple signals in *Arabidopsis* [18, 19]. SlGRAS41 is the only member of AtSCL28 subfamily in tomato, and a homologous gene identified in rice, *OsGRAS29* (also known as *DLT*), is involved in controlling the plant height of by modulating brassinosteroid signaling [41]. There are 6 SlGRAS proteins (SlGRAS5, SlGRAS8, SlGRAS24, SlGRAS40, SlGRAS47, and SlGRAS 48) clustered into the HAM subfamily. In *Arabidopsis*, 3 GRAS proteins of this group are post-transcriptionally regulated by miR171 (*AtSCL6*, *22*, *27*) [42, 43]. Here, the closest homologs of these *Arabidopsis* genes in tomato are the two genes, *SlGRAS24* and *SlGRAS40*, both having a putative binding site for Sly-miR171. Hence, 5’-RACE was performed to confirm their relationship. As expected, the 5’-RACE products of the predicted size to be generated from cleaved *SlGRAS24* and *SlGRAS40* template could be amplified. Subsequently, these products were cloned and the sequences of several independent inserts were determined. Sequencing results showed that the complementary sequences of each gene to Sly-miR171 mature sequence as well as the cleavage sites were exactly the same (Fig. 5). Interestingly, *in silico* analysis (http://plantgrn.noble.org/psRNATarget/) showed that another member of HAM subfamily, *SlGRAS8*, can also bind Sly-miR171 mature sequence and was predicted to be regulated through translational repression rather than mRNA cleavage, suggesting that a complicated regulatory mechanism of Sly-miR171 and its target genes in tomato.

In addition, to further explore the orthologous relationships of GRAS genes between tomato and other Solanaceae crops, 50 and 30 *GRAS* genes from potato (*Solanum tuberosum*) and pepper (*Capsicum annuum*), respectively, were selected to construct another phylogenetic tree (Additional file 4). We found that almost every member of *SlGRAS* genes (except for *SlGRAS17*) has its homologous gene(s) in either or both of potato and pepper genome, suggesting that the evolutional conservation and closer homology relationship among *GRAS* genes in closely related species.

### Expression analysis of *SlGRAS* genes in different tissues and organs

**Fig. 6:**
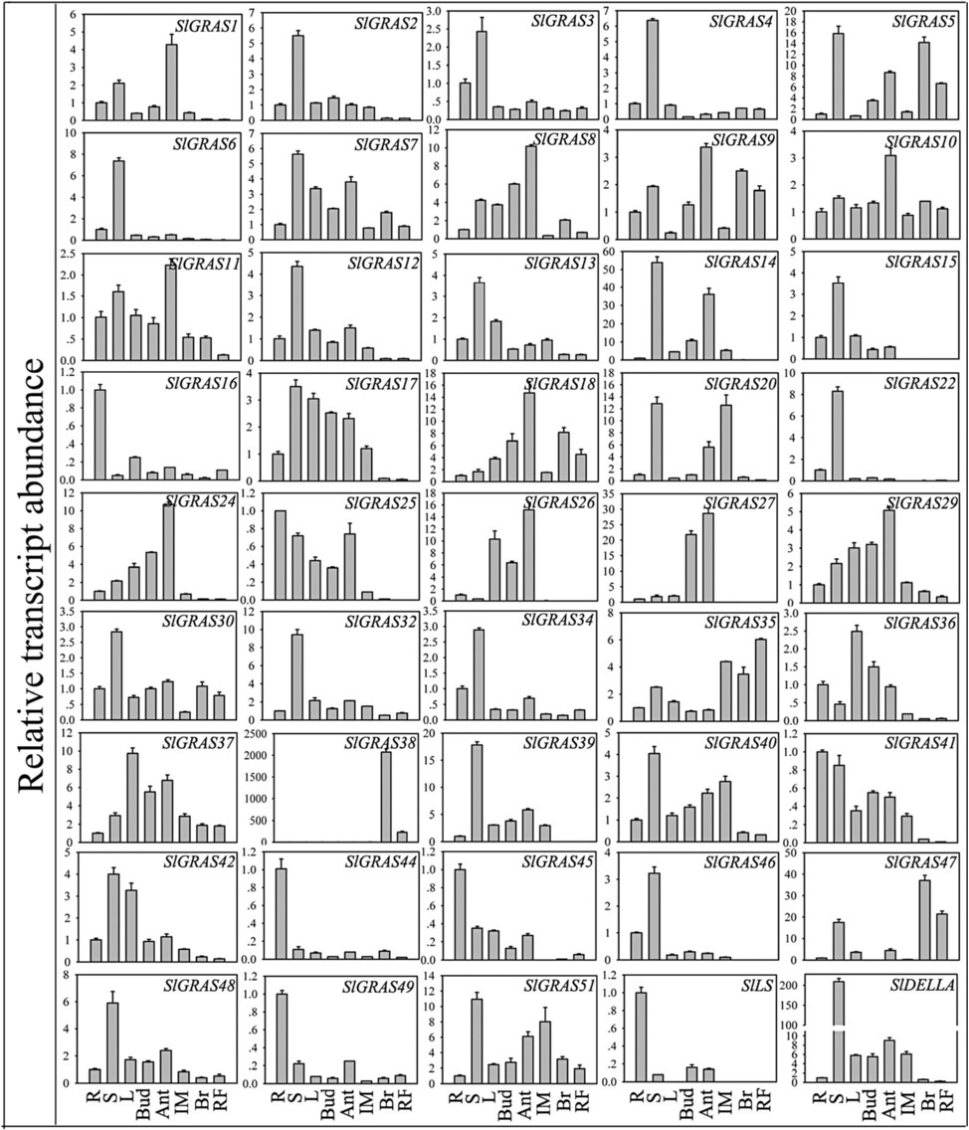
The expression profiles of 45 *SlGRAS* genes analyzed using qPCR during eight stages of development. Y-axis represents relative expression values and X-axis represents stages of development as follows: R root, S stem, L leaf, Bud bud flower, Ant anthesis flower, IM immature green stages, Br breaker stage, and RF red ripe stage of fruit development. The expression data of root were normalized to 1. Error bars show the standard error between three replicates performed

To investigate the potential functions of *SlGRAS* genes during tomato development, their expression patterns were carried out in different tissues including root, stem, leaf, bud, anthesis flower and three stages of fruit development using qRT-PCR. In the qPCR analysis, genes exhibiting Ct value > 36 were treated as non-expressors. As shown in Figs. 6 and 10a, a total of 45 *SlGRAS* gene transcripts were obtained, while 8 other *SlGRAS* genes could not be detected because of their low expression levels or might be pseudogenes. It is apparent that the expression levels in different tissues vary widely among the tomato *GRAS* genes, as well as among different tissues for individual *GRAS* genes. Of them, 23, 10, and 8 genes were found exhibit the highest expression in stems, anthesis flowers, and roots, respectively. During fruit development, generally higher transcript abundance can be observed in immature fruits than mature fruits, which suggests that those genes might relate to early fruit development. Nevertheless, several genes show dramatic increase at the breaker stage compared to the immature stage. For example, *SlGRAS38*, *SlGRAS35*, and *SlGRAS47* display relatively strong and specific expression during fruit ripening, indicating that they might have functional significance during the onset of ripening.

**Fig. 7:**
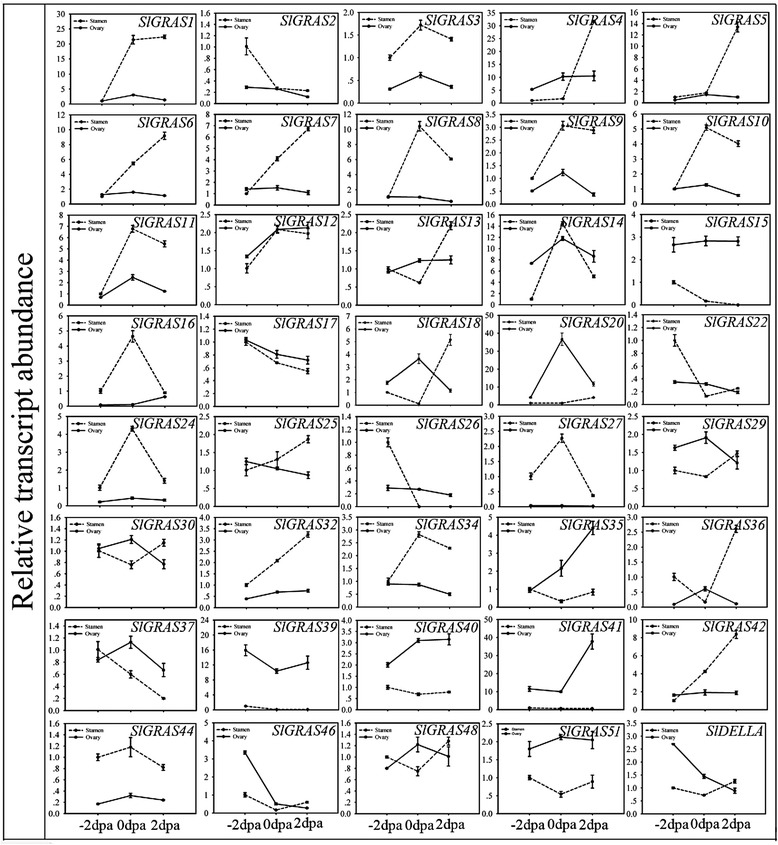
Expression patterns exhibited by 40 *SlGRAS* family genes during fruit-set stage of tomato. The X-axis represents 3 different stages, -2 dpa 2 days before anthesis, 0 dpa the first day of anthesis, 2 dpa 2 days post anthesis. Solid lines depict the expression patterns of ovaries while dotted lines stand for stamens. The expression data of -2 dpa stamens were normalized to 1. Error bars show the standard error between three replicates performed

A large number of *SlGRAS* genes demonstrate relatively high expression in flowers, suggesting the important role of these genes in such tissues. Given that many GRAS proteins are involved in regulating the gibberellic acid (GA) response, one of the key plant hormones during fruit set [44, 45], we analyzed the expression profiles of *SlGRAS* genes during the flower-to-fruit transition process (Figs. 7 and 10b). Of all the 40 *SlGRAS* genes identified, 16 genes exhibite higher expression in stamen while the transcripts of 12 genes are more abundant in ovary tissues, indicating functional specialization among GRAS gene family members in tomato floral organs, at least in stamen and ovary. The data show that most of *SlGRAS* genes undergo a drastic change in their mRNA levels either or both in stamens and ovaries, suggesting that the GRAS family members play different roles during pollination/fertilization.

**Fig. 8:**
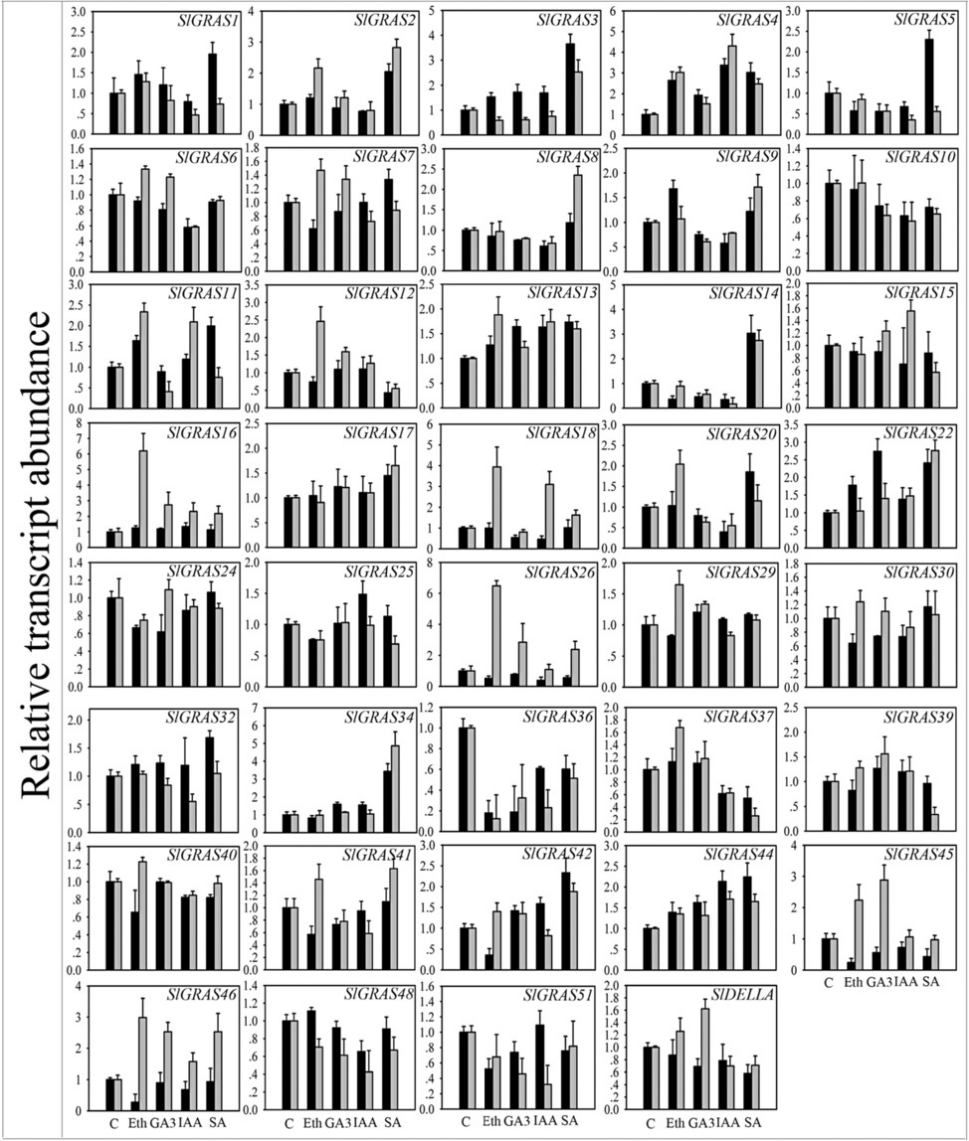
Expression analysis of 39 GRAS family genes in response to hormone treatments in two different parts of seedlings. Black and gray columns stand for the expression levels of the plant shoot part and root part collected from tomato seedlings, respectively. The X-axis represents various hormone treatments. C control sample, Eth ethephon, GA3 gibberellin, IAA indole acetic acids, SA salicylic acid. The expression data of control sample were normalized to 1. Error bars show the standard error between three replicates performed

### Expression analysis of *SlGRAS* genes in response to hormone treatments

Plant hormones have been extensively studied for their roles in the regulation of various aspects of plant development. In this study, hormone treatments resulted in a wide variety of *SlGRAS* gene expression profiles (Figs. 8 and 10c). The expression levels of 39 *GRAS* genes detected vary significantly in response to different hormone treatments as well as in different tissues in response to an individual hormone treatment, suggesting that the *SlGRAS* genes have differences in signal-selectivity not only among different hormones but also among different tissues of tomato seedlings. In ethephon (Eth) treatment, 15 and 12 *SlGRAS* genes were obviously induced and inhibited, respectively. Of them, the most up-regulated gene was *SlGRAS26* in roots, and the most down-regulated gene was *SlGRAS36* in shoots. Similarly, GA treatment led to 10 and 9 *SlGRAS* genes were obviously induced and inhibited, respectively, the most up-regulated gene was *SlGRAS26* in roots, while the most down-regulated gene was *SlGRAS36* in roots. In IAA treatment, 6 and 17 *SlGRAS* were significantly induced and inhibited, respectively, and *SlGRAS4* and *SlGRAS14* in roots were found to be most up- and down-regulated, respectively. As for SA treatment, 20 and 9 *SlGRAS* genes showed dramatic increase and decrease, respectively, *SlGRAS34* and *SlGRAS37* in roots went through the largest increase and decrease, respectively. Notably, several genes even demonstrated opposite expression in roots and shoots when responding to the same hormone treatment. For instance, *SlGRAS3* was up-regulated in shoots in response to Eth, GA3 and IAA treatments, while down-regulated in roots. Similar expression patterns were found in *SlGRAS18*, *SlGRAS26*, *SlGRAS41*, *SlGRAS45*, and *SlGRAS46*. The results suggest the complicated regulatory mechanism of these genes in response to hormone treatments in tomato. Taken together, these expression variations indicate that the SlGRAS gene family members were collectively regulated by a broad range of hormonal signals. Thus it is reasonable to speculate that those relevant genes might play pivotal roles in the cross-talk of hormones and should be candidates for further research in the field.

**Fig. 9:**
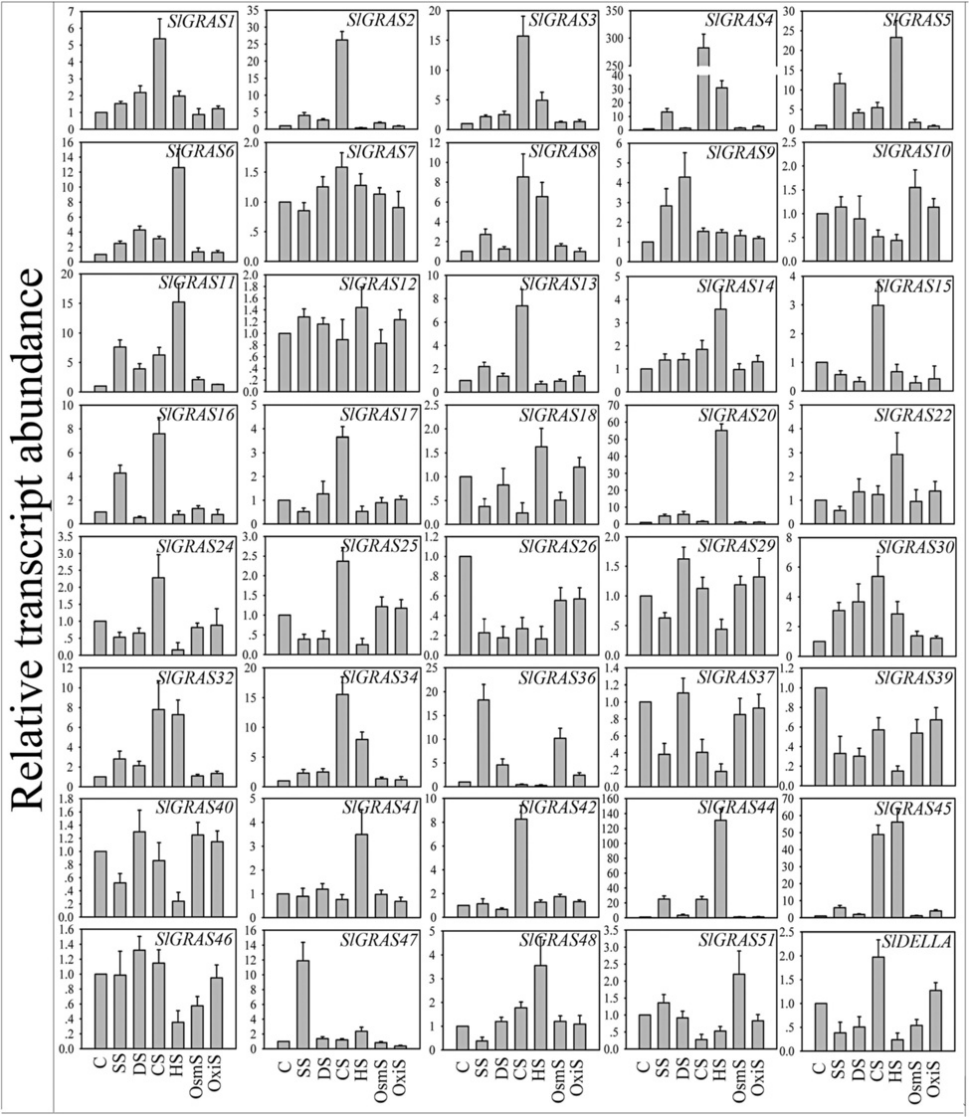
Expression analysis of 40 GRAS family genes in response to abiotic treatments. The X-axis represents different abiotic stresses. C control sample, SS salt stress, DS drought stress, CS cold stress, HS heat stress, OsmS osmotic stress, OxiS oxidative stress. The expression data of control sample were normalized to 1. Error bars show the standard error between three replicates performed

### Expression analysis of *SlGRAS* genes in response to abiotic treatments

**Fig. 10:**
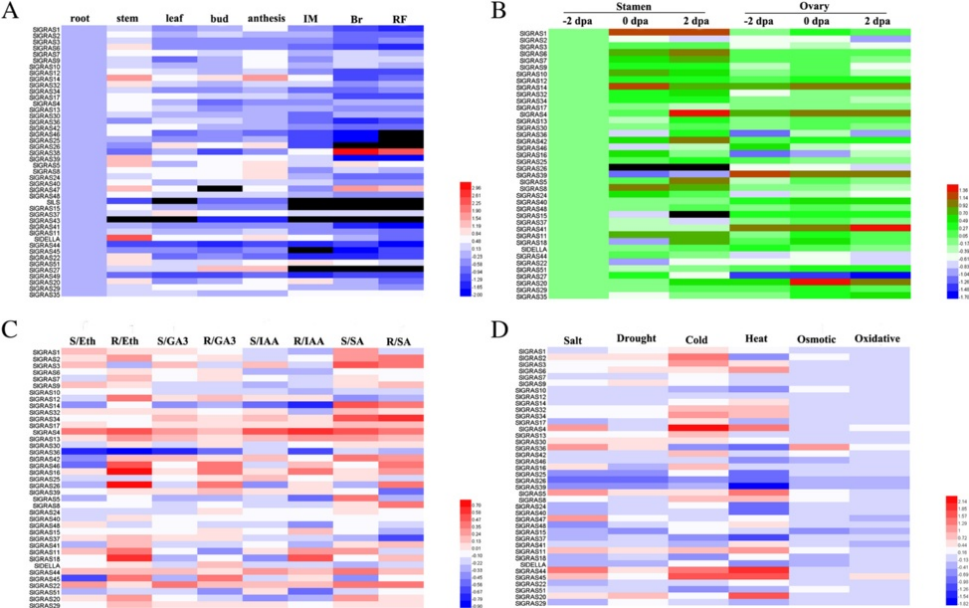
The expression profiles of *SlGRAS* genes visualized as heatmaps with respect to different tissues (**a**), floral organs (**b**), hormone treatments (**c**), and stress treatments (**d**). Members in the same subfamily based on the phylogenetic tree (Fig. 4) were grouped together. The color scale represents log10 expression values. The relative expression levels of root, -2 dpa stamen, and untreated control samples were normalized to 0

To further assess the functions of *SlGRAS* genes that may be involved in plant defenses to abiotic stresses, we analyzed the expressions of *SlGRAS* genes in response to salt, drought, cold, heat, osmotic and oxidative stress (Fig. 9 and 10d). Although only two genes (*SlGRAS26*, *SlGRAS36*) were found to be hyperresponsive to all treatments, all the analyzed genes exhibited differential expression in response to at least one abiotic stress treatments. Overall, in the six stressed conditions, a total of 30 *SlGRAS* genes were significantly induced, implying their putative roles in stress tolerance. 18 and 13 *SlGRAS* genes showed obviously increase and decrease under salt treatment, among which, *SlGRAS44* and *SlGRAS26* underwent the greatest mRNA levels change, respectively. Similarly, 17 genes were up-regulated and 7 genes were down-regulated following drought treatment with greatest change in *SlGRAS20* and *SlGRAS26*, respectively. Intriguingly, most members showed strong sensitivity toward heat (30 *SlGRAS* genes) and cold treatments (27 *SlGRAS* genes), and the largest differential expression usually observed when responding to one of these two treatments. For example, the transcript accumulation of *SlGRAS4* exhibited more than 250-folds change during cold stress compared to that in the control plants and *SlGRAS20* showed more than 50- folds change in response to heat treatment. By contrast, not too much change observed in most *SlGRAS* genes when osmotic and oxidative stresses were carried out. These data show the potential of some *SlGRAS* genes for enhancing adversity resistant capacity, especially considering that the tomato is an extremely temperature-sensitive crop.

## Discussion

To date, several attempts have been made to group members of GRAS family into subfamilies that reflect their evolutionary relationships [6–12]. These dendrograms were in substantial agreement though some fine-tunings. The bioinformatic analysis of GRAS proteins showing higher similarity within the same species indicates that gene duplications have occurred after the split among these lineages. Compared to *Arabidopsis*, larger number of GRAS proteins arisen in tomato suggests more gene duplications events or higher frequency of the retaining copies after duplication in tomato. Taken the tandem duplication events as example, 2/34, 10/45, 15/53, 17/60, 40/106 *GRAS* genes were identified as tandem duplicated genes in *Arabidopsis* [6], *P.mume* [11], tomato (Fig. 2), rice [6], and *Populus* [9], respectively, further validating that the duplication events are the most common mechanism contributing to the rapid expansion of GRAS gene family members in different species. Meanwhile, the exon-intron organization analysis showed that 77.4 % of *SlGRAS* genes were intronless in tomato (Fig. 1), with proportions 82.2 %, 67.6 %, 55 % and 54.7 % in *P.mume*, *Arabidopsis*, rice and *Populus*, respectively [9–12]. The high percentage of intronless genes in GRAS gene family in plant implies the close evolutionary relationship of GRAS proteins. Apart from GRAS gene family, intronless genes are also enriched in some other large gene families, such as F-box transcription factor gene family [46], DEAD box RNA helicases [47], and small auxin-up RNAs (SAUR) gene family [48]. Generally, intronless genes are archetypical in the prokaryotic genomes, and there are three explanations for the formation of the intronless genes in eukaryotic genomes: horizontal gene transfer from ancient prokaryotes, duplication of existing intronless genes, and retroposition of intron-containing genes [49]. Zhang et al. [50] recently reported the origin of plant *GRAS* genes from prokaryotic genomes of bacteria by horizontal gene transfer. That might be the reason of the abundant intronless genes in the GRAS gene family, which is likely to be its prokaryotic origin followed by extensive duplication events in the evolutionary history.

Intrinsically disordered proteins (IDPs) are highly abundant in eukaryotic proteomes and important for cellular functions. An IDR (intrinsically disordered region) within an IDP often undergoes disorder-to-order transitions upon binding to various partners, allowing an IDP to recognize and bind different partners at various binding interfaces [8, 51, 52]. By computational and bioinformatics tools, Sun *et al*. [8] demonstrated that the GRAS proteins are intrinsically disordered. One of the distinguishing features of GRAS proteins is its variable N-terminal, which is predicted to contain MoRFs (molecular recognition features), short interaction-prone segments that are located within IDRs and are able to recognize their interacting partners by undergoing disorder-to-order transitions upon binding to these specific partners [51, 52]. In tomato, except a few uncanonical GRAS proteins, multiple sequence analysis of tomato GRAS proteins showed that most members in this family possess a highly variable N-terminal domain, indicating the functional versatility of this gene family in tomato. Highly conserved C-terminal domains (GRAS domain) were observed in most SlGRAS proteins. Generally, Leucine-rich regions I (LR I) and II (LR II) flank the VHIID motif to form a LR I-VHIID-LR II pattern present in most GRAS proteins. It has been widely and experimentally confirmed for many GRAS proteins that the LRI-VHIID-LRII pattern or individual motifs within the pattern are used for interactions with protein partners [17, 23, 51–54].

Due to the functional diversity of *GRAS* genes, many members of this gene family need to be further functionally characterized. The expression patterns of *SlGRAS* genes here could help us to assess their possible functions. 8 *SlGRAS* genes were undetectable in any tissues/organs suggests a tendency to degenerate those genes after gene duplication or the lost of their functions during evolution. On the whole, the expression patterns vary greatly among different members even between those orthologous pair genes (*SlGRAS1* and *SlGRAS32*, *SlGRAS11* and *SlGRAS18*, *SlGRAS42* and *SlGRAS46*) (Fig. 6). Previously, expression profiles of *GRAS* genes in *Populus* and *P.mume* also demonstrated rather broad expression patterns across a variety of tissues, not only among subfamilies but members in the same clade [9, 11]. These results suggest that *GRAS* genes may undergo neo-functionalization or sub-functionalization in many higher plant species. Yet still, some GRAS genes with extremely high sequence identity (*SlGRAS1* and *SlGRAS14*, *SlGRAS2* and *SlGRAS3*, *SlGRAS7* and *SlGRAS12*, *SlGRAS9* and *SlGRAS10*) (Fig. 6) exhibited conserved expression patterns, implying their retention by genetic redundancy and selection for their contributions to the robustness of the genetic network. *SlGRAS25*,*SlGRAS39* and *SlGRAS15* with high mRNA levels in roots and stems suggests conserved functions with their homologous gene *AtSHR* [17] and *AtSCR* [55], which are involved in root and shoot radial patterning in *Arabidopsis*. The strong ovary-preferential expression of *SlGRAS41* during flower-fruit transition suggests its potential role in fruit development by modulating brassinosteroid signaling [45]. The homologs of *AtSCL3* (*SlGRAS11*, *SlGRAS18*) displayed high mRNA levels in anthesis flowers, indicating that they may exert new functions during pollination/fertilization by modulating GA signaling [18, 19]. Our results have proved that *SlGRAS24* and *SlGRAS40* can be cleaved by miR171 (Fig. 5), one of the most conserved miRNAs in plants, suggesting that they may have similar functions with their homologous genes characterized in other species such as *Arabidopsis* [43]. However, the expression patterns of *SlGRAS24* and *SlGRAS40* in tomato are largely different, which suggests that the complicated and widespread functions of the miR171-GRASs regulatory networks in tomato. Noticeably, according to the Supplementary Table 75 of Tomato Genome Consortium [27], there are 14 *SlGRAS* genes (*SlGRAS1*, *SlGRAS2*, *SlGRAS8*, *SlGRAS9*, *SlGRAS12*, *SlGRAS13*, *SlGRAS14*, *SlGRAS17*, *SlGRAS18*, *SlGRAS24*, *SlGRAS32*, *SlGRAS38*, *SlGRAS40*, *SlGRAS48*) were differentially expressed from mature green stage fruits to breaker stage fruits. Our results are consistent with the above data, suggesting the pivotal roles of these genes during fruit ripening. Two of them, SlGRAS18 and *SlGRAS38*, predominantly expressed in breaker and red ripening stage fruits, have been reported as target genes of RIN [56, 57], which is key transcriptional regulator during fruit ripening. Moreover, the spatio-temporal expression patterns revealed that the majority members of *SlGRAS* identified presented sharply increase or decrease upon pollination/fertilization either or both in stamen and ovary (i.e., *SlGRAS8*, *SlGRAS11*, *SlGRAS14*, *SlGRAS16*, *SlGRAS18*, *SlGRAS20*, *SlGRAS24*, *SlGRAS27*, *SlGRAS36*) (Fig. 7), indicating their potential active roles during ovary and anther development. Considering the relationship between *GRAS* genes and GA signaling, we speculate that members of this gene family involve in mediating GA responses during flower-to-fruit transition.

Plant growth and development are regulated by a chemically and structurally diverse group of hormones. Many known growth and development responses to hormones are due to modulation of gene expression, and these responses are among the best characterized to date [58]. In general, hormones control the expression of genes by regulating the abundance of two types of gene regulatory proteins, transcription factors and transcriptional repressors. To our knowledge, the relationship between GRAS proteins and hormones remain scarce, apart from the widely known gibberellin [3, 54], only a few reports mentioned some members involved in auxin and brassinosteroid signal transduction [41, 59, 60]. Among four hormones conducted here, auxin (Indole 3-acetic acid, IAA) is involved in almost all aspects of plant growth and development, from embryogenesis to senescence, from root tip to shoot tip [61]. Gibberellic acid also regulates a diverse array of developmental processes such as seed development and germination, organ elongation and control of flowering time [62]. Ethylene and salicylic acid play important roles in biotic stresses [63], while ethylene is also the key regulator during fleshy fruit ripening [45]. It has been reported that BnSCL1, a GRAS protein identified in *Brassica napus*, showed differential dose response to auxin in shoots and roots [59]. The current results demonstrated that the majority of *SlGRAS* genes detected here displayed distinct changes following different hormone treatments, and some of them even exhibited opposite trends in roots and shoots, suggesting that GRAS transcription factors regulate gene expression by modulating phytohormone signaling through complicated networks (Fig. 8). Additionally, several studies have revealed that *GRAS* genes play potential regulatory roles in stress responses. *PeSCL7*, a member of *GRAS* genes from *poplar*, was regarded useful for engineering drought- and salt-tolerant trees [64]. Over-expression of a *BnLAS* gene in *Arabidopsis thaliana* could increase its drought tolerance [65]. The DELLA protein was proved to be involved in many abiotic stresses such as low temperature, phosphate starvation, and high NO concentration [66–68]. As for the evidence of GRAS proteins in the regulation of plant defence responses in tomato: transcripts corresponding to *GRAS* genes in resistant tomato plants infected with virulent phytopathogenic bacteria were different [69, 70]. Furthermore, the expression analysis by qRT-PCR showed that several tomato *GRAS* genes were associated with plant disease resistance and mechanical stress response [36]. Many transcription factor families have been shown to display stress-responsive gene expression with significant overlap in response to various stress treatments, indicating the cross correlation upon signaling pathways involved in various stresses. The induction of *SlGRAS* genes in response to more than one stress treatments in the present work highlights the wide involvement of *GRAS* genes in environmental adaptation (Fig. 9). We observed that *SlGRAS* genes showed larger accumulation under salt, cold, and heat treatments compared to other three treatments, suggesting that the *SlGRAS* gene family members might play more important roles in response to these three stress conditions. Combined analysis of all qPCR data (Figs. 6, 8, 9 and 10), we found that four highly homologous genes belonging to AtPAT subfamily (*SlGRAS2*, *SlGRAS3*, *SlGRAS7*, *SlGRAS34*) exhibit similar expression levels when responding to hormone and abiotic treatments, implying that these genes may be also involved in hormone signaling and stress response. Consistently, two genes of this subfamily from rice, *CIGR1* and *CIGR2*, were reported to be gibberellin and stress related [71]. *SlGRAS36* was significantly decreased in response to all hormone treatments while obviously increased in its mRNA levels upon four abiotic stresses. Likewise, *SlGRAS4* was induced by all hormone treatments, and the strong upregulation of its transcripts under cold stress suggests the great potential for cold stress tolerance. Interestingly, both SlGRAS36 and SlGRAS4 share strong sequence similarity to AtSCL14, a GRAS transcription factor that is essential for the activation of stress-inducible promoters [41]. A homologous gene of *AtSCL14* from rice, *OsGRAS23*, is involved in drought stress response through regulating expression of stress-responsive genes [72]. Thus, we deduce that SlGRAS36 and SlGRAS4 may play important role in eliciting stress responsive genes in tomato. Besides, several *SlGRAS* genes were dramatically regulated under both hormone and abiotic stress treatments, indicating the coordinate response of these two determinants.

## Conclusions

Although some classical functions of GRAS transcription factors have already been characterized in several plant species, more members of the GRAS family in agricultural crops, especially in those with fleshy fruits, remain to be further studied. In this work, 53 GRAS transcription factors were indentified in tomato. The information generated about the structure of SlGRAS proteins will shed light on their functional analysis. The comparative, phylogenetic, and expression analyses of GRAS members will be useful to comprehensive functional characterization of the GRAS gene family, and to better understanding their possible roles in mediating hormone cross-talk and abiotic stress. After all, the data shown here should be taken into consideration in future studies for genetic improvements of agronomic traits and/or stress tolerance in tomato and probably other Solanaceae plants.

## Methods

### Plant materials and growth conditions

Tomato plants (*Solanum lycopersicum* cv. Micro-Tom) were grown on soil in greenhouse with suitable conditions: 14/10 h light/dark cycle, 25/20 °C day/night temperature and 60 % relative humidity, and the plant nutrient solution were irrigated once per week. Roots, stems, and leaves were collected on two-month-old plants, flowers (bud, anthesis) and fruit (immature, breaker stage, and red fruit) were harvested at the proper time. Stamens and ovaries were collected 2 days before anthesis (-2 dpa), the first day of anthesis (0 dpa), and 2 days post anthesis (2 dpa), respectively. All tissues were collected from six well-grown plants between 9:00 a.m. and 10:00 a.m. and thoroughly mixed, then frozen in liquid nitrogen immediately, and each tissues/organs were sampled for three independent times.

### Identification of tomato *GRAS* genes

At first, we used “GRAS” as a key word and the *S.lycopersicum* genome was chosen as initial queries, a total of 54 putative GRAS genes were obtained from the Phytozome database (http://www.phytozome.net). Meanwhile, systematic BLAST homology searches using amino sequence of the 32 AtGRAS proteins obtained from the National Center for Biotechnology Information (NCBI) were performed on all sequences in the International Tomato Annotation Group Release 2.4 tomato proteins (2.40) (BLASTP, *E* value ≤ 1 × 10^-5^) and tomato WGS chromosomes (2.50)(TBLASTN, *E* value ≤ 1 × 10^-5^) (SGN http://solgenomics.net/tools/blast/). Taken together, 53 potential *GRAS* genes were identified from the currently available genomic databases. Subsequently, online bioinformatics tools, ExPASy-PROSITE (http://prosite.expasy.org/) and TBLASTN of NCBI (http://blast.st-va.ncbi.nlm.nih.gov/Blast.cgi?PROGRAM=tblastn&PAGE_TYPE=BlastSearch&LINK_LOC=blasthome) were used to further confirm the presence of GRAS domain in resulting sequences.

### Bioinformatic analyses of tomato *GRAS* genes

The 53 putative *GRAS* genes were renamed according to their chromosomal location except 19 members were kept as their already existed name (*SlGRAS1*-*SlGRAS17*, *SlDELLA*, *SlLS*). The functional domain distribution and exon-intron structures of the GRAS proteins were obtained from Phytozome (http://www.phytozome.net). The tandemly duplicated genes were defined as an array of two or more *SlGRAS* genes with Smith-Waterman alignment e values ≤1 × 10^-25^ in the range of 350-kb distance, as proposed by Lehti-Shiu et al. [73]. We downloaded the GRAS protein sequences of *Populus*, rice, and *P.mume* according to two previous publications [11, 12], and at least one gene of each subfamily was selected based on the phylogenetic trees. Then, together with all 32 *Arabidopsis* AtGRAS proteins and 48 tomato SlGRAS proteins, the multiple sequence alignment were performed using the ClustalX2.0 program using the default settings. A phylogenetic tree based on the alignment was constructed using MEGA6.0 by the NJ (neighbour-joining) method with the bootstrap test replicated 1000 times.

### Modified 5’-RACE to identify the slicing sites of *SlGRAS24* and *SlGRAS40*

The 5’-Full RACE kit (TaKaRa, JAPAN) was used for RNA ligase-mediated rapid amplification of cDNA ends (RLM-RACE) assay according to the manufacture’s specification. Briefly, total RNAs were extracted from the seedlings of wild-type tomato, and Poly(A) mRNA was directly ligated to the 5’-RACE adaptor (60 nucleotides). The oligo(dT) primer was used to prime cDNA synthesis with reverse transcriptase. PCR was performed according to Tm of each GSP primers (Additional file 5), which were designed at the predicted 3′ products of complementary site of mature miRNA sequence. Finally, the PCR products were purified, cloned into pEASY-Blunt Cloning vector (Transgene) and were sent to sequencing for each product.

### Hormone and abotic stress treatments

For hormone treatment, 12-day-old tomato seedlings were soaked in liquid MS medium with 20 μM ethephon (Eth), 20 μM gibberellin (GA3), 20 μM indole acetic acid (IAA), 20 μM salicylic acid (SA) for 3 h, respectively. Roots and shoots of treated samples were harvested separately. Seedlings soaked in liquid MS medium without any hormone were used as control.

About one-month-old tomato plants were subjected to various abiotic stress treatments. For cold or heat treatment, tomato plants which were grown in green house were transferred to a cold chamber maintained at 4 ± 1 °C or in an incubator at 42 ± 1 °C, respectively. Salt, osmotic and oxidative stress treatments were carried out by spraying leaves with 200 mM NaCl, 100 mM mannitol and 100 mM hydrogen peroxide. Leaves were sampled at 6 h post treatment and untreated plants were used as controls. The drought treatment consisted of withholding water for up to 15 days. Well-watered plants were maintained as controls by watering plants daily.

At each treatment, materials from six separate seedlings/plants were combined to form one sample, and all of the treatment experiments were performed in three independent times. All these samples were frozen in liquid nitrogen immediately and stored at -80 °C until RNA extraction.

### RNA isolation and real-time quantitative PCR analysis

Total RNA was extracted using TRIzol reagent (Invitrogen, USA) according to the manufacturers’ instruction. RNA integrity was verified by 1.2 % agar gel electrophoresis and the RNA concentration was measured using NanoDrop 1000 (Thermo, USA). The PrimeScript^™^ RT reagent Kit with gDNA Eraser (TaKaRA, JAPAN) was used to remove any genomic DNA contamination and the first strand cDNA synthesis following the manufacturers’ protocol. Approximately 2 μg of RNA was used for each 20 μL reaction. Real-time quantitative PCR was conducted using SsoAdvanced^™^ Universal SYBR Green Supermix (BIO-RAD, USA) on a CFX96 Touch^™^ Real-Time PCR Detection System (BIO-RAD, USA). Each reaction mixture contained 10 μl SYBR Green Supermix, 1 μl cDNA template, 0.5 μl each primer, and 8 μl sterile distilled H_2_O. The PCR amplification cycle was as follows: 95 °C for 30 s, 40 cycles at 95 °C for 5 s, and 58 °C for 20 s. Melting curve analysis was performed ranging 60 to 95 °C to verify the specificity of the amplicon for each primer pairs. Relative fold differences were calculated based on the comparative Ct method using the 2^-△△Ct^ method with the *SlUBI* as an internal reference gene. All the primers for qPCR were designed based on the reference sequence obtained from the tomato WGS chromosomes 2.50 (Additional file 5).

## Availability of supporting data

Phylogenetic data (alignments and phylogenetic trees) supporting the results of this article have been deposited in TreeBASE respository and is available under the URL http://purl.org/phylo/treebase/phylows/study/TB2:S18045.

## Additional files

Additional file 1:
**The nucleotide and protein sequence information of all 53** ***SlGRAS*** **genes.** The amino acid sequence of GRAS domain of each protein was indicated in *red*. (PDF 372 kb) Additional file 2:
**Tandem duplication events in the 53** ***SlGRAS*** **genes.** (PDF 111 kb) Additional file 3:
**Multiple sequence alignment showed the other 4 most prominent motifs of GRAS domain: LR I, LR II, PFYRE, SAW, respectively.** (PDF 202 kb) Additional file 4:
**Phylogenetic analysis of GRAS proteins of tomato, potato, and pepper.** The phylogenetic tree was generated by Neighbor-Joining method derived from ClustalX alignment of 48, 50, and 30 GRAS amino acid sequences from tomato, potato, and pepper, respectively. (PDF 291 kb) Additional file 5:
**Primers used in this work.** (PDF 159 kb)
