# Supplementary material for: Genome-wide identification, phylogeny and expression analysis of GRAS gene family in tomato
Source: BMC Plant Biol. 2015 Aug 25;15:209. doi: 10.1186/s12870-015-0590-6 (PMC4549011; doi:10.1186/s12870-015-0590-6)
Supplement: Additional file 1: — The nucleotide and protein sequence information of all 53 SlGRAS genes. The amino acid sequence of GRAS domain of each protein was indicated in red. (PDF 372 kb) [file 12870_2015_590_MOESM1_ESM.pdf]

Additional file 1: The nucleotide and protein sequence information of all 53 *SlGRAS* genes. The amino acid sequence of GRAS domain of each protein was indicated in *red*.

SlGRAS1 Solyc11g012510.1.1

Nucleotide sequence (ORF):
ATGCAAACATCTCAGAGAACACAAATGTCTGGAAGTGTTCACGGATTGTACAATCAGCAAATGCAGCAAG
TTGAGCAGTATTACGCCCCTTACGATGTTCTTAAGAACAGCTGTAAGGATAATCGTAGCTCAGGGATGCA
ATTCTCTTTACAGGCACAAGACGAACAATTCTTCACGCTGGACTCGTCACCAGCTACCGACTATGTTGTCA
ACGATTCACCTCCTGCCTTGAGTGTTTCTTCCAACAGGAGTCCCTTTTCTCCTCAATGTTCACAGTCATACAT
GTCTGATCTGCATCACTCCTCTGATAACACGTGTGGTTCGCCTTTGAGTGGGTGTTCAGGAATTGATGATG
GTGACTTGAGACATGTGCTTCGGGAGCTGGAGAATAAATTACTGGGGCCTGAATCTGATACTGACGACA
GCTGCAGTTGCTCCTTGAACGATATGGTCTCAAAACCGTCTTCTGTTACAAGGTGGAACCGAGTGTTGGAC
ATGGCCCCTGGCTTGAACTTGAAAGAGTTGCTCGATGCCTGTGCTGAAGCAGTGTCAGATGCTGATATCT
CTACTGCTGAAGCTCTTATGAGTGCTTTAGAGCAACGGGTGTCTGTGTCTGGGGAACCGATGGAACGATT
GGGTGCATACGTGTTGGAAGGGATTAGAGCACGACTACTATCGTCAGGAAGCATCATATATAAAAAATTG
AAGTGCAAAGAACCAACTGGCTTGGAACTATTGTCTTACATGCAAGTCATCTTTAACATGTGTCCATACTA
CAAATTTGCTTATATGTCTGCGAATGTCGTCATTAATGAAGCCATGATGAATGAAAACAGAATCCATATCA
TTGATTTTCAGATTGCACAGGGAAGTCAGTGGATGTTCCTCCTCCACTATCTTGCTCATCGTCCTGGTGGAC
CCCCATTTGTCCGCATCACAGGTGTTGATGACGACGAATCAGCTTATGCTCGGGGTGGAGGACTTCAGCT
AGTAGGCAAAAGGCTAGCAGAAGTTGCCAAATCATGTGGAGTGCCTTTTGAATTCCATGGTGCTGCACTA
TCAGGCTGTGAGGTCCAACTAGAGAACCTTCGGGTTAAACATGGAGAAGCTCTGGCAGTAAACTTCCCTT
ATATGCTGCACCACATGCCAGACGAGAGTGTAAGTACTATCAATCATCGTGACCGCTTATTAAGACTAGTT
AAGAGTTTATCCCCCAAAATTGTGACCTTAGTTGAACAAGAATCAAACACCAACACCGCCCCTTTCCTTCCA
AGGTTCCGTGAAACTCTTGATTACTATACAGCAATGTTCGAATCAATTGATGCAGCTCGTCCTAGGGATGA
CAAGGAGAGGATCAGTGCAGAGGAGCATTGTGTGGCACGAGATGTTGTGAACATAATAGCATGTGAGG
GAGCTGACAGAGTGGAAAGACATGAACTTTTCGGAAAATGGAGGTTAAGACTTATGATGGCTGGATTTA
CTCAATGTCAATTGAGTCCATCAGTTGGTGAGACCATAAAGCACATGTTGAAAGAGTACAGCCCTAATTAT
CGGTATGCAGAAGGCGAAGGAGCACTCTATCTTGGATGGAAAAATAGAGCTTTAGCAACTTCCTCTGCCT
GGAGATGA

Amino acid sequence:
MQTSQRTQMSGSVHGLYNQQMQQVEQYYAPYDVLKNSCKDNRSSGMQFSLQAQDEQFFTLDSSPATDYVV
NDSPPALSVSSNRSPFSPQCSQSYMSDLHHSSDNTCGSPLSGCSGIDDGDLRHVLRELENKLLGPESDTDDSCS
CSLNDMVSKPSSVTRWNRVLDMAPGLNLKELLDACAEAVSDADISTAEALMSALEQRVSVSGEPMERLGAYVL
EGIRARLLSSGSIIYKKLKCKEPTGLELLSYMQVIFNMCPYYKFAYMSANVVINEAMMNENRIHIIDFQIAQGSQ
WMFLLHYLAHRPGGPPFVRITGVDDDESAYARGGGLQLVGKRLAEVAKSCGVPFEFHGAALSGCEVQLENLRV
KHGEALAVNFPYMLHHMPDESVSTINHRDRLLRLVKSLSPKIVTLVEQESNTNTAPFLPRFRETLDYYTAMFESID
AARPRDDKERISAEEHCVARDVVNIIACEGADRVERHELFGKWRLRLMMAGFTQCQLSPSVGETIKHMLKEYS
PNYRYAEGEGALYLGWKNRALATSSAWR-

SlGRAS2 Solyc07g063940.1.1

Nucleotide sequence (ORF):

ATGGAATCACACAATCTGTATGGATATGGTGCGACTGATGTGAATTTGCCATATTCTTACTCTATGACTACT
ACCCCTTCGATACCTAATAGGCTGTTGGGAATATTGAAATTTGATTCGGGAAATTCTCCGAATTCTCCATTT
GCAAACTATAATGATCCTGGTACCCCTACCACATTAAGTGATAGCCTGGAGCAACACAGCTCTACTGAAAA
TATTTCAGGGACTAGTCCTTGCAGTAATTCCTCGCGGGATTATAATCATTATTTCTGTAGGCCTAGTCCCTC
TCCAGATATCCACCATAACAATCTTCTGGTTTATTCTGGTGATAATTCTTTACTCCAGTATGCAAATCACAG
TCACAACATGAAACATGCGCTGCTTCAATTAGAGACCGCTTTAATGGGGCCAGAAGAAGTCACAACATCC
AGCCCCTCTGCAGGGGAAATTCAGCAGCCACAAGCATCAGGTGGTCAGAGGTCCGGTATGTGGCACCAA
GATGGCCAGGTTCCATATCGAATTGAAACACAGCCATCACATGTTTCTGTTTTTGGCATATCAGGGGATAT
AATTCAAAGTGAGGAGCATCACAAACCGATGGTGGATTATCCGTCGCAGGGGATTCCTTTTGGCAACCTA
AAGGAGCTGCTTATCGCGTGTGCCAGAGCTCTTGCTGAGAACAACTTGGATGATTTTGAAAAATTGATTG
CCAAGGCAAGAAGTGCTGTGTCCATTACTGGTGATCCAATCCAGCGTCTCGGTGCTTACATCGTAGAAGG
GCTAGTAGCAAGAAAAGAAGCATCCGGAACCAACATATACAGGGCTCTCAGATGTAAGGAACCAGCAGG
CTGGGACTTGCTTTCCTACATGCATATCTTATATGAGATATGCCCTTATCTAAAGTTCGGTTACATGGCTGC
AAATGGTGCAATAGCAGATGCGTGTAGAAATGAAAATCGCATTCACATTATAGACTTCCAAATTGCACAA
GGGACTCAATGGCTGACTCTTCTTCAAGCTCTTGCGGCCAGGCCAAGCGGTGCACCTTATGTACGTATTAC
AGGTATTGATGATCCAGTTTCAAAATACGCCCGAGGAGATGGATTGGCCGTAGTGGGAAAAAAGCTCGC
AGCAATTTCTGAGAAATTCAACATTCCTGTTGAGTTTCATGCTGTACCAGTTTTTGCTCCTGAAGTCACCAG
GGACATGCTTGATGTGAGGCCTGGTGAGGCTCTGGCAGTAAACTTCCCTTTGACACTTCACCACACACCTG
ATGAGAGCGTTGACGTGACTAATCCAAGGGATGAGCTTCTAAGGATGGTGAAGTCATTTTCTCCAAAGGT
GGTCACGTTGGTGGAACAAGAATCAAACACAAACACCGCCCCCTTTTTCCCTCGATTTCAAGAAGCTCTAG
ACTACTATTCTGCAATGTTCGAGTCCATAGATGTGACATTAGAAAGGGACAGGAAGGAAAGAATCAACGT
GGAGCAGCATTGTTTGGCAAGGGATATAGTTAACGTCATAGCATGTGAGGGCATGGAAAGAGTGGAACG
CCACGAGCTATTAGGGAAATGGAAATTGAGGTTCACAATGGCAGGGTTCCACCAGTATCCTCTGAGCTCT
TACGTGAATTCAGTGATAAAGAGCCTCATGAGGTGCTACTCAGAACATTATACACTGGTGGAGAAAGATG
GAGCTATGTTGTTGGGATGGAAAAAGAGGAACCTAATCTCTGCATCAGCTTGGCACTGA

Amino acid sequence:

MESHNLYGYGATDVNLPYSYSMTTTPSIPNRLLGILKFDSGNSPNSPFANYNDPGTPTTLSDSLEQHSSTENISGT
SPCSNSSRDYNHYFCRPSPSPDIHHNNLLVYSGDNSLLQYANHSHNMKHALLQLETALMGPEEVTTSSPSAGEI
QQPQASGGQRSGMWHQDGQVPYRIETQPSHVSVFGISGDIIQSEEHHKPMVDYPSQGIPFGNLKELLIACAR
ALAENNLDDFEKLIAKARSAVSITGDPIQRLGAYIVEGLVARKEASGTNIYRALRCKEPAGWDLLSYMHILYEICPY
LKFGYMAANGAIADACRNENRIHIIDFQIAQGTQWLTLLQALAARPSGAPYVRITGIDDPVSKYARGDGLAVVG
KKLAAISEKFNIPVEFHAVPVFAPEVTRDMLDVRPGEALAVNFPLTLHHTPDESVDVTNPRDELLRMVKSFSPKV
VTLVEQESNTNTAPFFPRFQEALDYYSAMFESIDVTLERDRKERINVEQHCLARDIVNVIACEGMERVERHELLG
KWKLRFTMAGFHQYPLSSYVNSVIKSLMRCYSEHYTLVEKDGAMLLGWKKRNLISASAWH-

SlGRAS3 Solyc12g005340.1.1

Nucleotide sequence (ORF):

ATGGACTCGCACCATTTGGTTGCTTATGGTGTGACCGGTAGTGATTTGTCGTATTCTTCATGCCCTACTGTT

AGTCCTCTAGAGAATAGACCATTTGGTACATCGAAATTTGATTCAGGAAATTCGCCACTTGTGAACTATTT
TAACTCTGAGACCTTCAACACAGTAAGTGATTACCAGGAGCAGCCCAGCTGTACAGAGAATCTTTCAGGT
GCTAGTTCTTCAAGTGGTTCCTCACTGGATTATAACCAATATTTCCATCGACCAAGCCCCTCGGAAGATCAT
CTTCCAGAAGCTCCTTACAGTCGGAACATGAAACATACTCTGCTGCAGTTGGAGTCTGCTTTAATGGGGCC
AGACAAAGAGGCAATGAAATCCAGCCCTTATCTTGGTGAAAATATGGGGGCACAAACATCAGGTCAGAG
GTATAAAGCGTGGAACAAGGAAGCCCAAGTTGTGCGTCATCAACAATCAGTAGTTTCAATCCTTAATGGA
ATTCAAAGTGATAAGCGAGACAATGTAATGGAGGACTTGCCCTTGCAGGGTGTTCCGTCTAGCAATCTGA
AGCAGCTGCTTATAGCATGCGCCAGAGCTCTTGCTGAAAACAAGCTAGATGATTTTGAAATACTGGTTGCT
AAGGCAAGGAGTGTTGTGTCCGTTACTGGAGATCCCATCCAGCGTCTTGGTGCTTACATTGTAGAAGGCC
TCGTAGCAAGAAAGGAGTTATCTGGAACTACCATATACAGGAGTTTAAAGTGTAAGGAGCCGGCTGGTA
AGGACTTGTTCTCCTACATGTATATCCTCTATGAAATATGTCCTTACCTAAAGTTCGGCTACATGGCTGCAA
ATGGTGCCATAGTAGAAGCATGCAGAAATGAAGATCGCATCCACATTATAGACTTCCAAATTGCACAGGG
GACCCAATGGATGACTCTCTTACAAGCTCTTGCAGCACGACCCGGTGGTGCCCCCTACGTACGTATTACAG
GAATTGATGATCCAGTTTCACAGTACGCTCGGGGAGATGGATTGGCTGCAGTTGCCAGACGGCTATCAGC
AATTTCTGAGGAATTCAACATTGCTGTTGAGTTTCATGCAGTGCCAGTTTTTGCTCCGGAAATCACTTGGG
ATATGCTTGATGTAAGGCCTGGTGAGGCTCTGGCTGTAAACTTTCCTTTGCAACTTCACCACACCCCTGAT
GAGAGCGTTGACGTGAATAACCCTAGAGATGGTCTCATTAGGATGATAAAGTCGCTTAGCCCCAAGATAG
TCACTTTGGTGGAGCAAGAATCAAACACGAACACAGCTCCATTTCTCCCTAGGTTTGTAGAAGCTCTAGAT
TACTACCATGCAATGTTTGAGTCCATAGATGTGACCCTACTAAGGGACATGAAGGAGCGGATCAATGTGG
AACAGCACTGTTTGGCTAGGGATATAGTGAATGTCATAGCATGTGAGGGCAAGGAAAGAGTAGAACGTC
ACGAGCTATTGGGGAAGTGGAAATCCAGGTTCATGATGGCAGGTTTTCAGCAATATCCTTTGAGCTCTTAT
GTGAATTCAGTGATTAAGGACCTCATGAAGCGTTACTCGGAGCATTATACACTGGTGGAGAAAGACGGA
GCTATGCTGTTGGGGTGGAAGGAGCGGAATCTAGTCTCTGCGTCTGCTTGGTTTTAA

Amino acid sequence:
MDSHHLVAYGVTGSDLSYSSCPTVSPLENRPFGTSKFDSGNSPLVNYFNSETFNTVSDYQEQPSCTENLSGASSS
SGSSLDYNQYFHRPSPSEDHLPEAPYSRNMKHTLLQLESALMGPDKEAMKSSPYLGENMGAQTSGQRYKAW
NKEAQVVRHQQSVVSILNGIQSDKRDNVMEDLPLQGVPSSNLKQLLIACARALAENKLDDFEILVAKARSVVSV
TGDPIQRLGAYIVEGLVARKELSGTTIYRSLKCKEPAGKDLFSYMYILYEICPYLKFGYMAANGAIVEACRNEDRIHII
DFQIAQGTQWMTLLQALAARPGGAPYVRITGIDDPVSQYARGDGLAAVARRLSAISEEFNIAVEFHAVPVFAPE
ITWDMLDVRPGEALAVNFPLQLHHTPDESVDVNNPRDGLIRMIKSLSPKIVTLVEQESNTNTAPFLPRFVEALDY
YHAMFESIDVTLLRDMKERINVEQHCLARDIVNVIACEGKERVERHELLGKWKSRFMMAGFQQYPLSSYVNSV
IKDLMKRYSEHYTLVEKDGAMLLGWKERNLVSASAWF-

SlGRAS4 Solyc01g100200.2.1

Nucleotide sequence (ORF):
ATGGAAGCCCTTTTTCAAGAACAGCTTTTTCCTTGTGCTGATTCTTTTATTTTTAGGCACCCTTCCATTCCAA
TGGATCCAAGAAAAGAAGTTATACAAAATGGACTCAATAATCATCCTAGTTTTGATCAAGATTACTTTTCA
AATCATGTTGTTGGAGTGGGAGATTCTTCTCCTCCTCCTCAAGAGGAAGGGGAAAAGGACTATTCTGATG
CAATGTACAAATTCTTAAGTCAGATGCTTATGGAAGAAGATGATTTGGAGAATAAGCCATGTATGTTTCAT
GACTGCATGGCTCTCCAAGCTAAAGAGAGATACTTATCTGATGTACTTCATGGATCCGAAAACAACTATTC
CCCACAATCTGTCATTATCAATCCACACGATTCCTCTAGTTTTCTCAGCAACTATTCACCTGATTCTATTGAG

TCACCTCAATGGGATCTTAATTTCGAATCCCCTGCTTCCATGTCCACTTTATCTAATCATGACTCTTTCTTCA
CTTCCTTCGGCAATGGCCATTTTGAAGAAGGAGCTGTCAATGTGTTACAAAGTGGCAGCAGCAGCAATTC
ACCAACTGGTCTGAGGGAAAAGAAAAATCGACATCGAGGAGATGTTGCTGCAGACCAGCAGAGGAGTA
ACAAACAGATGGCTACATTTGTTCATGATGAATCTGAACCATTGGAAATGTATGACAATGTGTTGCTTTGT
TTAAACAATCCATATGTGGAACAGCATAGTGCTACTAGTATCACTTCCTACTCGCCGCCTAATGAAGCCAA
AAAAACAAGCAAAGTTGGAAGGCCGAGAGGTGGTAGGAAGCATAGCAGCATTGTCAAGAAGGAAATGG
TGGATTTGAGGGCTTTGTTGACTCAGTGTGCGCAGGCCATGGCAAACTATGATAGCAGAACAGCTAATGA
GCTGCTGATGCGGATAAGAGAACACTCTACACCTCATGGCGATGGGACGGAGAGGTTGGCTCATTATCTT
GCCAATGCCCTTGAAGCACGCCTGTCCGGCACGGGAACAGCTTTGTATACAGCCTACGCACCCAGTAGGA
TATCAGCTGCTAACATTTTGAAAGCTTACAAGGCATTTATCAGAGCATGTCCATTCAAGTTGCTGTCAAAC
ATTTTCGCAAACAAGTATATCCGAAAGGTCATTGCTGGAGCACCAAAGATACACATAATTGATTTTGGTAT
CTTGTATGGTTTCCAATGGCCCTGTCTCATACAAGGTCTATCCATGAGGGCTGGGGGACCTCCAGAGCTTC
GCATTACTGGAATCGATCTTCCCCAGCCAGGTTTCAAGCCAGCAGGGAGGGTTGAGGAGACAGGGCGTC
GCTTGGAAAAGTACTGCAAGCGATTTAGTGTCCCTTTCGTATTCAAAGCCATCGCGAAGAAGTGGGAAAG
CATCACGCTTGAAGAGCTTGAGGTTCAAAGGGATGAAGTGTTGGTAGTTAACAGTTTGTATAGGCTAGGA
AACATACCTGATGAGACAGTAGTACCAAACAGTCCAAGGGATGCTGTCCTGAATTTAATCAGGAGGATCC
GTCCTGATTTATTCATCCATGGTGCGTTGAACGGTACTTTCAACACTCCATTTTTCGTCACACGATTCAGGG
AGGCGCTTTTTCACTTCTCTTCACTGTATGATATGTTTGAGGCTACTCTTCCCCGCGAGGACGAGGACAGA
AAGCTATTCGAGGAAGAGGTTTTTGCAAGAGATGCTATGAACGTGATTGCTTGTGAAGGGACAGAGAGA
GTTGAGAGACCTGAAACATACAAGCAGTGGCAACTTAGATGCGTTAGAGCTGGATTCAAACAGGTGCCA
CTTGACCAGGAGATTGTGAAGATAGTGAGGAATAAAGTGAGGTCGGAGTACCACAGGGACTTCTCAGTT
GATGAAGATGGACACTGGATGCTACAAGGATGGAAAGGACGCGTAATTTACGCTCTCTCTTGTTGGAAGC
CTACAAAGCAGTCTGTAAAATTAGTCTAG

Amino acid sequence:
MEALFQEQLFPCADSFIFRHPSIPMDPRKEVIQNGLNNHPSFDQDYFSNHVVGVGDSSPPPQEEGEKDYSDA
MYKFLSQMLMEEDDLENKPCMFHDCMALQAKERYLSDVLHGSENNYSPQSVIINPHDSSSFLSNYSPDSIESP
QWDLNFESPASMSTLSNHDSFFTSFGNGHFEEGAVNVLQSGSSSNSPTGLREKKNRHRGDVAADQQRSNKQ
MATFVHDESEPLEMYDNVLLCLNNPYVEQHSATSITSYSPPNEAKKTSKVGRPRGGRKHSSIVKKEMVDLRALL
TQCAQAMANYDSRTANELLMRIREHSTPHGDGTERLAHYLANALEARLSGTGTALYTAYAPSRISAANILKAYKA
FIRACPFKLLSNIFANKYIRKVIAGAPKIHIIDFGILYGFQWPCLIQGLSMRAGGPPELRITGIDLPQPGFKPAGRVE
ETGRRLEKYCKRFSVPFVFKAIAKKWESITLEELEVQRDEVLVVNSLYRLGNIPDETVVPNSPRDAVLNLIRRIRPD
LFIHGALNGTFNTPFFVTRFREALFHFSSLYDMFEATLPREDEDRKLFEEEVFARDAMNVIACEGTERVERPETYK
QWQLRCVRAGFKQVPLDQEIVKIVRNKVRSEYHRDFSVDEDGHWMLQGWKGRVIYALSCWKPTKQSVKLV-

SlGRAS5 Solyc09g018460.1.1

Nucleotide sequence (ORF):
ATGATTCAATCAGAGATTCTTCACAACTCATGGTCTTCAACTAACCAAATTCATCCTTTTTATGAATATCAAA
ATCATGTTATTTCTAATTATTCTTCACTTAACTCCAAAGATCAAGATTCCTCTGAAATTTCCTTATCAGATTTC
ACTTCCCTTTTCTCTGATGATGTTCTTAATGATCTCCCCATGGATGATATCGAGTTCGTTGATGTCTCTCGAT
GGTTAAACGATAGTGAAAATGAAGGAAATGAAAATACAAAAATCACTTCAGAGACTAAAAAAGATGGAG
ACTCATGTAGTCCTGCACTTTCAAGCATATCCATTGATACCTCAACGACAATACAGTCAAGAAATGATGTT

ATTTCACAAAGTAGAGAAATCGACAACCAAATGAGAATTCATCATTTATTGTCGGTGTATGGAGAGGCAA
TGGAAAATGGTCACAAAGAATTAGCAGAAGTGATCATACGATGCATAAAGGGGAAAATAAATCCTTTAG
GTGAATCGTTGGAACGCGTTGCTTCAAACTTATTCCAGCATACAGAAGAAGACGATCAAGAAAGTTACTT
GAAACAAGAATCAAACAAGATTATTGAAGCAGCATTCAAAGCCTTCTACCAAATTTTCCCATTAGGGAAAT
TTGCTCATTTTGCTGCTAACTCCGCAATTATAGAAGCTATGCCTGATGAGTCACAGACAATTCACATAATAG
ATTATGATATGGGAAAAGGGATTCAATGGCCTCCAATTATTAAAGCCATGAGCCAAAAAAGAAAGATTTT
GAGATTAACATCCATAAAGAAATCAACAAGTGATCATTGGAGATTTGAGGAAACAAAAAGAAGACTTGTT
GATTATGCAAATCTTTTTGGCCTAACATTACAAGTTGATGAGATGACATTTGAAGAATTGGCAAGTGAATT
GAGAAGAATGAAGAAAAGGGGTAAAAGGAGTGAATGGTTGGTTTTCAACTGTATGTTTCAATTTTCTTAC
ATGGGGAAAAGGATGCGTAGAATCGACGCGTTGGGGTTTCTGAAACTAGCTAAGGAGTTGTTATTAGCT
AATTCTTCAACACACAGAGGACTTGTCACTTTTGGCGATGGAGAAGCAATGGATAATAGTCATCATAACAC
AAACAATACTTTTTCTTCATACTTTAACAAGAATTTGTTACATTACCAATCGATTTTCGAGTCATTGGAATTG
TATTTTCCCTCTCATCTTGCACAAGCAAGAGTTGCCATGGAATCTCTTTTTCTAGCTCCTGAAGTGTGCTCAT
TTTCTTGGTTCCAAAACTGGGAAGAGATGACAAATGTTTCTGATTCTTGTAGTGAAATGGGGTTGCAAGG
TATGAGAATAAGCAAAGAAAACTTATTACAAGCCAAAGAAGTGGTGAATGAAAGAGAAACTCCATTTAA
GGTGAGGATTGAAGAAGAGAGGCAACATGAAATGGTCTTGGAATGGAAAGGCATATCTATTGTGAGAGT
TTCAACTTGGATGTAG

Amino acid sequence:
MIQSEILHNSWSSTNQIHPFYEYQNHVISNYSSLNSKDQDSSEISLSDFTSLFSDDVLNDLPMDDIEFVDVSRWL
NDSENEGNENTKITSETKKDGDSCSPALSSISIDTSTTIQSRNDVISQSREIDNQMRIHHLLSVYGEAMENGHKEL
AEVIIRCIKGKINPLGESLERVASNLFQHTEEDDQESYLKQESNKIIEAAFKAFYQIFPLGKFAHFAANSAIIEAMPD
ESQTIHIIDYDMGKGIQWPPIIKAMSQKRKILRLTSIKKSTSDHWRFEETKRRLVDYANLFGLTLQVDEMTFEELA
SELRRMKKRGKRSEWLVFNCMFQFSYMGKRMRRIDALGFLKLAKELLLANSSTHRGLVTFGDGEAMDNSHH
NTNNTFSSYFNKNLLHYQSIFESLELYFPSHLAQARVAMESLFLAPEVCSFSWFQNWEEMTNVSDSCSEMGLQ
GMRISKENLLQAKEVVNERETPFKVRIEEERQHEMVLEWKGISIVRVSTWM-

SlGRAS6 Solyc04g064550.1.1

Nucleotide sequence (ORF):
ATGTCTTTAGTTAGATCTCTAAGATCAATTGGAAATGGGAAGCTGTATTTTCAAAATGGTCACAATGATAA
CTCTAGTTTGGCTACATCCATGTACACCAAAAATGCACGCGGGATTATGTATGCAACCGAGTCTAGCAGCA
CAGACAGTTATGATCCAAAGTATCTGCTAGAATCACCTTCACCTTCTGAAGAACTTTTAAACACATCACCTA
CAGATGTCTTGGGGAATCCATTCCACCAGAGACATTCATCTTCTTTCCATCCATCAAGAGATTACAATCAG
GTTAGTTATGATTCAGCAGACTGTGTCAATCAAAGTCCAGATTCATCAGAATATAATGATGGAAGGGTGA
CAATGAAGCTTCAAGAACTTGAAAGGGTACTTTTCGATGATAACGAAATTGAAGGAGATGATGTATTTGC
TCGTGGCGAGACTGTGGACATAGATGATGAATGGTTTAACCAGATCCGAACAGAGTTACTTCAGGAGTCT
CCGAAGGAGTCTACATCGGCAGATTCTAATACCAGTAGCAGTAGTAGCTACAAGGAAATATCTGTATCTG
CTCCTCAAACTCCAAAGCAAATGCTGTTTAGTTGTGCTGCTGCTATTCAAGATGGACACATTGAACAAGCG
TCATCTATGATTAATGAACTGAGGCAGATGGTCTCCATCCAGGGAGATCCTCTTGGAAGGACTGCAGCTT
ACATGGTAGAAGCTCTTGCAGCTCGAATGGCTACATCTGGCAGAGGTCTTTATAAAGCTCTAAAATGCAA
AGAAGCCACCTCTTCTGAGAGACTCTCTGCTATGCAAGTCCTCTTTGAAGTATGTCCATACTTCAGGTTTGG
TTTTATGGCAGCAAATGGTGCGATCCTAGAGGCTTTTAAAGATGAGAAAAGAGTTCACATCATAGATTTTG

ACGTTAACCAAGGAAGTCAATATTACACTTTATTGCAGACATTGGGTAGTATGCCTGGTAAGCCACCCCAT
GTGAGGCTAACTGGGGTAGACGACCCTGAATCTGTCCAACGTGCTATTGGAGGCCTTAATGTCATTGGTC
TGAGGCTTGCACAGCTGGCCAAAGATCTTAAGATCTCTTTTGAGTTCCAAGCAGTCTCTTCCAACACTGCA
TTAGTCACCCCAGCAATGTTAAACTGCCGTCCTGGTGAAGCTGTTCTAGTAAACTTTGCGTTCCAGCTTCAT
CACATGCCTGATGAAAGTGTATCAACGGTAAACCAAAGAGACCAGCTTCTGCGGATGGTGAAGAGCTTAA
ATCCAAAGCTGGTAACAGTTGTTGAGCAAGACATGAACACTAACACTGCTCCATTTCTACAAAGGTTTGCA
GAAGTCTACAACTACTATTGTGCCGTATTTGAGTCTCTCGATGCAACTCTTTCAAGGGATAGCCAAGAGAG
GGTCAATGTAGAACGGCAATGTCTCGCACGTGATATCATCAACATTGTTGCCTGTGAAGGATTGGAGCGA
ATAGAGCGTTATGAGGTTGCAGGGAAGTGGAGAGCTAGGATGATGATGGCTGGTTTCACTCCATCCCCA
ATTAGCCGAAATGTCTATGAATCGATCCGGAATCTTATCAAGCAATATAGCGAAAGGTACAAAGCAGAGG
AGGAGGCAGGTGCACTTTATTTCGGGTGGGAAGACAAAACCTTGACTGTTGCTTCAGCTTGGAGGTAG

Amino acid sequence:
MSLVRSLRSIGNGKLYFQNGHNDNSSLATSMYTKNARGIMYATESSSTDSYDPKYLLESPSPSEELLNTSPTDVLG
NPFHQRHSSSFHPSRDYNQVSYDSADCVNQSPDSSEYNDGRVTMKLQELERVLFDDNEIEGDDVFARGETVDI
DDEWFNQIRTELLQESPKESTSADSNTSSSSSYKEISVSAPQTPKQMLFSCAAAIQDGHIEQASSMINELRQMVS
IQGDPLGRTAAYMVEALAARMATSGRGLYKALKCKEATSSERLSAMQVLFEVCPYFRFGFMAANGAILEAFKDE
KRVHIIDFDVNQGSQYYTLLQTLGSMPGKPPHVRLTGVDDPESVQRAIGGLNVIGLRLAQLAKDLKISFEFQAVS
SNTALVTPAMLNCRPGEAVLVNFAFQLHHMPDESVSTVNQRDQLLRMVKSLNPKLVTVVEQDMNTNTAPFL
QRFAEVYNYYCAVFESLDATLSRDSQERVNVERQCLARDIINIVACEGLERIERYEVAGKWRARMMMAGFTPSP
ISRNVYESIRNLIKQYSERYKAEEEAGALYFGWEDKTLTVASAWR-

SlGRAS7 Solyc07g065270.1.1

Nucleotide sequence (ORF):
ATGAGGCCTTCTCAGAACCCTGAGGAAATCAACGAGTTCAACAAATTCTATAACCAACCCATAGAGTATCA
AGAATCCTATTTCCTGCCTTCCGTTAACAATCCGAACAACAACCAATCGTTCTATGCTGATGTCTCTGCCTT
AAAACCCGCCCAACACTGTTATGTTGAATCATCCGCGGGAAATTCTAGTGAACTTGTTTCTGATTCACCTCC
TATTGTTAATTTCAACCCCATGATGAACCAAGGTTCTGATTCGTGTCGTTCAGACATGCATCATTCTCCTGA
TGATACATTTCAGTCGTCGGGTAACAGTTCGTGCTACACTAGTGATGTTACTGACCTGAAGCACAAGTTAA
GGGAACTTGAAACTGCAATGCTGGGGCCAGATTCAGAAAGCTTGGAATCATTTAATAATACTATCCCTGT
GACTGCAGCCAATCAAGTTCCATCAGAGTCGGATAAATTGGTTGGAATGATGGAGATGATGCCTAGCGG
AGACTTGAAAGAAGTTCTAATTGCTTGTGCTAAAGCAATAGCGGAGAATAATTTGATCACAGCTGAATGG
TTAATGTCAGAACTACGCACGGTGGTATCTGTTTGTGGGTCTCCCATACAACGTTTAGGTGCTTATATGCT
GGAAGGTTTAGTTGCTAGATTGGCCTCCTCGGGAAGCTCCATCTACAAGGCCTTAAGGTGCAAAGAGCCT
ACTAGTGTCGAGCTATTCTCGTACATGCACTTGCTTTATGAAATCTGTCCATACTTCAAGTTTGGATATTTG
TCAGCAAATGGTGCAATTGTTGATGCCATGAAAGATGAGAACTCAATTCATATAATCGATTTCCAGATTGC
TCAGGGTAGCCAGTGGATCACCTTAATCCATGCTCTTGCAGCACGGCCTGGTGGACCTCCAAGAATCCGC
ATCACAGGGATTGATGACTCTACATCAGCTTACGCCAGAGGAGGAGGGATTGAAATCGTAGGTCGAAGA
CTGTCAAGCATTGCTGCATCTTGCAATGTACCTTTCGAATTTCATCCCGTGTCTGCTTCTTGTCCAGATATTG
AGATTGAGCATCTTAAGGTTCTTCCCGGGGAACCATTGGCTGTTAACTTTGCGCTCGTCTTGCACCATATG
CCTGATGAGAGCGTTGGAACTCAAAATCACAGAGACAGACTTCTGAGGATGGTTAAAAGCTTATCTCCCA
AGATTGTTACCTTAGTGGAGCAAGAGTCCAACACTAACACAGCCCAATTCTTCCCCAGGTTCTTGGAGACA

CTGAACTACTACCTATCTGTTTTCGAATCAATAGACGTGGCTCTACCTAGAGACCATAAGGAACGAATCAA
CGTTGAGCAACACTGCTTAGCCCGTGAAATTGTCAACATATTAGCATGTGAAGGGGCAGAGAGAGTGGA
ACGACACGAGCTGCTTGAAAGGTGGAGATCACGATTTGCTGTGGCCGGGTTTAAACCATATCCGTTAAGC
TCCTCAGTAAATGCTACTATCAAGACCCTGCTGGAGAATTACTACCAAAGTTATACGCTTAATGAGAGAAA
TGGCGCGCTCTATCTTGGCTGGATGAATCGAGATTTAGTTGCTTCTTGTGCATGGAAATAA

Amino acid sequence:
MRPSQNPEEINEFNKFYNQPIEYQESYFLPSVNNPNNNQSFYADVSALKPAQHCYVESSAGNSSELVSDSPPIV
NFNPMMNQGSDSCRSDMHHSPDDTFQSSGNSSCYTSDVTDLKHKLRELETAMLGPDSESLESFNNTIPVTAA
NQVPSESDKLVGMMEMMPSGDLKEVLIACAKAIAENNLITAEWLMSELRTVVSVCGSPIQRLGAYMLEGLVAR
LASSGSSIYKALRCKEPTSVELFSYMHLLYEICPYFKFGYLSANGAIVDAMKDENSIHIIDFQIAQGSQWITLIHALA
ARPGGPPRIRITGIDDSTSAYARGGGIEIVGRRLSSIAASCNVPFEFHPVSASCPDIEIEHLKVLPGEPLAVNFALVL
HHMPDESVGTQNHRDRLLRMVKSLSPKIVTLVEQESNTNTAQFFPRFLETLNYYLSVFESIDVALPRDHKERINV
EQHCLAREIVNILACEGAERVERHELLERWRSRFAVAGFKPYPLSSSVNATIKTLLENYYQSYTLNERNGALYLGW
MNRDLVASCAWK-

SlGRAS8 Solyc02g085600.1.1

Nucleotide sequence (ORF):
ATGAAAGTGCCCTTTTCCACTAATGATAACGTAAGTTCTAAGCCATTGGTTAACAGCAACAACAGCTTTAC
TTTTCCAGCCGCTACCAACGGTTCTAATTTGTGCTACGAACCTAAGTCAGTGCTCGAGCTCCGTCGTAGCC
CAAGTCCTATAGTTGATAAGCAGATTATAACAACGAATCCTGATTTGTCTGCTCTTTGTGGTGGTGAAGAT
CCTCTTCAATTAGGAGATCATGTTCTGAGTAACTTTGAAGATTGGGATTCTTTGATGAGAGAACTTGGCTT
GCACGACGATTCTGCTTCGCTTTCAAAAACGAATCCTCTCACCCACAGCGAATCGCTGACTCAGTTCCATA
ATCTCTCCGAGTTTTCAGCTGAATCGAATCAGTTTCCTAGTCCTGATTTTTCTTTCTCAGACACTAATTTCCC
TCAGCAGTTTCCGACGGTGAATCAGGCAAGTTTCATCAACGCCCTTGATCTCTCCGGGGATATCCACCAGA
ATTGGAGCGTAGGATTTGATTATGTGGATGAACTCATTCGTTTTGCTGAGTGTTTCGAAACAAACGCTTTC
CAACTCGCACATGTGATACTGGCACGCCTCAATCAACGGCTCAGATCCGCAGCAGGAAAACCTCTCCAAC
GAGCTGCCTTTTACTTCAAGGAAGCACTTCAAGCGCAGCTCGCCGGATCAGCTCGGCAAACTCGTTCTTCA
AGTTCCTCCGACGTAATTCAAACGATTAAATCTTATAAAATATTGTCAAATATTTCTCCGATCCCTATGTTCT
CTAGCTTCACGGCGAATCAGGCCGTGCTTGAGGCAGTTGACGGCTCCATGCTAGTCCACGTCATCGATTTC
GACATCGGACTTGGTGGTCACTGGGCTTCCTTCATGAAAGAGTTAGCCGACAAAGCCGAGTGCCGTAAAG
CCAACGCGCCGATCCTTAGAATTACGGCTCTAGTTCCTGAAGAGTACGCTGTGGAATCAAGGTTAATTAG
AGAAAATTTAACCCAATTTGCTCGTGAACTCAATATCGGTTTCGAAATTGATTTTGTCCTAATTCGTACATT
CGAATTGTTATCCTTCAAAGCGATAAAGTTCATGGAGGGAGAGAAGACAGCGGTGCTTTTATCTCCCGCT
ATCTTCCGACGGGTCGGGTCAGGGTTTGTAAATGAACTCCGTCGAATATCCCCTAACGTGGTGGTACACG
TGGACAGCGAAGGACTTATGGGTTACGGTGCAATGTCTTTCCGGCAGACAGTAATCGACGGGCTGGAAT
TTTACTCTACACTACTGGAATCACTGGAGGCAGCAAATATCGGTGGTGGAAATTGCGGGGATTGGATGAG
AAAAATAGAGAATTTCGTGCTGTTTCCTAAGATAGTTGATATGATCGGAGCAGTAGGGCGGCGTGGAGG
TGGAGGATCGTGGAGGGATGCCATGGTGGATGCCGGATTCCGGCCGGTTGGACTAAGCCAGTTTGCCGA
TTTTCAAGCAGATTGTTTATTGGGTAGGGTACAGGTAAGAGGATTCCACGTGGCAAAGAGACAAGCAGA
GATGTTGCTTTGCTGGCATGACAGGGCCCTAGTAGCCACGTCAGCTTGGAGGTGTTAA

Amino acid sequence:

MKVPFSTNDNVSSKPLVNSNNSFTFPAATNGSNLCYEPKSVLELRRSPSPIVDKQIITTNPDLSALCGGEDPLQLG
DHVLSNFEDWDSLMRELGLHDDSASLSKTNPLTHSESLTQFHNLSEFSAESNQFPSPDFSFSDTNFPQQFPTVN
QASFINALDLSGDIHQNWSVGFDYVDELIRFAECFETNAFQLAHVILARLNQRLRSAAGKPLQRAAFYFKEALQ
AQLAGSARQTRSSSSSDVIQTIKSYKILSNISPIPMFSSFTANQAVLEAVDGSMLVHVIDFDIGLGGHWASFMKEL
ADKAECRKANAPILRITALVPEEYAVESRLIRENLTQFARELNIGFEIDFVLIRTFELLSFKAIKFMEGEKTAVLLSPAIF
RRVGSGFVNELRRISPNVVVHVDSEGLMGYGAMSFRQTVIDGLEFYSTLLESLEAANIGGGNCGDWMRKIENF
VLFPKIVDMIGAVGRRGGGGSWRDAMVDAGFRPVGLSQFADFQADCLLGRVQVRGFHVAKRQAEMLLCW
HDRALVATSAWRC-

SlGRAS9 Solyc06g036170.1.1

Nucleotide sequence (ORF):

ATGTCCTCGGATTTCTCCGGCGGAGTTCCAGACTTTCACGGCGGCGCCGGTAGATCCAGCTTGATTCCGAT
GAACAACTCCCAGCCACAAATTCAATTAACCCAACGCCCTGACGGAGTTTCTCAGAATCTCCACCGGAGAC
CTATGTTCATCGGGAAACGGTCACTCGCAGCCTTTCAACAGCAGCAACAGTTTCAGTTTCTACAACAACAA
CAGCAGCAACAAGGTTTGGGATTTTATCTACGTAACGTGAAGCCTAGAAATTACCAGCAGGCATCTCCAA
TTTCTCCTCTAGATTACTCTGTTTCTTCCTCGTTGATTTCATCTGAATTTTCTCCGATGACTCCACGCCATCCT
CTCCCGATTTCCACGGCGAACACGAACGGGGTTTTATCTTCTGGTAACCCGAATTGTTCAGCAGTTGCTTC
TTACCTAAATCAGGTACAGAACAGTTTATACCAGGAATCAGAGGAAAAAATGATGAATCGACTGCATGAG
TTAGAGAAACAGCTCCTAGAAGACAACAATGAGGAGGAAGAAGATACAGTCTCTGTTGTGACTAACAAC
GACGAGTGGTCGGAAACAATAAAGAATCTGATTACTCCGACTAGTAACCACTTATCCCCGGCATCATCTAC
GTCTTCATGTTCTTCTTCTATGGAATCTCCGCCAGTATCTTCTCCCAGGCAGTCTATTGTCGAAGCTGCTACC
GCAATAATCGACGGAAAAACCAATGTTGCAGTACAGATCCTCACGCGCCTCGCACAGGTAGCTGATGTTA
GAGGGTCTTCCGAACAGCGGCTGACGGCGTACATGGTTTCAGCACTCCGATCGCGCGTGAACTCCACGGA
GTACCCACCGCCCGTGATGGAGCTGCGTAGCAAAGAGCACGCAGTTTCAGCTCAAAACCTCTACGAGATA
TCCCCGTGTTTCAAGCTAGGATTTATGGCAGCTAATTTCGCCATTGTTGAAGCTGTAGCTGATCATCCCTCA
AACAAAATTCACGTCATTGATTTCGACATAGGACAAGGTGGACAATACTTGCATTTACTACACGCGCTAGC
TTCCAAGAAAACAGATTATCCCATCAGCTTAAGAATCACGGCGATCACAACAGAGTTCACGGTCAGAGCT
GATCACAGTTTAAAATCCATTGAAGATGATCTGAGAAGTCTAGCAAACAAAATCGGTATTTCCTTGATTTT
CAAAGTAATTTCACGTACAATCACCGATTTGAGTAGGGGAAAATTAGGGATTGAACACGACGAAGCTTTA
GCAGTGAATTTCGCATACAGATTATATAGATTACCCGACGAGAGCGTAACAACAGAGAATCTAAGAGACG
AGCTTCTCCGGCGAGTGAAGGGGTTATCACCAAAGGTGGTGACATTAGTAGAGCAAGAGTTGAACGGGA
ACACGGCGGCGTTTGTGGCGCGTGTAAACGAGGCGTGTGGATATTACGGAGCATTGTTGGATTCACTGG
ATGCAACTGTATCAAGAGAGGAAACGGGTCGGGTCAAGATCGAAGAAGGGCTGAGTCGTAAATTAACAA
ACTCGGTAGCGTGTGAAGGTAGGGATCGTTTGGAGAGATGCGAGGTGTTTGGTAAATGGAGGGCCCGA
ATGAGTATGGCTGGGTTCGGGCCGAGGCCCATGAGTCAACAAATTGCTGATTCACTGCTTAAGAGGCTTA
ACTCGGGCCCACGTGGCAATCCAGGATTCAATGTGAATGAACAAAGTGGGGGTATTAGGTTTGGATGGA
TGGGAAAAACCCTCACCGTTGCTTCTGCTTGGTGTTAA

Amino acid sequence:

MSSDFSGGVPDFHGGAGRSSLIPMNNSQPQIQLTQRPDGVSQNLHRRPMFIGKRSLAAFQQQQQFQFLQQ
QQQQQGLGFYLRNVKPRNYQQASPISPLDYSVSSSLISSEFSPMTPRHPLPISTANTNGVLSSGNPNCSAVASYL

NQVQNSLYQESEEKMMNRLHELEKQLLEDNNEEEEDTVSVVTNNDEWSETIKNLITPTSNHLSPASSTSSCSSS
MESPPVSSPRQSIVEAATAIIDGKTNVAVQILTRLAQVADVRGSSEQRLTAYMVSALRSRVNSTEYPPPVMELRSK
EHAVSAQNLYEISPCFKLGFMAANFAIVEAVADHPSNKIHVIDFDIGQGGQYLHLLHALASKKTDYPISLRITAITT
EFTVRADHSLKSIEDDLRSLANKIGISLIFKVISRTITDLSRGKLGIEHDEALAVNFAYRLYRLPDESVTTENLRDELLR
RVKGLSPKVVTLVEQELNGNTAAFVARVNEACGYYGALLDSLDATVSREETGRVKIEEGLSRKLTNSVACEGRDR
LERCEVFGKWRARMSMAGFGPRPMSQQIADSLLKRLNSGPRGNPGFNVNEQSGGIRFGWMGKTLTVASAW
C-

SlGRAS10 Solyc03g025170.1.1

Nucleotide sequence (ORF):
ATGTCGTCAGGTTTCTCCGGCGAGATTTACGGCGCCGGTGGGAGATCTACCATGAATAACAACAACTCAC
AAAGGCCTTATTCGTCGCCGCTATCGGGTATTTTGCCTGACCCGGTTACGCAGATGGTTCATCAAGGGAG
ACCTAATTTGATGGGGAAGCGATCTCTAGCGGAGTTTCAACAACAGCAGCAATTGCAGTTTCTACAACTTC
AACAGCAGAAACAACAGCAGCAGCAGATGATGCTACTTCAACAGCAGCAGCAGCAAGGAATTGGGTTTT
ATCTCCGTAACGTAAAGCCTAGAAGTTATCAGCAGTCTTCCCCAATGTCTCCTCTTTCCCCTGTAGATTTTTC
GATTGCCGCTGCTGCTTCAATTTCATCTTCGAATTCAAATGTATCCACCATGATGAACACGCGCCAGGCTCT
ACCGGTTCTGCAGCAGCCTGCTAATATGGGTGGGTTTTTATCTCCGGGTATTCAGAATTATTCTACAGGGG
TTTCGTCTCTAAATCCGGTTCAAAACGGAGCAACCATCGGAGTACAGGAATCTGATAAAAAAATGATGAA
TTGTCTTCAAGAGCTTGAGAAACAGCTCTTAGACGACAACGATGAAGAAGAAGGCGATACAGTTTCGGTC
ATCACCAACAACGAGTGGTCGGAGACGATACAGAATCTGATTAGCCCCTCTCAGAACCAAAACCAAATTC
AGAAACTGGCATCACTATCTCCATCCTCTTCGACTTCTTCGTGTGCTTCTTCAACAGAATCTCCGGCAATAT
CTTGTCCCAAACAGTCTATAATAGAAGCAGCCACAGCAATATACGATGGAAAAAACGACGTCGCACTGGA
AATCCTCACGCGCCTATCACAAGTTGCCAATATCAGAGGCTCCTCCGACCAGCGGCTAACGGCATACATG
GTCGCCGCTTTACGGTCGCGCTTGAACCCCGTCGATTACCCACCGCCGGTCCTGGAGCTGCAAAGCAAAG
AACATACAGAGACTACTCACAATCTATACGAGGTATCTCCCTGTTTCAAGCTCGGTTTCATGGCTGCCAAT
CTAGCCATCCTTGAAGCTGTAGCTGACCACCCATTTAACAAGCTTCACGTCATTGATTTCGACATCGGCCA
AGGCGGACAGTACTTGCATTTACTGCATGCACTGGCTGCCAAGAAATCAAACAACCCCGCCGTTTTAAAA
ATCACTGCTTTCACAGAACAAGCCGGCGGAGTTGACGAGAGACTCAATTCCATCCACATGGAGCTTAACT
CCGTCGCTAATCGGCTCGGGGTCTGCTTATATTTCAATGTAATGTCCTGTAAAGTAGCCGATTTGAGCCGA
GAAAACTTAGGCCTCGACCCTGATGATGCATTAGCAGTTAATTTCGCATTCAAATTATACAGATTACCCGA
CGAAAGCGTCACAACAGAGAATCTCCGCGACGAGCTTCTCCGTCGGGTAAAAGCGTTATCACCGAAGGTG
GTAACGGTGGTGGAGCAAGATATGAACGGCAACACGGCGCCGTTTCTAGCGCGTGTGAACGAAGCGTGT
GGGCATTACGGTGCGATATTCGACTCGCTGGATGCTACAGTTCCACGAGACAGCATGGACCGAGTCAGG
ATCGAAGAGGGACTGAGTCGCAAAATGTGTAATTCGGTAGCGTGCGAAGGGAGGGACCGCGTTGAAAG
ATGCGAGGTATTTGGGAAGTGGAGGGCCCGTATGAGCATGGCTGGGTTCGGGCCGAAGCCCGTTAGTCA
AATCGTGGCGAATTCTTTGCGTTCGAAGCTTAATTCGGGTACACGTGGCAACCCGGGCTTCACCGTGAAC
GAACAAAGTGGAGGTATTTGCTTTGGTTGGATGGGACGGACTCTCACCGTCGCGTCAGCTTGGCGTTAA

Amino acid sequence:
MSSGFSGEIYGAGGRSTMNNNNSQRPYSSPLSGILPDPVTQMVHQGRPNLMGKRSLAEFQQQQQLQFLQL
QQQKQQQQQMMLLQQQQQQGIGFYLRNVKPRSYQQSSPMSPLSPVDFSIAAAASISSSNSNVSTMMNTR
QALPVLQQPANMGGFLSPGIQNYSTGVSSLNPVQNGATIGVQESDKKMMNCLQELEKQLLDDNDEEEGDTV

SVITNNEWSETIQNLISPSQNQNQIQKLASLSPSSSTSSCASSTESPAISCPKQSIIEAATAIYDGKNDVALEILTRLS
QVANIRGSSDQRLTAYMVAALRSRLNPVDYPPPVLELQSKEHTETTHNLYEVSPCFKLGFMAANLAILEAVADHP
FNKLHVIDFDIGQGGQYLHLLHALAAKKSNNPAVLKITAFTEQAGGVDERLNSIHMELNSVANRLGVCLYFNVM
SCKVADLSRENLGLDPDDALAVNFAFKLYRLPDESVTTENLRDELLRRVKALSPKVVTVVEQDMNGNTAPFLAR
VNEACGHYGAIFDSLDATVPRDSMDRVRIEEGLSRKMCNSVACEGRDRVERCEVFGKWRARMSMAGFGPKP
VSQIVANSLRSKLNSGTRGNPGFTVNEQSGGICFGWMGRTLTVASAWR-

SlGRAS11 Solyc12g099900.1.1

Nucleotide sequence (ORF):
ATGTTACAAGATGATGGTTCTTCATCTGTGACCTCATCATCACCTATTCAAGTATTTCCAATGATGTCTGTA
TCACCTAGCTTTGGTTCATCAAATCAGTGGCTTAAGGAGCTGAAATCTGAAGAAAGAGGGTTGTATTTGA
TACATCTTTTGCTTGCTTGTGCTAATCATGTTGCTTCTGGTAGCCTTGAGAATGCTAATATAGCACTTGACC
ATATTTCCCAACTTGCATCTCCTAGTGGAGATACCATGCAAAGGATTGCTTCATATTTTACTGAGGCTTTAG
CTGATAGGATTCTAAGGAGTTGGCCTGGTCTTTATAAGGCGTTGCGTTCGACTAAGTTATCGGTTGTCTCA
GAAGAAATTCTTGTTAGGAAGATGTTTTTCGAGATCTTTCCTTTCTTGAAGGTGGCGTTTGTGGTCACAAA
TCAAGCTATAATTGAAGCTATGGAAGGTGAAAAGATGGTTCATATTGTGGATCTTAATGCTGCTGAACCC
CTGCAATGGCGTGCGTTGCTTCAGGACTTGAGCGCGCGTCCTGAAGGACCGCCCCATCTGCGCATTACTG
GGGTTCATCAGCAAAAAGAGGTGTTAGATCAAATGGCACATGTGCTTACTCAAGAAGCAGAAAAACTGG
ATATCCCTTTTCAGTTCAATCAAGTAGTTAGCAGATTGGAAAATCTTGATGTTGAGAAACTTCGCGTGAAA
ACGGGGGAGGCTCTTGCGATTAGTTCAATTATGCAATTGCACACCCTTCTAGCCCATGATAATGACAAGAA
GTCCCCTTTGCCTTTTAAGCATTCAAATGGTGTTAACTTAAACAGGGCACTAGTCAACCAAAATACTTTAGG
GGAATTTCTTGAAAAAGATATGGCTAATGGTTGCAGTCCAAGCAATGACACCGCTTCTTCATCCCCGCTAT
GTTCAACTGGTTCAACAAAGATGGATAGTTTCCTCAATGCTTTGTGGGGTTTATCACCAAAAGTTATGGTG
GTAACAGAACAAGATGCTAACCATAATGGGACAACTCTTATGGAGAGGCTATCAGAGTCGTTACATTTTT
ATGCTGCATTATTTGATTGTCTTGAATCGACGCTGCCAAGAACATCATTAGAGAGATTAAAGGTGGAAAA
GATGTTATTAGGTGAAGAGATTAGAAACATTATAGCATGTGAAGGGATCGAACGAAAGGAGAGGCATGA
AAAGCTCGAAAAGTGGTTCCAAAGATTCGACACATCTGGTTTTGGGAATGTGCCTTTGAGTTATTATGCTA
TGTTGCAGGCAAGAAGGTTGTTGCAGAGTTACAGTTGTGAAGGATACAAGATCAAAGAAGATAATGGTT
GCGTGGTGATATGCTGGCAGGATCGCCCACTTTTCTCAGTGTCGTCTTGGCGATGTAGGAAGTGA

Amino acid sequence:
MLQDDGSSSVTSSSPIQVFPMMSVSPSFGSSNQWLKELKSEERGLYLIHLLLACANHVASGSLENANIALDHISQ
LASPSGDTMQRIASYFTEALADRILRSWPGLYKALRSTKLSVVSEEILVRKMFFEIFPFLKVAFVVTNQAIIEAMEG
EKMVHIVDLNAAEPLQWRALLQDLSARPEGPPHLRITGVHQQKEVLDQMAHVLTQEAEKLDIPFQFNQVVSR
LENLDVEKLRVKTGEALAISSIMQLHTLLAHDNDKKSPLPFKHSNGVNLNRALVNQNTLGEFLEKDMANGCSPS
NDTASSSPLCSTGSTKMDSFLNALWGLSPKVMVVTEQDANHNGTTLMERLSESLHFYAALFDCLESTLPRTSLE
RLKVEKMLLGEEIRNIIACEGIERKERHEKLEKWFQRFDTSGFGNVPLSYYAMLQARRLLQSYSCEGYKIKEDNGC
VVICWQDRPLFSVSSWRCRK-

SlGRAS12 Solyc07g047950.1.1

Nucleotide sequence (ORF):

ATGCAAGCATCACGGCGCAGAAGAACAACCATGTCTAATATGCTTTACTGTGAACCTGTGCAGAAGGCAG
AGGCCTACTATCTGCCTCAGTTTCAAACTTTAGAAAGCCATTTGAGCTGCATTAATGGCAGCCTTGGAGGT
AATCACTCCTTCCAGACTTATCATGACCGCTATTGCATGCTGGAGGCGTCTTCTGCCACTGGAAGCAACGC
TGTTTACCACTCACCATCAACTGCTAGTTTCTCATCAAATGGAAGCACGACGTGCAATCAAGAATCTCAGC
TGTACCTGTCAGATGTTCGTCAGTCCCCTGAGACAACTAACTATGGCTCACCCATAAGTGGATCTTGTATT
ACAGATGATGTGACTGATTTCATGCACAAGCTAAAGGAATTGGAAACAGCTATGCTGGGACCTGATGCGG
ACTTCCAGGAGAGTTATGATAATTCCTTGGCAAGCAGTATAGCTTCCTCAGAAATTGATAGCTGGAGGCA
AATGATGTTGGCCATACCTAGACGGGATTTGAAACAGGTGCTTATTGCTTGCGCAAAAGCAGTTTCTGAT
GGTGAATTAGTAACTGCACAAGTGTTGATATCTGAGTTGCGTCAAATGGTATCAGTGTCAGGGGAACCAA
TTCAGAGACTGGGAGCATACATCTTGGAAGGGCTTGTTGCAAGGTTGGGTGCATCAGGAAGTTCCATATG
CAAATCCTTGAGATGCAAAGAACCCGCAAGTTTTGAGCTGTTGTCTTATATGCACGTTCTTTACGAGATTT
GCCCTTACTTCAAATTTGGATACATGTCAGCTAATGGTGCCATTGCAGAAGCAATGAAGGACGAAAATAG
AGTCCATATAATTGATTTTCAAATTGCTCAAGGGAGCCAGTGGGTGCCTATGATCCAAGCTTTTGCAGCTC
GTCCTGGAGGACCCCCACATATCCGCATAACAGGCATTGATGATTCCACCTCAGCATATGCTCGTGGAGG
AGGCCTTGATATCGTGGGGCAGAGGCTTTCCAAACTTGCTAAAACTTTCAAGGTTCCTTTTGAGTTTCATC
CTGCTGCCATGTCGGGTTCTGATATTCAGCTAAAAAACCTTGGAATTCAACCTGGAGAAGCACTAGCAGTA
AATTTTGCTTTCACATTACATCACATGCCAGATGAAAGTGTGAGCACTGAAAATCATCGGGATAGACTATT
ACGGATGGTCAAAAACCTCAACCCCAAGGTGGTTACCCTTGTTGAGCAAGAATCTAATACAAATACTGCT
GCTTTCTTTCCTCGATTTCTTGAAACCTTGGATTATTATTCAGCCATGTTTGAATCAATCGATATGACTCTTC
CAAGGGGACACAAGGAGCGCATCAATGTTGAGCAGCATTGTTTGGCGAGGGACGTTGTTAACATCATAG
CATGTGAAGGGATTGAAAGGGTAGAACGACATGAACTTCTTGGTAAGTGGAAGTCGCGGTTTAGAATGG
CTGGTTTTAACCCATACCCATTGAGTTCATTGGTGAACGCTACGATCAAGACATTGCTAGAGAGCTACTCT
GATAAGTATAGGCTTGAAGAAAGAGATGGTGCTCTTTACCTTGGTTGGATGAACAGAGATTTGGTTGCTT
CTTGTGCCTGGAAATAA

Amino acid sequence:

MQASRRRRTTMSNMLYCEPVQKAEAYYLPQFQTLESHLSCINGSLGGNHSFQTYHDRYCMLEASSATGSNAVY
HSPSTASFSSNGSTTCNQESQLYLSDVRQSPETTNYGSPISGSCITDDVTDFMHKLKELETAMLGPDADFQESYD
NSLASSIASSEIDSWRQMMLAIPRRDLKQVLIACAKAVSDGELVTAQVLISELRQMVSVSGEPIQRLGAYILEGLV
ARLGASGSSICKSLRCKEPASFELLSYMHVLYEICPYFKFGYMSANGAIAEAMKDENRVHIIDFQIAQGSQWVP
MIQAFAARPGGPPHIRITGIDDSTSAYARGGGLDIVGQRLSKLAKTFKVPFEFHPAAMSGSDIQLKNLGIQPGEA
LAVNFAFTLHHMPDESVSTENHRDRLLRMVKNLNPKVVTLVEQESNTNTAAFFPRFLETLDYYSAMFESIDMTL
PRGHKERINVEQHCLARDVVNIIACEGIERVERHELLGKWKSRFRMAGFNPYPLSSLVNATIKTLLESYSDKYRLE
ERDGALYLGWMNRDLVASCAWK-

SlGRAS13 Solyc06g076280.1.1

Nucleotide sequence (ORF):

ATGGATCCTCGATTCATTCCGTTGTCTGATCCTGTAAACACTTTCGAGTTCGAAGATCAGATTAATTTGTCT
AGCTATGAGGGATCTCTGAATCCTCCTCACAATTATAATGATGACTATGTTGCTTTTGGTGTTCCGTATACG
GCCCCAAGTGTAGACATTGGTAATTTCGCCCCATCATCAAACGTGAGCTCGGAAGTGGACTCTCCAGATG
ATCATGACTCTGATTTTCTGTTTAAGTACTTAAACCAGATACTTATGGAGGAGAATATTGAAGATAAGCCC

AGCATGTTTCATGACCCGCTTGCTCTAAAAGCTGCTGAGAAGTCCTTATATGAAGCCCTTGGCAAGTCGTA
CCCTCCTTCACCCTATCATGTTGACCATCAGTTAGAAAGCCCAAGCCCTGACAGCATTTTCCAGACTTCCAG
TGACCATAGTACAAGTAGTAGCAATGCTCATAGTAATTCCATGGATCCTCACTGGATTGTTGATCCTGGAG
AATCTAGTTTGTCTTTGCCTGTGGAGAGTCATCCATCAGAGTACTCCATCCAACCTTTGATGCAGAGTAACT
CAGAGAGGTCTCATGGTTCTTTAAACAACATCAACAACTTGAATGTCCATATGGACTCTTTTTTAAATCCTA
ATGCTCTTTCAAACATGTTTACTGATCGCGAGTCTATCTTACAGTTCAAGAGAGGGGTGGAGGAAGCTAA
TAAATTCCTTCCAAATGTTAGTCAATTTGTTGTTGATTTGGATAAATATACCTTTCCTCCAAAGGTGGAAGA
AGTGACCAAAGAGGCTGTGGTCAAGGTAGAGAAGGATGAGAGGAATCACTCACCTAATGGGACTAAAG
GAAGAAAACATCAGTATCCTGAGGACAGTGATTTTGAAGATGAAAGGAGCAACAAACAATCAGCAATAT
ATGTGGAGGAGGAGGCTGAGTTATCAGAGATGTTTGACAGGGTTCTGCTCTGTACAGATAAAGGTGAGA
CAATATGTGGTGATGTCAAGTGTGAAATGCCAGTAGACAATAGTTTAGACCAGAATGGACAAGCACATG
GATCAAATGGTGGAAACACTCGTGCAAAGAAACAAGGGACTAAAAATGAAGCTGTGGATCTAAGGACTC
TCTTAGTTAGCTGTGCACAGTCTGTTGCTGCTGACGATCGCAGGACAGCATATGAACAGCTAAAGCAGAT
CAGGCAGCATTGTTCTTCCATTGGTGATGCATACCAAAGGCTGGCCAGTGTATTTGCGGATGGCCTTGAA
GCCCGGTTGGCTGGCACTGGCACTCAAATATATGCTGCCCTTGCTCCGAAAAAGATTACAGCTGCTGAGA
AATTGAAAGCATACCAGGTTTATCTTTCGGCATGCCCGTTCAAGAAAATATCAATATTCTTTGCGAATAAA
ATGATCTTCCACACAGCATCTAATGCCAGGACATTGCATCTCATAGATTTTGGTATACTATATGGTTTCCAG
TGGCCAATACTCATCCAGCTTCTCTCAGAGATACCTGATGGGCCTCCAAAGCTTCGCATTACTGGAATAGA
CCTTCCCCAACCTGGATTCAGGCCAGCAGAAAGCTTAGAACAGACAGGGAGCCGATTGGCAAAATATTGT
GAGCGCTTTAAAGTACCATTTGAATATAATGCCATAGCAACACAGAATTGGGAGAACATCAAACTTGAGG
ACTTGAAACTTGCAAGTGGTGAGACTGTTGCTGTGAACTGTCTTTTTCGTTTTAAGAACCTGTTGGATGAG
ACAGTGATGTTGGACAGTCCAAGGGATGCAGTTCTGGGCTTAATTAGAAAGATGAATCCGGATATCTTCG
TGCAAGCTGTAATCAATGGATCTTACAGTGCACCCTTCTTTGTCACTCGCTTCAGGGAGGCCCTCTTCCATT
ACTCAACTCTCTTTGACATGTTTGATGCTACCTTACCCCGTGGTGATCAGAAGAGGTTGCATTTTGAACAA
GAATTTTACAGACGCGAGGCAATGAATGTCATTGCTTGTGAGGGTTCCGAGAGGGTTGAGAGGCCTGAG
ACGTACAAGCAATGGCAGGTTCGCAATATGAGGGCTGGATTCAAGATTCTTCCGTTGAACCAACAACTCG
TGCAGAAGTTAAGGTGCAAGGTAAAGGCTGGATACCTCCGCGATTTTGTGTTTGATGAGGATGGTAAATG
GATGTTACAGGGTTGGAAAGGCCGGGTAGTATGCGCTAGCTCGTGTTGGGTGCCAGCATAG

Amino acid sequence:
MDPRFIPLSDPVNTFEFEDQINLSSYEGSLNPPHNYNDDYVAFGVPYTAPSVDIGNFAPSSNVSSEVDSPDDHD
SDFLFKYLNQILMEENIEDKPSMFHDPLALKAAEKSLYEALGKSYPPSPYHVDHQLESPSPDSIFQTSSDHSTSSS
NAHSNSMDPHWIVDPGESSLSLPVESHPSEYSIQPLMQSNSERSHGSLNNINNLNVHMDSFLNPNALSNMFT
DRESILQFKRGVEEANKFLPNVSQFVVDLDKYTFPPKVEEVTKEAVVKVEKDERNHSPNGTKGRKHQYPEDSDF
EDERSNKQSAIYVEEEAELSEMFDRVLLCTDKGETICGDVKCEMPVDNSLDQNGQAHGSNGGNTRAKKQGTK
NEAVDLRTLLVSCAQSVAADDRRTAYEQLKQIRQHCSSIGDAYQRLASVFADGLEARLAGTGTQIYAALAPKKITA
AEKLKAYQVYLSACPFKKISIFFANKMIFHTASNARTLHLIDFGILYGFQWPILIQLLSEIPDGPPKLRITGIDLPQPG
FRPAESLEQTGSRLAKYCERFKVPFEYNAIATQNWENIKLEDLKLASGETVAVNCLFRFKNLLDETVMLDSPRDA
VLGLIRKMNPDIFVQAVINGSYSAPFFVTRFREALFHYSTLFDMFDATLPRGDQKRLHFEQEFYRREAMNVIACE
GSERVERPETYKQWQVRNMRAGFKILPLNQQLVQKLRCKVKAGYLRDFVFDEDGKWMLQGWKGRVVCASS
CWVPA-

SlGRAS14 Solyc04g014830.1.1

Nucleotide sequence (ORF):

ATGCAAGCATCTCAGGGACCTCAAAGGTCGTGTAGTGTACATAAATTGTACAATAAGCCAATGCAGCAAG
TTCAGCAAAACTACACCCCTTGCCGTGCTTCTGACAACAGCAATTATAATGATGGTAGCAACTCCCAGGCA
CAGGTTTCTTTAACGACAGAGAATGAGAAGTTCTTCACTGTGGACACATTTCCGGCTACTGACTGTGCCAT
CTACGATGGAGATCCTTCCGTAAGCGTCTCTTCCAACAGGAGTCCTTTCTCTTCTCAATGTTCTCAGTCGAA
CATGTTTGAGCAACGTCGTTCTTACGAGAAAACTGCTGGTTCACCTGTAAGTTTGTGTTCAGGAGTTGATG
ACAGCAACGGGAAGAAGCATGAGCTGCGGGAACTGAATAATAAGTTGCTAAGGCCTGAATCCGATATTG
ATGACAGCTGCAGTTGCTCATTAAATGGTGTAGTCTCAAAACATTTTTCCTTGACAAGGCGGAATCAAGTA
TTGGACGTAGCCTCAAGATTGGACTTGAAAGAGTTGCTTGTTGCCTGTGCTGAAGCAGTAGATGAAGCTG
ATACCTCGACTGCAGAAGTTCTGATGGATGCTTTGGAGAAAAGGGTATCGGTTTATGGGGAACCTATGCA
ACGACTGAGTGCATACATGTTGGAAGGTCTAAGAGCACGATTATTGTCCTCCGGGAGTAACATATACAAA
AAACTGAAGTGCAATGAACCAACTAGCTCAGAATTGTTGTCCTACATGCAAGTCCTGTATCACATCACCCC
TTACTTCAAGTTCGCTTATATGTCCGCCAATGTTGTCATAAGCGAAGCCATGAAGAATGAGAATAGAATCC
ATATCATTGATTTTCAAATTGCACAGGGAAGTCAATGGGTGTTCCTCATCCACTATCTGGCTCGTCGGCCT
GGTGGTCCGCCATTTCTTCGCATCACAGGCATTGATGATTCCCAATCAGCTCATGCACGCGGTGGTGGACT
TCAGCTAGTTGGCGAAAGGTTAGCGAGCATTGCCAAGTCCTGCGGAGTGCCTTTCGAATTCCATACTGCT
GCATTGTCAGGCTGTATGGTCAAACTAGAGAACCTAAGAGTTAGACATGGAGAAAGCCTGGCAGTTAACT
TCCCTTACATGTTGCACCACATGCCAGACGAGAGCGTAAGCACTATGAACCATCGGGACCGCCTATTAAG
ACTAGTCAAGAGCTTGTCTCCCAAAATCGTGGCCTTAGTTGAACAAGAAATGAACACCAATACCGCCCCTT
TCCTTCCAAGGTTCCGTGAAACTCTAGATTACCACAAAGCAATATTCGAATCAGTCGATGTAACTCGCCCG
AGGAATGACATGCAACGGATCAGATCAGAGGAGCATTGTATTGCACGGGATGTTGTCAATCTCATAGCAT
GTGAAGGGGCTGATAGAGTGGAAAGGCATGAAGTTTTTGGCAAGTGGAGGTCAAGACTTTTGATGGCTG
GATTTACTCCATGCCCGCTGAGTCCATCGGTTGCTGAGGCCATTAAGGTCATGTTGAAGGAGTATAGCTCA
AATTATAAGCTTGCTGAAAGCCAGGGGGCGCTTTATATTGGATGGAACAACAGAGCTTTAGCAACTTCTT
CTGCTTGGCAATTACCTCATTCGCTGCCTTTGGGATCTTGA

Amino acid sequence:

MQASQGPQRSCSVHKLYNKPMQQVQQNYTPCRASDNSNYNDGSNSQAQVSLTTENEKFFTVDTFPATDCAI
YDGDPSVSVSSNRSPFSSQCSQSNMFEQRRSYEKTAGSPVSLCSGVDDSNGKKHELRELNNKLLRPESDIDDSC
SCSLNGVVSKHFSLTRRNQVLDVASRLDLKELLVACAEAVDEADTSTAEVLMDALEKRVSVYGEPMQRLSAYML
EGLRARLLSSGSNIYKKLKCNEPTSSELLSYMQVLYHITPYFKFAYMSANVVISEAMKNENRIHIIDFQIAQGSQW
VFLIHYLARRPGGPPFLRITGIDDSQSAHARGGGLQLVGERLASIAKSCGVPFEFHTAALSGCMVKLENLRVRHG
ESLAVNFPYMLHHMPDESVSTMNHRDRLLRLVKSLSPKIVALVEQEMNTNTAPFLPRFRETLDYHKAIFESVDV
TRPRNDMQRIRSEEHCIARDVVNLIACEGADRVERHEVFGKWRSRLLMAGFTPCPLSPSVAEAIKVMLKEYSSN
YKLAESQGALYIGWNNRALATSSAWQLPHSLPLGS-

SlGRAS15 Solyc10g074680.1.1

Nucleotide sequence (ORF):

ATGGCCAAGGCTTTACCTGTGAGTGGTGAGAACTGCACCGGTGTGAACGTCGTCGGTGATAGTAGTGGT
GAGAAGACAAGCAAGCAACTTTTCCACTGTGAAAACGGAAAAATGGTTAGGAAGAGAGCGGCGTCTGAC
ATGGAGATCCAGACCGGCGCCGGTGAAGAGCATAGATATTTACGCCGGCCTGCTATGATAGGGGGGTCT

CATTCTCAGGTTGGTGATTCAAGGGTTTGTGGTAATAGTAATTTTGGTCATGGTATGAACAGTAATTTGAC
AACGACGATGACGATGACGACTCAGGTTTCTAACTACTCCACTATGCAAATGTTACCTTCTTCCACGAACTT
GTGCGGTGTGACGTCAAGAGGAGGGCCTGGGATTGATACCGGATTTTCTAATTCAACCCCAAACCTAACT
TACACAGACGCCATAACATCGCATCATCAGCCACAAGGTACTCAAACCCAGAACAACAACAGTCAATCCCC
ATCTGTTTGTGTTTTCTCCGGTCTGCCGCTTTTTCCTCCAGATAGAAACCGCCAAAACAGTGGTTTACTACT
GCAACAACCTCCTGCTGCTGCAGCAGCTGTTGTATCTTCACCTCTTACAACTGGAAGAATCGATTCCATGG
AGGATAGTACGTCGGCAACGGCATGGATTGACAGTATTATAAAGGATTTAATCAACAGCTCCGCACAAGT
ATCGGTGCCCCAACTCATACAGAATGTAAGGGAGATTATTCACCCTTGCAATCCGTATCTCGCATCTCTTCT
CGAGTACAGGCTTCGCTCTCTCACTAGTAATAATAATGGCGGTGCTGATCAAAATGACCCCATGGAGTGC
TGGAGAAGAAAAGAATCGCTTCCGGCACAGCTCGCTGGTTTACAACAGGCCCAAAATAATGCCAATTTAT
TGCAGCACAATATTTTATCCCTTCCTGATTCCTCAAATAATCAATACTTAAACTGGGATATAGCTTTGCCAA
ACAGCCATAACGCTCCGGTAGCGCCTTCTCATAACCAGCACCAGCAGCTAGGCGGCAATAATCCTACTGC
AACAGATCTTTCATTTGTTACACTTTCTCCTCAAGTGCAGCAGCAGCAACAACAACAACAACAAGAATCTC
CCCATTCTCATTCTCAGCAACAGGCCGCAGTAGACTTAGATCAACAACAGAAGCAGCAGCAATCTTCTAGT
TCTCTATCTCCAACATCGGTAGCCGACAATAGCGCCAAAACAAAAACTTCAACCCCAGCTCCACCAGTTCC
GATCAACACGTACCGAGAAAAAAAGGAAGAAGAACGACAACAGAAGAGGGATGAAGAAGGATTACACC
TTCTGACCTTGCTATTGCAATGTGCAGAAGCCGTATCCGCTGATAATTTAGAAGAGGCAAATAAAATGCTA
TTAGAAGTATCCGAACTCTCCACACCGTTCGGCACATCTGCACAGCGTGTTGCAGCATATTTCTCAGAAGC
CATGTCAGCAAGGCTGTTGAATTCATGCTTAGGAATATACGCAGCCCTACCAATGACCAGCGTCCCCATGC
TTTACACACAGAAAATGGCATCTGCTTTCCAGGTGTTTAACGGAATCAGCCCTTTCATCAAATTCTCACATT
TCACAGCCAATCAAGCTATACAAGAAGCTTTCGAGAGAGAAGACCGGGTACACATAATCGACCTTGATAT
AATGCAAGGTCTTCAATGGCCCGGTCTTTTCCACATCTTGGCTTCCAGGCCCGGTGGACCTCCTTACGTTC
GGCTCACGGGGCTTGGAACCTCGATGGATGCTCTTGAAGCAACCGGCAAACGACTATCCGATTTTGCCGA
ACGATTAGGCCTTCCTTTCGAGTTTTTACCAGTTGCTGATAAAGTTGGAAACTTAGACCCTGAGAAATTGA
ATGTGAGCAAAAGAGAAGCTGTAGCAGTTCATTGGTTGCAACATTCCCTTTACGATGTTACTGGCAGCGA
CCCTAATACACTCTCCCTCTTACAAAGGTTGGCTCCAAAGGTGGTGACGGTGGTGGAGCAGGATCTGAGC
CACGCGGGTTCATTCCTGGGGAGGTTTGTAGAGGCAATACATTACTACTCAGCTCTATTTGACTCACTGGG
CGCGTGTTACGGGGAGGAGAGTGAAGAACGACACGTGGTGGAGCAACAACTATTATCAAAGGAGATAC
GTAATGTACTAGCCGTAGGAGGTCCATCAAGGAGCGGGGATGCTAAATTCAACAACTGGAGAGAGAAGC
TTCAACAATCTGGATTTAGATCCTTGTCTCTTGCAGGTAATGCTGCTGCACAAGCCACTCTTTTACTCGGAA
TGTTCCCGTCTCATGGATACACTTTGGTGGAGGATAATGGAACACTTAAGCTTGGATGGAAAGATCTTTGC
TTGTTTACTGCTTCTGCATGGAGACCTAATTCATTGCATGCTGCACCAGGTTCTCGTCACTTCTCTCGTCCTA
ATATGGATTAA

Amino acid sequence:
MAKALPVSGENCTGVNVVGDSSGEKTSKQLFHCENGKMVRKRAASDMEIQTGAGEEHRYLRRPAMIGGSHS
QVGDSRVCGNSNFGHGMNSNLTTTMTMTTQVSNYSTMQMLPSSTNLCGVTSRGGPGIDTGFSNSTPNLTYT
DAITSHHQPQGTQTQNNNSQSPSVCVFSGLPLFPPDRNRQNSGLLLQQPPAAAAAVVSSPLTTGRIDSMEDST
SATAWIDSIIKDLINSSAQVSVPQLIQNVREIIHPCNPYLASLLEYRLRSLTSNNNGGADQNDPMECWRRKESLPA
QLAGLQQAQNNANLLQHNILSLPDSSNNQYLNWDIALPNSHNAPVAPSHNQHQQLGGNNPTATDLSFVTLS
PQVQQQQQQQQQESPHSHSQQQAAVDLDQQQKQQQSSSSLSPTSVADNSAKTKTSTPAPPVPINTYREKKE
EERQQKRDEEGLHLLTLLLQCAEAVSADNLEEANKMLLEVSELSTPFGTSAQRVAAYFSEAMSARLLNSCLGIYA
ALPMTSVPMLYTQKMASAFQVFNGISPFIKFSHFTANQAIQEAFEREDRVHIIDLDIMQGLQWPGLFHILASRP
GGPPYVRLTGLGTSMDALEATGKRLSDFAERLGLPFEFLPVADKVGNLDPEKLNVSKREAVAVHWLQHSLYDVT

GSDPNTLSLLQRLAPKVVTVVEQDLSHAGSFLGRFVEAIHYYSALFDSLGACYGEESEERHVVEQQLLSKEIRNVL
AVGGPSRSGDAKFNNWREKLQQSGFRSLSLAGNAAAQATLLLGMFPSHGYTLVEDNGTLKLGWKDLCLFTAS
AWRPNSLHAAPGSRHFSRPNMD-

SlGRAS16 Solyc03g123400.1.1

Nucleotide sequence (ORF):
ATGGCCATTGAAGAACCTGAGCCTAATTCCAACTCCGACCCCATATCAGAGTGGCTAGCTAGTACTTTATC
CGATGTTCCATCCTTCTTCCATGAACCTTACAGTTATGCCGATGACTTAAATTTCTACGCGGACCCATGGTG
GGTTCCTGATCAAGAGGATCAGATCGTTAATCACAACATCATCATCGACAATACTTGTAACTCATTCAACA
TTAGCTCCCCAGTCAACACAGCTATTAACAATATCCCTTTGGAGCCCATCATTTTGGATCATCCACAGCCGG
TGGATCTGTCTAAGAAAAGGAAAAAATCTGATCAAAATCCAAAGGCGTCAAAGAAGAATCATAAACTTCA
GATTAATGAGGCAGCACATGCTCCTACTATAGTTGATCAAGAAGGAGTGCAATTAAAAAAATCAATAGGA
CCGAAGAGAGTAACAACAGGAAATAACAGTAACAACAAAGAAGGAAGATGGGCAGAACATTTGCTCAAC
CCCTGTGCTGCTGCAATCACTGTAGGTAACATGAACCGCGTGCAACATCTGTTGTACGTTCTCCATGAACT
GGCGTCATTCACAGGCGATGCCAACCACAGGCTAGCTGCTCATGGACTCCGCGCCTTGACACATCATCTGT
CTTCCCCTGGCTCATCATCAGCATCCTCTGGAACTATTGGAGTTACTAATTTCTCTTCTGCCAATCATAAATT
CTTCAGGGACTCGTTGATTAATTTCATCGACGTTAGTCCCTGGTTCCGAATCCCCAATAACATTGCGAATTC
ATCTGTTCTCCAAATAATTGGACAACAGGATCGGCTAAAGAACCTTCATATCCTTGATATTGGAGTTTCTCA
TGGTTTTCAATGGCCAACTCTTCTTGAAGAATTGACTAGGCGGTCAGGTGGACCGCCGCCACTGGTTCGCC
TGACCGTTATTACACCAACCACAGAAAACGGAGAATTGACAGGAACTCCATTTGTGATCGGGCCACCTGG
CTATGATTTTTCCTCGCAACTACTAGCCTATGCAAAGGCTATCAACATCAATCTACAGATCAACAGACTGG
ATAATTTTCCTCTTCAAAACCTCAATTCCCAAATCATAAATTCTTCATCTGATGAAACCTTAGTCATCTGTGC
TCAATTTAGACTGCATAACTTGAATCATACTATCCCGGATGATCGAACAGATTTATTGAAGATATTGAAGA
GTTTGGACCCGAAAGGATTAGTTCTCAGCGAGAATAACACGGAGTGCAGCTGCAACAGCTGTGGGGACT
TTGCGACCACGTTCTCAAGGAGAGTGGAGTACTTATGGAGGTTTTTGGATTCCACGAGTGTTGCCTACAA
AGGGCGGGAGAGCGAAGAAAGGAGGATGATGGAAGGAGAAGCAGCAAAGGCATTGACGAACATGGGA
GAAATGAATGAAAGAAAAGAGAAATGGTGTGAGAGAATGAGGAGCGCTGGTTTTGTAAAAGCGGTGTT
TGGTGAAGATGCCATTGACGGAGCTCGGGCTTTGCTGAGGAAGTATGACAGCAATTGGGAGATAAGGGT
GGAGGAGAAAGATGGCTGTGTGGATCTATGGTGGAAAGGACAGCCAATTTCATTCTGCTCATTATGGAA
GATTTAG

Amino acid sequence:
MAIEEPEPNSNSDPISEWLASTLSDVPSFFHEPYSYADDLNFYADPWWVPDQEDQIVNHNIIIDNTCNSFNISSP
VNTAINNIPLEPIILDHPQPVDLSKKRKKSDQNPKASKKNHKLQINEAAHAPTIVDQEGVQLKKSIGPKRVTTGN
NSNNKEGRWAEHLLNPCAAAITVGNMNRVQHLLYVLHELASFTGDANHRLAAHGLRALTHHLSSPGSSSASS
GTIGVTNFSSANHKFFRDSLINFIDVSPWFRIPNNIANSSVLQIIGQQDRLKNLHILDIGVSHGFQWPTLLEELTRR
SGGPPPLVRLTVITPTTENGELTGTPFVIGPPGYDFSSQLLAYAKAININLQINRLDNFPLQNLNSQIINSSSDETLVI
CAQFRLHNLNHTIPDDRTDLLKILKSLDPKGLVLSENNTECSCNSCGDFATTFSRRVEYLWRFLDSTSVAYKGRES
EERRMMEGEAAKALTNMGEMNERKEKWCERMRSAGFVKAVFGEDAIDGARALLRKYDSNWEIRVEEKDGC
VDLWWKGQPISFCSLWKI-

SlGRAS17 Solyc02g085340.1.1

Nucleotide sequence (ORF):
ATGGCCTATATGTGTACGGACAGTGGGAATCTAATGGCAATTGCTCAGCAAGTCATAAAGCAAAAGCAGC
AGCAGGAGCAGCTCCAACAGCAAGAGCAACAGCAGCAGCAGCAACAACAATTCTTGGGTGTTAATCCTTT
GTGTCTCAGCCCATGGACTTCCAATCACCAGACCTTAACTAATAGCCCCACTTTAGGATATGGACTTACCG
GGTCGGGTTTTGCAGACCCATTTCAGGTCGCCGGAGGAACAGATAGCGCCGAGCCGGGATTTCAGTTCCC
TAACTTGGAACATCATTCGACCGGGTTTAGGTTTGCTGATTTCGGCGGTGGACCGGGTGGTGAGTTTGAC
TCAGATGAGTGGATGGAGAGCTTGATAGGCGGCGGAGATTCTACCGGAAGCTCTAATCTTCAATCTGGTT
GCGAAGCCTGGCAGACGAGTTCCGAATTCACTACCCTCTACGGAGATCCGTTCCCGAGTTGTCCGAATCG
ACTCAGTATCGGTTCTGCTCCTCCGCCTCCACCTTCTTCCGATCTCAATGGGGTCATTTTTTCGGAAACCCA
GAAAAATCTAAGCCCACTACAACCACAGACATCTCCATGGGTCGCACCATCTTCATCTCCTCCTCCAATTGT
TCAACCCGCGAGCAAAGAATCCAAGGTGGTATCAGTTGATGTGCAGCCATGTAGTTCCCCTGAAAGTTTTT
CGTCAAAACCATTGTTGAAATCCTTGGTGGAATGTGCAAGACTCGCTGAATCAGAGCCTGAAAATGTAGT
AAAATCATTGATTCGACTAAGGGAATCAGTTTCCCAACAAGGAGATCCAATGGAGCGAGTTGGGTTCTAT
TTCTTAGAAGCTCTTTACAATAGGCTTTCGTCGTGTCAAGCAGAGAGAACTCCGTCAATCTTTGGTACTGC
GCCGGAGGAGCTCACTTTGTCATACAAAGCCTTTAATGATGCATGTCCCTACTCAAAGTTTGCTCACTTAA
CGGCTAATCAGGCGATTCTTGAAGCAACTGAAAAAGCAACGAGGATTCACATTGTAGATTTTGGAATTGT
TCATGGTATCCAATGGGCAGCTTTTTTACAAGCTTTAGCAACTAGATCAGCTGGTAAACCAGTCAGTGTTA
GAATCTCTGGCATTCCATCAGTGGTATTGGGTAACTCTCCGTCAGCTTCACTTTTAGCTACAGGGAATCGT
CTTCGTGACTTCGCGAAGCTTCTGGATCTTAACTTCGAATTCGAACCAATATTGACTCCAGTTCAGGAACTT
AACGGGTCTAGTTTTCGAGTTGATCCAGATGAAATCTTAGCTGTGAACTTCATGTTGCAGCTGTACAATTT
ACTGGACGAAACAAATGTTGGTGTAAAAACTGCACTGAGTTTAGCCAAATCATTGAATCCAAGCATTGTG
ACATTGGGTGAGTATGAGGTGAATTTGAATGATGTTGGATTCCTGCAGCGATTTAAGAACGCACTGAAAT
ACTACTCAACTATTTTCGAGTCATTGGATCCGAGCTTGACCCGAGACTCCGCTGAGAGAGTACAAGTGGA
AAGACTGATACTTGGCCGGAGAATTGCCGGAGCGGTGGGCCTAGACGATGGCGGAACCAGAAGAGAAT
GTATGGAAGATAAAGAACATTGGAAAGAATTAATGGAAGGGGCTGGTTTTAAACCAGTAACACTTAGCC
ATTATGCAATGAGTCAAGCAAAAATTCTTCTTTGGAACTATAATTATAGTTCATCATTTGGATTAATTGATT
CTGCCCCTGGGTTTCTCTCCTTGGCTTGGAAAGATAATCCCCTTTTGACTGTTTCTTCATGGCACTAA

Amino acid sequence:
MAYMCTDSGNLMAIAQQVIKQKQQQEQLQQQEQQQQQQQFLGVNPLCLSPWTSNHQTLTNSPTLGYGL
TGSGFADPFQVAGGTDSAEPGFQFPNLEHHSTGFRFADFGGGPGGEFDSDEWMESLIGGGDSTGSSNLQSGC
EAWQTSSEFTTLYGDPFPSCPNRLSIGSAPPPPPSSDLNGVIFSETQKNLSPLQPQTSPWVAPSSSPPPIVQPASK
ESKVVSVDVQPCSSPESFSSKPLLKSLVECARLAESEPENVVKSLIRLRESVSQQGDPMERVGFYFLEALYNRLSSC
QAERTPSIFGTAPEELTLSYKAFNDACPYSKFAHLTANQAILEATEKATRIHIVDFGIVHGIQWAAFLQALATRSAG
KPVSVRISGIPSVVLGNSPSASLLATGNRLRDFAKLLDLNFEFEPILTPVQELNGSSFRVDPDEILAVNFMLQLYNLL
DETNVGVKTALSLAKSLNPSIVTLGEYEVNLNDVGFLQRFKNALKYYSTIFESLDPSLTRDSAERVQVERLILGRRIA
GAVGLDDGGTRRECMEDKEHWKELMEGAGFKPVTLSHYAMSQAKILLWNYNYSSSFGLIDSAPGFLSLAWKD
NPLLTVSSWH-

SlGRAS18 Solyc01g008910.2.1

Nucleotide sequence (ORF):
ATGTTACAAGATGATGGTTCATCATCAGTGACTTCATCATCACCACTTCAAAATTTCCCAATGATGTCAATT
TCACCTAGCTTTGTTGGTGGTGGTGGTTCACCATATCAGTGGCTTAAAGATTTGAAATCTGAGGATAGAG
GTTTGTATCTGATTCACCTTTTGCTTGCTTGTGCTAATCATGTAGCTAATGGTAATCTTGAAAATGCTAACA
TTGCACTTGATCAAATCTCCTATCTTGCATCTCCTAATGGAGATACAATGCAACGAATCGCCTCGTATTTCG
CTGAATCACTTGCTGATAGGATATTGAGATCTTGGAATGGGATTTACAAGGCGTTGAATTCGACTAAGTT
GAGGGTTGTATCTGAGGATATACTTGTTAAGAAGATGTTTTTCGAGTACTTTCCGTTCTTGAAAGTAGCTT
CTGTGATCGCTAATCAAGCGATCATAGAAGCTATGGAAGGTGAAAAAATGGTTCACATTGTTGATCTTAAT
GCTTCCGAGCCTTTACAGTGGCGTGCGCTCCTTCAGGATTTGAGCGCACGCCCTGAAGGACCCCCGCATTT
GAGGATTACGGGGGTCCATCAACAAAAACAAGTGTTGGAACAAATAGCACATGTTCTAACAGAGGAAGC
TGAAAAGCTTGATGTCCCTTTTCAGTTTCATCAAGTAGTTAGTAAATTGGAGAATCTTGATATCGAAAAGC
TACGAGTTAAAACCGGGGAGGCGTTAGCGATTAGCTCGGTTATGCAATTGCATACTCTTCTTGCACATGAT
GATGAACCCCAAAAGAAATCCCCTTTGGGTTTTAAGCATTTGAATGGTGTTCATTTGCAAAGGGCAATACT
AAACCAAAACACTCTAGGGGATTTGCTCGAAAACGAGATGATGACTCATAGTGTTTTTAGTCCGGGCAAT
GAATCAGCATCCTCATCTCCGTTATCATCGAGTGCATCAACAAAGATGGAAGGATTCCTCCACGCGTTGTG
GGGGTTGTCACCAAAGGTCATGGTGGTTACAGAACAAGACTCGAATCATAACGGTACAACCCTTATGGAA
AGGCTATCCGAGTCGTTGTATTACTACGCTGCATTGTTTGATTGTCTCGAGTTCACACTACAGAGAACATC
GTTAGAGAGGTTAAAGGTCGAGAAAATGATGTTTGGCGAGGAGATTAAGAACATTGTAGCGTGTGAGGG
AGGTGAACGTAGGGAGAGACACGAAAAGTTGGATAAATGGTTTCAAAGACTCGATGGAGCTGGTTTCAT
GAACGTGCCTTTGAGTTATTACGCGATGTTGCAAGCAAGGAGATTGTTGCAGAGCTATAGTTGTGAAGGA
TACAAGATTAAAGACGAGAATGGTAGCGTGGTGATCTGTTGGCATGATCGCGCGCTCTTCTCAGTCTCAG
CGTGGAGATGCAGGAGGTGA

Amino acid sequence:
MLQDDGSSSVTSSSPLQNFPMMSISPSFVGGGGSPYQWLKDLKSEDRGLYLIHLLLACANHVANGNLENANIA
LDQISYLASPNGDTMQRIASYFAESLADRILRSWNGIYKALNSTKLRVVSEDILVKKMFFEYFPFLKVASVIANQAI
IEAMEGEKMVHIVDLNASEPLQWRALLQDLSARPEGPPHLRITGVHQQKQVLEQIAHVLTEEAEKLDVPFQFH
QVVSKLENLDIEKLRVKTGEALAISSVMQLHTLLAHDDEPQKKSPLGFKHLNGVHLQRAILNQNTLGDLLENEM
MTHSVFSPGNESASSSPLSSSASTKMEGFLHALWGLSPKVMVVTEQDSNHNGTTLMERLSESLYYYAALFDCLE
FTLQRTSLERLKVEKMMFGEEIKNIVACEGGERRERHEKLDKWFQRLDGAGFMNVPLSYYAMLQARRLLQSYS
CEGYKIKDENGSVVICWHDRALFSVSAWRCRR-

SlGRAS19 Solyc01g009840.1.1

Nucleotide sequence (ORF):
ATGTTGACTGGTTGTTCTTCATCATTGGACTTGCCTCAAGCACATCATTTGCCTAATAACCAGTGGGACGAT
GATGCAGCTGAGCTGATCAGCTTACTTTGTGTTGAAGCAATTGGCTCAAGGAATGTTACTGGTGTTAATCA
ACTGATAGCTAGGCTAGGGCGGCTTGCCTATCTAAGAGGGTCTCCAGATTTGGTCTCAAATTTTCATATTA
TACCCACTCAGGTTCTCGATTGTTTAGATGTTTTTAGCAGTACGGTATTGAGGCTGTTGAATCAGGTTAGG
CCAATTTCAAAGTTCATCCATTTCACATCAAATGAGATTATGGTTAGAGCTTTTGAAGGCAAGGACCAGGC
TTACATTATTGATTTTGATACTGAGCAAGTCCTGCAGTGGCCTAACCTGTTTCAGAGTTTAGTTTCTAGGCA
CAAACCCCCCTCCCAAATTCATGTTAGAATTACTGGTATTGGAGAATCAAAGCAGGATCTTGGCATTGATC
TATGGATGCATCATGTAAAAGAGGGGGAAAATGTCGCAGTCTTCCATTGGACAGCCCTGGTAGGATTTAA

GACAGAAGAGTTGTTTGCTCGGGATATTAGAAACATTATCGCCCGTGAAGGACGAGATATGATTGAAAG
GCATGAATATTTTGGTAAATGGCGGAAGTTGATGGAACAAGGGGGGTTTCAAATGCACAGGGATTACAG
AAAGGGAACTGCTTCAGAGTCCAATTCTGTTGAAGATGTACTCATATGA

Amino acid sequence:
MLTGCSSSLDLPQAHHLPNNQWDDDAAELISLLCVEAIGSRNVTGVNQLIARLGRLAYLRGSPDLVSNFHIIPTQ
VLDCLDVFSSTVLRLLNQVRPISKFIHFTSNEIMVRAFEGKDQAYIIDFDTEQVLQWPNLFQSLVSRHKPPSQIHV
RITGIGESKQDLGIDLWMHHVKEGENVAVFHWTALVGFKTEELFARDIRNIIAREGRDMIERHEYFGKWRKLME
QGGFQMHRDYRKGTASESNSVEDVLI-

SlGRAS20 Solyc01g059950.1.1

Nucleotide sequence (ORF):
ATGAAGTGTCCCTTTTTCTTTAATGCAGTGGTGTCAGAAATGAAGGACCTGAGTAAAAATCTCTTTGAGTT
AGATACTAATGAAGTTGTGGTAGTTTACTCAGAGTATGTTCTTGGAGGCATGTTAACATGGCCTAATCACC
TTGAAGTTGTTCTAAGATTCATTGAAAGCCTCAATACTTGTGTGATAATAGTCATCGAAACTGAGGCAAAC
ATTAACGAGCCAATATTTATGGATCAATTTAATGAATCAGTGTTTCTGTATGCTACACTCTTTGATTGTTTG
GAGACATTCATGGGCCGAGACAATCAGCACAGGATGGAATTTGAAAGACTGGTTCTTAGAAAAATCATTC
ACAATGTGATCACTTGTGAAGGAGAAGGAAGGATCTTCCACAACGTCAGGCTGGAAGTATGGAGGGCTT
TGTTTGAAAATTTTGGTATTAAGGAAACAGAATTGAGTGAATTATCCTTGTACCAAGCAAATCTAATAGTC
GATCGATCTGCTCATGGCTTTTGCACTCTGGATATGGATGGAAATTGCCTTACTATTAAATGGAAAGGAAC
TCCAATAGTATTTGCTTCAGTACTTCTGTTCAAAGAGTTGTGTATTACTTCTGTGAATCTCTTCAAGAGAGA
ATTGATAAGGAGAAGGGAACATTATCACCTAATAGACATGAAGGTTGGGAAGAGAAACCATTTGATTTG
GACAAAGGTTCTGCAGAATGCGAAGCCTATTGCAGTTGTATGCTATCAATCATCACCTTTCTTCCAAGTGC
CGCAATTTACTGGAATTCAAGCCATCATAGATAATGTCAAGTCAGCAAAGGAGGGGTTCATTTGA

Amino acid sequence:
MKCPFFFNAVVSEMKDLSKNLFELDTNEVVVVYSEYVLGGMLTWPNHLEVVLRFIESLNTCVIIVIETEANINEPI
FMDQFNESVFLYATLFDCLETFMGRDNQHRMEFERLVLRKIIHNVITCEGEGRIFHNVRLEVWRALFENFGIKET
ELSELSLYQANLIVDRSAHGFCTLDMDGNCLTIKWKGTPIVFASVLLFKELCITSVNLFKRELIRRREHYHLIDMKV
GKRNHLIWTKVLQNAKPIAVVCYQSSPFFQVPQFTGIQAIIDNVKSAKEGFI-

SlGRAS21 Solyc01g059960.1.1

Nucleotide sequence (ORF):
ATGAAAGCCTTGTTCTTGGATTACACATTGTCTTATATGAATCTGACATCCTTATATTTCAAGATGGCAACA
GCCAAGAGGACAACTGTGAGCATTTTCATTATCTGTTTTAAAAAAAGAAAACCTCCAAGGTATCGCCGTCC
TAAGATGGTTCTATCGACAACAACCATCATGAAGATTGCCAAAGAACAATTAAAACTTTATACCTCACAGA
ATCACGATGCATATTCCATTATTTTTAGTCCTTTAATTGCGGATTTGGGCCTTTCTCCTCAAGTTACAGAGG
AAGTGGAGCTCGTTCTACTGCTTTTAGTTTCCGCTGAAATGCTAGCCAATCGACAATTAGATTGTGCAAGA
AAGTTGTTGAACTTATGCATCAACTTTTCTCTTTCAGCTGGTAATCCAGTTCAAAGAGTTGTCTATTATTTTG
CTGAATCTCTTCAGAAGAGGATCAATAAAGGGACAGGAATACCAACTATAGCAGCAAAAAATTGATGCAGT

GAATCCAAATATTATGGAAGATGTTCTCATGGATCCACGAACCGATGTTATTGAGACTGAACAAATGCTTC
CCTTCCGCAAAGTAACTCAATTTACTGGAATTCAATCCATCTTGGACAGTGTTAAATCATTCAAGAGGATTC
ATTTAATTGATTTTGGGATAAAAACCGGATCACAATGGACAATCCTTATGCAAGCTCTGGTTGGTAATGGA
GAATGTCCACCGGAACATCTCAAGATAACAGCAGTTGGAACCTCTTTGATTCGAATGCAAGAGGTAGGAA
AGAGATTGACATCTTTTGCAGACACCTTACATATACCTTTTTCCTTCAAAACAGTTGTATCTGACTTGAGAC
ATATCAACAAGGATTTATTCGAGTCAAAGGTTGGTGAAGTTGTGGCAATCTATTCTGACTCACGTCTTTGG
ACCTTGTTAGCATGGCCTAATCACTTGCAATCTCTTATACAAGTTTGCAAAAGTTTGGATCCATGTGTAATG
GTGGTAACTGAAATAGAGGCAAATACTAATACACCAATTTTCATCGATCGTTTCAATGAAGCATTGTTTTA
CTACAGTGCTATCTTTGATAGTCTTGAAACTTGCATAGGGTGGAACCATCAGTATAGAGCAGTAGCTCAA
GGAGTGTATATACGAAGGATTATTGAAAATGTAATTACATCCGAGGGAGAAGAGATGGTACATCGCCAT
GAGAAGCTTCGATCTTGGAGGACGTTATTTAAAAATTTTGGTATAGAGGAAGCAGAACTGAGTCACTCAT
CCTTGTACCAAGCAAAGTTGCTCGCAGAAAATTCTACATGCCATGGGCTTTGCAGTCTAGAAATGGATGG
GAAGTCTCTAATTATTAAATGGAAGGGAACCCCAATTAAGTCACTTTCTGCATGGAAGTTCCACCAATAA

Amino acid sequence:
MKALFLDYTLSYMNLTSLYFKMATAKRTTVSIFIICFKKRKPPRYRRPKMVLSTTTIMKIAKEQLKLYTSQNHDAYSI
IFSPLIADLGLSPQVTEEVELVLLLLVSAEMLANRQLDCARKLLNLCINFSLSAGNPVQRVVYYFAESLQKRINKGT
GIPTIAAKIDAVNPNIMEDVLMDPRTDVIETEQMLPFRKVTQFTGIQSILDSVKSFKRIHLIDFGIKTGSQWTILM
QALVGNGECPPEHLKITAVGTSLIRMQEVGKRLTSFADTLHIPFSFKTVVSDLRHINKDLFESKVGEVVAIYSDSRL
WTLLAWPNHLQSLIQVCKSLDPCVMVVTEIEANTNTPIFIDRFNEALFYYSAIFDSLETCIGWNHQYRAVAQGV
YIRRIIENVITSEGEEMVHRHEKLRSWRTLFKNFGIEEAELSHSSLYQAKLLAENSTCHGLCSLEMDGKSLIIKWKG
TPIKSLSAWKFHQ-

SlGRAS22 Solyc01g079370.2.1

Nucleotide sequence (ORF):
TCATATACAACAAGAATTCCATTCAATTTTTTTTTCTAGCTCTTTTCTCATGGATTCTTTTGCAAATTTATTTA
GAGAACCATTTCAATTTTTTACAACTATTCTTCTTAGTCTTCTTTTTCCTCTTTCTTTTCTTATAATTTCTAGGC
TATCTGTTGCACATTATCTTATTGATCTTGTAGGTTATTCAAAAACAGAAATTTCAAATAATTGTGATTTACT
AATCAAATTTTTTCTTCAAGTTAACCCTATTTTTCTACATGTTCTTGTTTCTATCATCAGTGTTGCTGCACTTT
CTCATACCTTAACCGGTGGTACCTCTTGCTTCATCAAGCGTACCCCAGAGCCCGTCTCGAGACTTCGATTAT
ACACCGCGTGGTTTTTCGTATGCATATTGCAAATTTGTGTTGGTTTAGGCATTGAAGGTAGTATAGCTGCT
GGAGTTGATGCTTATGAAATTGGTTATGAAAGGGGATTTTTGACAAGAGCTATATTCTTCTTGGGATTAAA
TGAGACAATGTTCTTTTGGTCCAATATTTTTGTGAAGCCAGTGGTGGATGACACAGTTTTTGGCATTCTAC
AAGAAAATAGATGGATTGAAAAGGCAGCATTGGCAATGAGTTTTGGTGGACTTTGGTGGTGGAGATTGA
GAGATGAAGTTGAGTTTTTAGTGGTTGTGGTGGAGAAAAAAAGAGACTTGTTGATTAGTATTGGATTGGC
TGATTTTGTTGGTTGGTGGTTGTATTATTTAACTGTTACAATTGGTATGATCAAAATTGTGAAAACTCTTGT
TTGGCTTGTTTATGTAATGTTTGGTAGAAGAGTTGTTGATTTTGACAATACTGATGGTGACTCTAGGAACA
ATGATGAGAAGGATGATCAGTTTCATCTTAAAGGATATGAGAGAAGTGAGTTAGTGGTCAATGGTATTGA
AGAAGGTTGTGTAGATATTGATTCTCTGTACTCCATTTGTGGACATAACAATGAAGAGAATACATCTGATG
AACAAGTTAGAACCAACTTATTGGAAGATCAGCAACAAAGCCTATCAGATTGGACGCAAAATCATTTCCTC
ACCAACACAGTCATGCCCTTGCAACCGGTCCAAATCCAATCTACTATTGAAGCACCAACAAATGATGATTT
CCCGCCTCAAGTACCAGCACCTGCCAACTCTAGCAATGAAAAACCAAAGCCGTTTTCATTAGCATCTTTGG

AGCTACTGAGCAATTATGGCAGACTGAGCAAGAAATCCAGTGAGGAAAACTTGTGCACTAATGCACTAAG
TTGTGAGGCTCACTTAGAGAATAGCCACAAGTTGCCAATGAAAGAGATCTTGAGGGCTGCAGGAGAAAG
GTACATACAGTACTCCACCCAAAGGCTAGATGGTTTTTCTATGTTTATTCACCCTTATGGTTCGGCACTCTC
AGGCCTTTGTATGGAGGAAACAAGGGATGTGGAGCTTGTCCACCTCCTCCTAGCTGCAGCAGAAGAAGT
GAATAATCAACAATTCCATCTAGCTAGCAAATTGATTTCTCGTTGTATGTGGGTGGCATCTGATTCAGGTA
ATCCAGTCCAGAGACTTTCTTACTATTTTGCTAAAGCTTTAGAAGAAAGGATTGATCGATCAACTGGAAGA
TACACATGTACGGATGAAGATCACCACCTCAAGTATATTAAAATTATGTCATTAGGTACCAACTCTGCATTC
TTGACCTGCCACCAACTAATTCCTTTCAGTCAAGTGATGCAATTTGCAGGAGTTCAAACAATTGTTGAAAA
TGTCAGAAGCGCAAAAAAAATTCATTTGATTGATTTTAATATCAGAAGTGGAATTCAGTGGATAGTCTTGA
TACAAGCTCTTGCAGAAAAAGGTGACAGTCCAATTGAGCTTCTTAAGATAACTGCAGTTGGAAGCATAGA
AAAAGAGAACTTTGAAGCAACAAGCAACACATTACACAGTTTCACCAAGTCCTTAGGCCTGCCATTTTCAT
TTGATATAGTTTTTGTCTCGGACATGAAAGATTTCAAGAAAGAGTCGGTCAATATTAAAACAGATGAGACT
GTGGCTGTTTACTGTAACTCTATACTAAGGACAATGCTATCAAAACCAGATTGTTTGGATAATTTGATGAA
AGTAGTCAGAAGTATTGGACCAACAATTATTGTTGTTGGCGAAGTTGAAGCAAACCATAATTCACCATCAT
TTTTAAACCGCTTTATTGAGACTCTTTTCTTTTACAGTGCATTTTTTGATTGCTTCGAGGACTGCATGGATCG
ATGCAGTCCATGTCGAACAACGATTGAAGGGGTATATTTTGGTGAAGGTATTCGGAACATCGTGGCGGCT
GAAGCTGAGGACAGGTTCACCAGGAATGTGAAGCTTGAAGTATGGAGAGCGTTCTTTGCAAGGTTTGGT
ATGGTGGAAGAAGAGCTTAGTGAGTCAGCTTGGTATCAAGCTCATCTGATTCTCAAGCAATTTGCCCAAG
GAAGTTCTTGTGATCTTCAAAAGGATGGGAAAGGCCTCATTATTGGGTGGAAGGGAACACCAATTCATTC
TCTATCCATTTGGAAGTTCTTGTAA

Amino acid sequence:
MDSFANLFREPFQFFTTILLSLLFPLSFLIISRLSVAHYLIDLVGYSKTEISNNCDLLIKFFLQVNPIFLHVLVSIISVAAL
SHTLTGGTSCFIKRTPEPVSRLRLYTAWFFVCILQICVGLGIEGSIAAGVDAYEIGYERGFLTRAIFFLGLNETMFFW
SNIFVKPVVDDTVFGILQENRWIEKAALAMSFGGLWWWRLRDEVEFLVVVVEKKRDLLISIGLADFVGWWLYY
LTVTIGMIKIVKTLVWLVYVMFGRRVVDFDNTDGDSRNNDEKDDQFHLKGYERSELVVNGIEEGCVDIDSLYSIC
GHNNEENTSDEQVRTNLLEDQQQSLSDWTQNHFLTNTVMPLQPVQIQSTIEAPTNDDFPPQVPAPANSSNEK
PKPFSLASLELLSNYGRLSKKSSEENLCTNALSCEAHLENSHKLPMKEILRAAGERYIQYSTQRLDGFSMFIHPYGS
ALSGLCMEETRDVELVHLLLAAAEEVNNQQFHLASKLISRCMWVASDSGNPVQRLSYYFAKALEERIDRSTGRY
TCTDEDHHLKYIKIMSLGTNSAFLTCHQLIPFSQVMQFAGVQTIVENVRSAKKIHLIDFNIRSGIQWIVLIQALAEK
GDSPIELLKITAVGSIEKENFEATSNTLHSFTKSLGLPFSFDIVFVSDMKDFKKESVNIKTDETVAVYCNSILRTMLSK
PDCLDNLMKVVRSIGPTIIVVGEVEANHNSPSFLNRFIETLFFYSAFFDCFEDCMDRCSPCRTTIEGVYFGEGIRNI
VAAEAEDRFTRNVKLEVWRAFFARFGMVEEELSESAWYQAHLILKQFAQGSSCDLQKDGKGLIIGWKGTPIHSL
SIWKFL-

SlGRAS23 Solyc01g079380.1.1

Nucleotide sequence (ORF):
ATGAATAAACAACAAGAAGAACTGAATGAGCATGCTGCCCATTCTGCAGGTCAGTTAAGTCGAGGAAAA
ACAAAGCTCAACAATCTAGAAACAGGAAGCAATGTGGAGGAGGTACCTTTTCTATCTCTAGCTCCTTTTGA
ATTGTTGAGTAACAATCCGAGCAGGTGCAGGAGAAGAGATAAAGTTAGAGATGTGAATGGCGGAGCCA
ACATGCACCAGAAGCTCTCAGCTACACAAATCATGAGGCTAGCCGGAGAAAGATTCATCCAGTTCTCTTCC
AACAAGTTCATCAACATAAACAATCTTTTGCACCCCTATGGTTCTGCTTTCTCAGACCTTTCTCCTGAAGAT

AATCAAAATGTAGAACTTGCTCAAATCCTCCTAGCTGTAGCTGAGAAAGTTTCTGACCAGCACTACGATCG
TGCAAGGAAATTTCTGAGTCAATGCCAACTACTTGCATCAAACACAGGTAACCCTGTGCAAAGAGCAGTC
TTTTATTTTGCACAAGCACTTGGAGAGAGGATCGATAGAGAAACAGGAAAATTAATGCCAAAAATCTGCA
CGGAGACAGATGAACTTAACAGTATCTGTGTTGCATCAAGATTCCATGCCTCATTCGTAGCATTACATCAA
GAAGTTCCATTCACCCAAATAGTGCATTTCACAGGAATTCAAGCCATACTAGAAAGTGTAGCAATGAAACC
CAAAGTTCACCTAATTGATTTCCTTATCAGAACAGGGGTACAATGGGCATCCTTGATCCAAGCAGCAGCAG
AGAGAAAAGACAACCCAATCGAACATATGAAGATCACAGTTATCGAATCAGTCAATAAAGAAAGAGTAAT
AGAAACAGGCAAGTCTTTAGAGAGTTTCGCTAATTCCTTGAACTTCCCCTTCTCATTCAAGATCATCTTTTT
ACCAGATAGAAACCAACTGACCGAAGACCACTTCGACATAAAACCTGACGAAGCAGTAGTAATCCATGCA
GCATTCTTTCTACGAGCAATGATCAGCATACCCAACTGTTTAGAAACTGCAATGAGAGTTGTAAGAAGGCT
ACAACCATCACTAATGGTAGTATCAGAAGTAGAGGCAAATCTGAATTCACCTCTATTCGTGAACCGATTCA
TAGACACTTTATTCTACTACAGTGCATTATTTGATTGCCTGGAAGATGTAATGAAGCGGGATGATCAACAC
AGGATGACACTGGAAGTGTCATACGGAGCAGGGATTCGGAACGTAGTAGGAAATGAAGGGGAGGAAAG
GGTCATTAGAAGTGTGAATCTTGATGTGTGGAGATCATTCTTGAAGAGGTTTGGAATGGAGGAAGTAAA
GCTAAGTGACACATGTTTGTATCAAGCTAACTTGATTATTCAACAGTTTGCTTGTAAGAATTCTTGCAGTGT
GGATGTTAACAGAGGGTCTTTCATGATCAGCTGGAAAGGAACTCCAATCTATTCCACTTCTGCTTGGAAAT
TCCATTGA

Amino acid sequence:
MNKQQEELNEHAAHSAGQLSRGKTKLNNLETGSNVEEVPFLSLAPFELLSNNPSRCRRRDKVRDVNGGANM
HQKLSATQIMRLAGERFIQFSSNKFININNLLHPYGSAFSDLSPEDNQNVELAQILLAVAEKVSDQHYDRARKFLS
QCQLLASNTGNPVQRAVFYFAQALGERIDRETGKLMPKICTETDELNSICVASRFHASFVALHQEVPFTQIVHFT
GIQAILESVAMKPKVHLIDFLIRTGVQWASLIQAAAERKDNPIEHMKITVIESVNKERVIETGKSLESFANSLNFPF
SFKIIFLPDRNQLTEDHFDIKPDEAVVIHAAFFLRAMISIPNCLETAMRVVRRLQPSLMVVSEVEANLNSPLFVNR
FIDTLFYYSALFDCLEDVMKRDDQHRMTLEVSYGAGIRNVVGNEGEERVIRSVNLDVWRSFLKRFGMEEVKLS
DTCLYQANLIIQQFACKNSCSVDVNRGSFMISWKGTPIYSTSAWKFH-

SlGRAS24 Solyc01g090950.2.1

Nucleotide sequence (ORF):
ATGAAGGGGATGCCCTTACCCTTTGAATTTGAGGGGAAGGGGGTGTTAGAATTAGACGTTTTGTTCAATAA
AGATAGTATCTTGAATTCTTGGAGTAACCACAATACCAGCAAAGAAAGTAGTAGTTGTTGTTACTTTGTAAAT
AGTCCAAGTGCTGTCCTGGATACTATAAGGAGTCCAAGGCCTATTAATTCCTCTTCAACACTGTCTTCATCTTT
GGGTGGTGGTGGTGGTGGTGGAGGCACTGCTTCCACAGACACTGCAGGAGGAGGTGTGGCGGCAGTTTC
TGCAAACCCATCTTCTAAATGGCAGCAGCAGCAGCAAGACAATACTACTGCTACAAGCTCCAATGTGGGAGC
TGAATCTGAGCTTCAACAAGTTCAACCCCCATCTTCTCTTGAGATGGGTGCTGCTGCCACGGGAGGGGATAA
ATGTGCTATGGAGGAGTGGGAAGGAGGGTTATCAGAGTCCGTAATGGCTTCACCTTGCCAAGAACAGTCTA
TACTAAGGTGGATCATGGGAGATGTCGATGACCCATCCATGGCTAACTTGAATAAGGTGTTGCAGGTTAGTG
GTACAGGGGACTATGAATTCAATGGAGGATTTGGGGTTGTGGATCAAGGTTTTGGTGTAGACCCAGTTGGC
CAAATTGGTAGTTTTATGCCTGCAATTAGCTCATCTGTTTCTATGTCAAGTTCAAGTTTTCCTACCAACAGAAT
GAACAGTGACAAGATTGGCTTGGTTTCTAACCCACCCACAAATCTCCCTCAAAATCCAATCTTTCCTTCATTG
TCTAACAATCTTGGGCCAATTGCATTTGGCCAGACACAGCAACAGCCGTTTGAGAGCACTGATTTGAAGCCT
CATGCTTTCAATTCACAGTTCTTGATAAACCAGCACCAAACACAGATTCCTCAGAACCCATCGTTTCTTTTGC

CACTACCATTTGCACAACAGGAACAAAATCTTGCCTTGCCACCGCAGGCGAAACGACACAACCCCGGGACC
ATGGGAGGGCTTGAACCGGGCTCTCAGATCTCCAAAGGATTGTTTCTAGATGCAGGGCACCAGCAGCCAAC
ACCATCTCAGGGGCTTGCTCATCAGCTCCAGCTACTTCCCCATTTTAGGCCAGGAGCAATGGGGACAAAGCC
AAAGATGGTGGGGGAAGAAATGGCTCAGTTTCATCAACTACAACAACAGCAGCAGCAGCAACAACAAGCG
ATTATTGACCAGCTATTCAAAGCTGCAGAGCTGGTCCAGACAGGGAATCCGGTACTCGCGCAAGGGATATTG
GCGCGGCTCAATCACCAGCTCTCTCCAATTGGTAAGCCTTTCTATAGGGCTGCTTTTTATTGCAAGGAAGCTT
TACAATTGCTACTCCATGCCAACACCAACAACTTGAACCCATCAATGGCCTCTTCGCCTTTTAGTCTCATTTTC
AAGATTGGTGCCTATAAGTCCTTCTCTGAGATCTCACCAGTTGCACAGTTTGCTAATTTTACTTGTAACCAAGC
CCTGCTTGAGGTCTTGGATGGGTTTGAAAGAATTCATATTGTAGATTTCGATATCGGTTATGGCGGGCAATGG
GCCTCCCTTATGCAAGAGCTTGCCTTGAGAAGTGGTGGCGCACCTACTTTGAAAATAACTGCATTAGCCTCA
CCATCCACACATGACCAGCTAGAGCTTGGACTCACCAGAGAAAATTTGATCCATTTTGCTAGTGAAATCAATA
TGGCATTTGAGTTTGAAATTTTAAGCATTGATTCTTTAAATTCAACGTCATGGTCACTGCCTCCTTTAGTCTCA
GAGAATGAGGCAATTGCTGTCAATCTTCCAGTTAGCTCGCTTTCGAGCTATCAGTTGTCCCTTCCATTGGTTC
TTCGTTTTGTGAAGCAGTTGTCACCTAGGATTGTGGTTTCTGTGGATAGAGGTTGTGACCGGACTGATCTAC
CATTTCCAAACCATGTAATTCAAGCCCTTCAGTCCTACTCAAATCTTCTTGAGTCACTAGATGCTGTAAATGTG
AATTTCGATGCCCTCCAAAAGATTGAAAGGTTCTTGCTCCAACCAGGAATTGAAAGAATTGTAATGGGTCGT
TTTCGATCTCCTGAAAAGACGCAGCATTGGAGGTCACTGTTTTTTGTCATCTGGATTCGCCCCATTATCTCTCA
GCAATTTTACAGAATCACAGGCCGAGTGTGTAGTCAAGAGGACTCCTGTTAG

Amino acid sequence:
MKGMPLPFEFEGKGVLELDVLFNKDSILNSWSNHNTSKESSSCCYFVNSPSAVLDTIRSPRPINSSSTLSSSLGGG
GGGGGTASTDTAGGGVAAVSANPSSKWQQQQQDNTTATSSNVGAESELQQVQPPSSLEMGAAATGGDKCA
MEEWEGGLSESVMASPCQEQSILRWIMGDVDDPSMANLNKVLQVSGTGDYEFNGGFGVVDQGFGVDPVG
QIGSFMPAISSSVSMSSSSFPTNRMNSDKIGLVSNPPTNLPQNPIFPSLSNNLGPIAFGQTQQQPFESTDLKPHA
FNSQFLINQHQTQIPQNPSFLLPLPFAQQEQNLALPPQAKRHNPGTMGGLEPGSQISKGLFLDAGHQQPTPSQ
GLAHQLQLLPHFRPGAMGTKPKMVGEEMAQFHQLQQQQQQQQQAIIDQLFKAAELVQTGNPVLAQGILAR
LNHQLSPIGKPFYRAAFYCKEALQLLLHANTNNLNPSMASSPFSLIFKIGAYKSFSEISPVAQFANFTCNQALLEVL
DGFERIHIVDFDIGYGGQWASLMQELALRSGGAPTLKITALASPSTHDQLELGLTRENLIHFASEINMAFEFEILSI
DSLNSTSWSLPPLVSENEAIAVNLPVSSLSSYQLSLPLVLRFVKQLSPRIVVSVDRGCDRTDLPFPNHVIQALQSYS
NLLESLDAVNVNFDALQKIERFLLQPGIERIVMGRFRSPEKTQHWRSLFFVIWIRPIISQQFYRITGRVCSQEDSC-

SlGRAS25 Solyc02g092370.1.1

Nucleotide sequence (ORF):
ATGGATACTTTGTTTAGGTTAGTTAGCCTTCAACAACAACAACAACAACAATCTGATCAATACTCTTTTAAC
TCCAGCAGAACTTCAAGCAGCTCTAGATCTTCTAACAAACAAAACAGCACATACAATTATCATCCACATCA
TAATCATCAAGACGAAGAATGCTTCAACTTTTTCATGGATGAAGATGATTTCTCTTCTTCTTCTTCTAAACA
CAACAACTATCCTCCTCCTCATTACAATCAATATCAACAAATCTCCACACCCACAACTACAAGCAGTACCCC
AACACATCAATCTCAATCTCAATATGATCATCAATTCTCCCCAGCACGTGATTTAAATCTCGAATTCGCTTCC
TCATTTTCTGGAAAATGGGCCACAGACATTCTTCTAGAAACTTCTCGTGCCATAGCCGATAAGAACAGTAC
ACGTGTCCAACAGCTCATGTGGATGTTGAATGAGCTGAGCTCCCCCTATGGAGATACCGAACAAAAATTG
GCTTCTTATTTTCTTCAAGCATTATTTAGCCGTATGACGGATTCTGGCGAACGATGCTATCGTACCTTATTA
TCCGCTTCGGATAAAACATGTTCGTTCGAGTCAACGAGGAAATTAGTTTTGAAATTTCAGGAAGTTAGCCC

TTGGACAACTTTTGGTCACGTTGCATCCAATGGTGCAATTATGGAAGCGTTAGAAGGTGAATCGAAGTTG
CATATAATTGATATTAGCAACACGTATTGTACTCAATGGCCTACTTTACTAGAAGCACTAGCAACCCGCAC
CGACGAAACACCGCATCTCCGCCTCACGACGGTGGTTGCAGCTGCTAGCGGAGGTGCAGCGTCGGTGCA
AAAAGTAATGAAGGAGATTGGTAGTAGAATGGAAAAATTTGCTAGGCTTATGGGCGTACCGTTTAAATTC
AACGTGATTCACCACGTGGGAAATTTGTCTGAGTTGGATATGGGTGCGTTAGATATTAAAGAAGAGGAA
GCGCTAGCAATTAATTGTATTGGTGCGTTACATTCGGTTACGCCAGCTGGCAATCGAAGGGATTATTTGAT
ATCATTATTTAGGAGGTTACAACCTAGGATTGTTACAATTGTTGAAGAAGAGGCTGATCTTGATGTGGGC
GTTGATGGTTTCGATTTTGTTAATGGTTTTCAAGAATGTTTGAAATGGATTAGGGTTTATTTTGAATCATTA
GACGAGAGTTTTTCGAAAACAAGTAATGAACGTTTGATGCTAGAGCGACAAGCAGGACGTTCGATTGTTG
ATTTACTAGCATGCCCACCATCAGAGTCAATGGAGAGGAGAGAAACGGGGGCAAAATGGTCACATCGTA
TGCATGCAGGAGGGTTCAGTCCGGTTTTATATAGTGATGAAGTTTGTGATGATGTTAGAGCTTTGTTGAG
AAGGTATAAAGATGGGTGGTCGATGGGACAGTGTGGCGGAGATTCCGCCGGCATATTCTTGTCGTGGAA
GGAACAGCCGGTGGTGTGGGCCAGTGCATGGAAGCC
TTAA

Amino acid sequence:
MDTLFRLVSLQQQQQQQSDQYSFNSSRTSSSSRSSNKQNSTYNYHPHHNHQDEECFNFFMDEDDFSSSSSKH
NNYPPPHYNQYQQISTPTTTSSTPTHQSQSQYDHQFSPARDLNLEFASSFSGKWATDILLETSRAIADKNSTRVQ
QLMWMLNELSSPYGDTEQKLASYFLQALFSRMTDSGERCYRTLLSASDKTCSFESTRKLVLKFQEVSPWTTFGH
VASNGAIMEALEGESKLHIIDISNTYCTQWPTLLEALATRTDETPHLRLTTVVAAASGGAASVQKVMKEIGSRME
KFARLMGVPFKFNVIHHVGNLSELDMGALDIKEEEALAINCIGALHSVTPAGNRRDYLISLFRRLQPRIVTIVEEE
ADLDVGVDGFDFVNGFQECLKWIRVYFESLDESFSKTSNERLMLERQAGRSIVDLLACPPSESMERRETGAKW
SHRMHAGGFSPVLYSDEVCDDVRALLRRYKDGWSMGQCGGDSAGIFLSWKEQPVVWASAWKP-

SlGRAS26 Solyc02g092570.1.1

Nucleotide sequence (ORF):
ATGCAATTTACTGAGAGTTTACCTCCACAAGTTTCATCATTTTCAAGTTTGATGATGAACAAGGATAGTAG
TACTAATAATAATCAGATCCAACGCGCCCGCCCGTGGCCTGGTTTTCACACATCGAAGAATTTGGGGAACA
TTGGGGATACTAATTGCATGGAACAGTTGTTAGTCCATTGCGCCAATGCCATCGAGAGCAATGACGCAAC
TTTAGCACAACAAATATTATGGGTCCTTAATAACATTGCCCCTCCTGATGGTGACTCGAACCAACGCCTAA
CTTGTGGATTCCTACGAGCCCTAATTCTTCGTGCTGCGAAAATTGGGACTTGTAAATTACTCACTGCTAATC
TGAATCATTCCATGGAAACTCATAAATTCTCCATTATTGAGCTTGCAAGTTTCGTTGATTTAACCCCTTGGC
ATCGGTTCGGGTTCACGGCTGCTAACGCTTCTATATTAGAAGCTGTGGAAGGTTACTCTATTATTCACATT
GTTGATTTCAGCTTAACTCATTGCATGCAAATTCCAACACTTATAGATGCAGTTTCTACTCGAGTAGAAGG
ACCTCCGTTGATCAAACTAACGGTAGCTGGTGCAACCGAAGATTTACCGCCTATGCTTGATCTTTCTTACG
AAGAATTAGGTGCTAAATTGGTAAATTTCGCAAGGTCACGAAATGTCATGATGGAATTTAGAGTAATTCCT
TCGAGTTCTTCGGATGGTTTTTCAAATGTAATCGAACAGCTACGTGTTCAAAATCTAATACGTCCGGATAA
TGGCGAAGCACTAGTAATAAACTGTCACATGATGCTTCATTACATACCGGAGGAAACAACGGTAATTTCC
AACTCATTCCGTGCCATGTTTCTTAAGGCCATTCGGAATTTGGACCCAACAATTGTTGTGCTAGTAGATGA
AGACGCGGATTTTACTTCTAATAATCTGGTGTGCAGATTAAGATCGGCGTTTAATTATTTATGGATACCTTA
TGATACAGTGGATACATTTCTTCCGCGAGGGAGCAAGCAAAGGCAGTGGTATGAAGCGGATATTTGCTG
GAAGATTGAGAACGTGATAGCTCAGGAGGGAATACAACGACTAGAACGGCTGGAGCCGAAGAGTCAGT

GGGTGCAACGGATGAGAAATGCAAATTTCAGAAGCAGTGGATTTAGCGAAGAGGGAGTTACGGAAGTG
AAGAACATGTTGGATGAACATGCAGCTGGGTGGGGGCTAAAGAGGGATGAAGAATATCTTGTGCTTACA
TGGAAAGGACATAATGTTGTATTTGCTACTGCTTGGGTTCCTCTTTAG

Amino acid sequence:
MQFTESLPPQVSSFSSLMMNKDSSTNNNQIQRARPWPGFHTSKNLGNIGDTNCMEQLLVHCANAIESNDAT
LAQQILWVLNNIAPPDGDSNQRLTCGFLRALILRAAKIGTCKLLTANLNHSMETHKFSIIELASFVDLTPWHRFGF
TAANASILEAVEGYSIIHIVDFSLTHCMQIPTLIDAVSTRVEGPPLIKLTVAGATEDLPPMLDLSYEELGAKLVNFARS
RNVMMEFRVIPSSSSDGFSNVIEQLRVQNLIRPDNGEALVINCHMMLHYIPEETTVISNSFRAMFLKAIRNLDP
TIVVLVDEDADFTSNNLVCRLRSAFNYLWIPYDTVDTFLPRGSKQRQWYEADICWKIENVIAQEGIQRLERLEPK
SQWVQRMRNANFRSSGFSEEGVTEVKNMLDEHAAGWGLKRDEEYLVLTWKGHNVVFATAWVPL-

SlGRAS27 Solyc02g094340.1.1

Nucleotide sequence (ORF):
ATGGGGAGTTTAAAGAACGACATAATAAAAAATGAGGTTGGCAGTGAAATAACAAGTTTTAAGGACCAG
AATTCTTACATCAAACTGCTTCCAGAGGACTCTCTTGCCTCTTCTGAATCCAAGAAAGTCACTCCTATCTCCT
CGGACTTTGAACTCGATTGTGGCAGCTTAATACCTACCAGCCTCACTTTCCCTCCCGGAGGTGGCGACGAT
GATGATGTTGAAATCCAATCTCCTGATAACTCAATCTGGGAGTCCTTCTTTGCTGATCAGCTGGAGGCAGA
TTTCATGATCTCTTCCCCCGTCAGGAACCTTTCCTCGACTTCGACTTTCTGTACTACTACTACTACTCACAAT
AACAACACATACACACATCATAATCAGGGAATTCATGGGCAGAGCATGATGATGTGCTCCCCTCCTCGCTC
TCCCTTGAGACCTAATAATTACAACTCCACTAATAAAGGCAAGGGACTTAGCCCCTTCCAAAAAGTTTTCA
ACTCTCCTAATAACCAGTTCATGCAAATTGAGAGCTTTAACCTGCCAGCTCTTGAGAGTTTCTTAGATGATG
ATTTGGCATCCGAATATTCCACACTCAAAGTTTCAGATGTTGGATCCTCTTCCGAGTCTCTCTCAGTAATTC
CTGATTTTTTAGAGTGCCTGGCGTTGCCAAATTCATCATCCAATACGTCCGCTAGCTTTATGGGATCATTAC
TCAGCAACACATCTGTCGGACAAGTAGACGATGAGATTTTCCAGACGGGGTCTATTGCACCTCTGTCACAA
CAGCTACATCAAGAACGACACCATGAGAAGCAACAAAAACAAATACCAACACATGTTCAACTACCATCAA
CACAACAACAATATACGCAAATGATCAATCACAATTTGGTTGTTGCTGCACCTGATCAGATGCAGGAACA
GGACAGTGGCTTACAATTGGTGCACCTTCTCCTTGCCTGTGCTGAAGCAGTCTCCAAAGAGGACTATATGT
TGGCAAGAAGGTATCTCCACCACCTGAATCGAGTAGTCACCCCAATCGGTGACTCAATGCAACGAGTGGC
TTCTTGCTTCACGGAAGCTCTTACTGCTAGACTTGCCGCAACATTAGCCACTAAACCCAGCACCTCTGTCCC
AAAACCTTTCAATCCTTTTCCTCCTAATTCCCTTGAAATCCTCAAGATCTATCAGATTCTTTACCAAGCCTGC
CCTTATGTCAAATTTGCTCATTTCACAGCTAATCAAGCCATATTTGAAGCATTTGAAGCAGAGGAGCGCGT
TCATGTTATTGATTTAGACATCCTTCAAGGTTATCAGTGGCCGGCTTTTATGCAGGCCTTAGCAGCCCGAC
CTGGCGGTGCTCCTTTCCTTCGTATCACAGGAGTGGGTTCGTATCCGGAAGCGGTCAGGGAAACAGGTCG
ATGTTTGACAGAACTGGCGCAGTCCCTCCACGTTCCTTTTGAATTTCACCCGGTGGGGGAGCAGCTTGAG
GATTTGAAACCGCACATGTTCAACAGAAGGATAGGTGAGGCTTTGGCGGTGAATTCGGTGAATAGACTCC
ATCGAGTCCCAGGGAATTGTATTGGGAACTTGTTAGGCATGATCAGAGACCAAGCTCCAAATATAGTAAC
GATCGTGGAGCAAGAGGCGAGCCATAACGGACCGTATTTCCTGGGTCGGTTCCTGGAGGCACTGCACTA
CTATTCGGCCATCTTTGATTCCCTGGACGCTACATTTCCAGGGGATTCATCACAGAGGGCAAAATTGGAAC
AGTACATATTTGGACCTGAGATAATGAACATAGTTTCGTGTGAGGGAATGGAGAGAATGGTGCGGCACG
AGAGGTTAGAAAAGTGGAGGAGGGTGATGGAAGGAAAAGGTTTTAAAGGAGTAGCACTGAGTGCAAAT
GCAGTCACACAATCCAAAATATTACTGGGTCTCTACTCCTGTGATGGTTATAAATTGACAGAGGACAATGG

TTGCTTGCTTTTGGGATGGCAAGATAGGGCCATTCTTGCTGCCTCTGCATGGCGATGCTGA

Amino acid sequence:
MGSLKNDIIKNEVGSEITSFKDQNSYIKLLPEDSLASSESKKVTPISSDFELDCGSLIPTSLTFPPGGGDDDDVEIQS
PDNSIWESFFADQLEADFMISSPVRNLSSTSTFCTTTTTHNNNTYTHHNQGIHGQSMMMCSPPRSPLRPNNY
NSTNKGKGLSPFQKVFNSPNNQFMQIESFNLPALESFLDDDLASEYSTLKVSDVGSSSESLSVIPDFLECLALPNS
SSNTSASFMGSLLSNTSVGQVDDEIFQTGSIAPLSQQLHQERHHEKQQKQIPTHVQLPSTQQQYTQMINHNLV
VAAPDQMQEQDSGLQLVHLLLACAEAVSKEDYMLARRYLHHLNRVVTPIGDSMQRVASCFTEALTARLAATLA
TKPSTSVPKPFNPFPPNSLEILKIYQILYQACPYVKFAHFTANQAIFEAFEAEERVHVIDLDILQGYQWPAFMQAL
AARPGGAPFLRITGVGSYPEAVRETGRCLTELAQSLHVPFEFHPVGEQLEDLKPHMFNRRIGEALAVNSVNRLH
RVPGNCIGNLLGMIRDQAPNIVTIVEQEASHNGPYFLGRFLEALHYYSAIFDSLDATFPGDSSQRAKLEQYIFGPE
IMNIVSCEGMERMVRHERLEKWRRVMEGKGFKGVALSANAVTQSKILLGLYSCDGYKLTEDNGCLLLGWQDR
AILAASAWRC-

SlGRAS28 Solyc03g110950.1.1

Nucleotide sequence (ORF):
ATGATTCATGATATGGCTAATGTTTTTCTATCACTTGAGCCTTGTAATGGTGATCAAATTGGTTATGACCCT
ATGGAAAATAGCCTGTATCTTACTCATCACAAAGAACTGTCTTATTCTCTTAATCCTTACACATCTGTTTTGA
AGAGAAATGCTCCTACTAATAACATGATCATTTCATCTTTATCTAATGACAGTGCGAGTTTTAAGAGGTTA
AGGAGAACCCCAAGTCTTGGTGAGTCTTTTGGAAGCAATACCACTTTTTATAGTACTGAAAGCAGCAGCA
CTGGTGGCAGTTTACCGAGAATTGGTAGTAGTAATAGTGTGAATAGCTTGTCATTGCAACCAGGGATTCA
TTTTCGCGATCATGTTTGGGCGTTGAACCAAAGGTATCTTGCTGCTGAGGCTTTTGAAGAGGCAGCAGCT
GATATAATTAATCAGGAGGAGGAAAATGGGGAAGGTATGAAGTTAGTACAGCTTTTAATTACTTGTGCTG
AAGCTGTGGCTTGTAGAGATAAGTCTCGTGCATCGGTGTTGTTATCGGAGCTACGTGCTAGTGCATTGGT
ATTTGGGACGTCTTTCCAGCGTGTAGCGTCCTGTTTCATGCAGGGGTTGTCTGATAGGCTGGCTCTGGTGC
AGCCGCTTGGCACGGTTGGTTATGTTGCTACCCCTGCTATGAATAAGACAGACATTGCCTTGGAGAAGAA
AGAAGAAGCTTTGAGGCTGCTTTATGAGATATGTCCACATATTCAGTTTGGTCATTTTGTGGCTAATTGCTT
GATATTGGAAGCCTTTGAGGGAGAGAGTTTCATTCATGTGGTTGATTTGGGAATGAGCCTTGGTTTGCCT
CATGGTCATCAATGGCGGCGTCTTGTTCAGAGTCTTGTTAATCGCCCTGGACAGCCCCCACGCCGCCTTAG
GATCACTGCTGTTGGACAGAATATCGAGAAATTGCAAATTATTGGAGATGAGCTTGAGGACTATGCAAGA
AGCCTTGGTATAAATTTGGAGTTTTCAGCTGTGGAAAGCAACTTGGAAAACCTTAAACCAAAGGACATCA
AAGTATACGATGGCGAGGTCCTCGTGGTTAACAGCATTCTTCAGCTCCATTGTGTGGTGAAGGAAAGCCG
CGGTGCACTCAACTCTGTTTTGCAGGTAGTTCATGAACTTTCTCCAAAGATTCTAGTCCTTGTGGAGCAAG
ACTCGAGCCACAACGGACCCTTTTTTCTCGGGAGATTCATGGAAGCTCTGCATTATTACTCTGCTATCTTCG
ACTCCCTTGATGTCATGCTGCCCAAGTATGACACCAGGCGAGCCAAGATAGAGCAATTTTACTTTGCAGAG
GAGATTAAGAACATTGTGAGCTGCGAGGGACCAGCAAGAGTGGAAAGGCACGAGAGGGTGGACCAATG
GCGTAGGAGGATGAGCCGAGCAGGTTTTCAAGCAGCGCCTATTAAGATGGTGTCACAAGCCAAACAGTG
GCTTGCGAAAGTGAATGGACATGAAGGATTCACCATCACAGAAGAGAAAGGCTGCTTGGTTCTTGGATG
GAAATCGAAGCCTATCGTGGCTGCCTCTTGCTGGAAATGCTGA

Amino acid sequence:
MIHDMANVFLSLEPCNGDQIGYDPMENSLYLTHHKELSYSLNPYTSVLKRNAPTNNMIISSLSNDSASFKRLRRT

PSLGESFGSNTTFYSTESSSTGGSLPRIGSSNSVNSLSLQPGIHFRDHVWALNQRYLAAEAFEEAAADIINQEEEN
GEGMKLVQLLITCAEAVACRDKSRASVLLSELRASALVFGTSFQRVASCFMQGLSDRLALVQPLGTVGYVATPA
MNKTDIALEKKEEALRLLYEICPHIQFGHFVANCLILEAFEGESFIHVVDLGMSLGLPHGHQWRRLVQSLVNRPG
QPPRRLRITAVGQNIEKLQIIGDELEDYARSLGINLEFSAVESNLENLKPKDIKVYDGEVLVVNSILQLHCVVKESRG
ALNSVLQVVHELSPKILVLVEQDSSHNGPFFLGRFMEALHYYSAIFDSLDVMLPKYDTRRAKIEQFYFAEEIKNIVS
CEGPARVERHERVDQWRRRMSRAGFQAAPIKMVSQAKQWLAKVNGHEGFTITEEKGCLVLGWKSKPIVAAS
CWKC-

SlGRAS29 Solyc04g011630.1.1

Nucleotide sequence (ORF):
ATGTATCGGTTCAGGAATCTACTCGATGAGACTGTGGTGGTAAATAGTCCTAGAGATATTGTATTGAATCT
CATCCGGAAGTTAAATCCTGATGTTTACATACAGGGGATCGTAAACGGTGCTTATAATGCTCCCTTCTTTAT
CACACGATTCCGGGAGGCACTTTTTCATTACTCGTCCGTGTTTGATATGCTTGAAGCTAATATTCCCCGTGA
AATTCCTGAGAGATTGCTGGTTGAAAAGTTAATATTTGGCAGGGAGGCAATGAATGTTGTAGCGTGTGAA
GCTGCTGAGAGGATTGAGAGACCAGAAACATATAAACAATGGCAGGTCAGAAATACAAGGGCTGGTTTT
AGGCAGCTTCCTTTGAACGAGGAGATTTTGAGGATGGCAAAAGATCGTGTAAAGGCGTACCACAAGGAT
TTCGTGATTGATGTAGACGGTCATTGGCTACTGCAGGGATGGAAAGGTCGTATCATGTATGCAGCTTCAA
CCTGGAAGGCAGCTCTTTAA

Amino acid sequence:
MYRFRNLLDETVVVNSPRDIVLNLIRKLNPDVYIQGIVNGAYNAPFFITRFREALFHYSSVFDMLEANIPREIPERL
LVEKLIFGREAMNVVACEAAERIERPETYKQWQVRNTRAGFRQLPLNEEILRMAKDRVKAYHKDFVIDVDGH
WLLQGWKGRIMYAASTWKAAL-

SlGRAS30 Solyc05g053090.1.1

Nucleotide sequence (ORF):
ATGGGGCAATATCATGAGGCAGGATCTGCAGTTAAATTAGAGGATGAAGATTGCTCATTTTTCGCTGATC
CGAATCTGATTAATAATCTCAGAGTTAATGATTACTTTCATGACGATTATGATCCAAATTTGATTAATAATC
TTAGAGTTAGTGATAATTTAGTCAATATTAGCAGTCCATTTCAATCTGATGTCGAACGAAATGCTTTAGTTC
CATTAACTGCAGATGACTTTCATGAGGATTATGATTTCAGTGATGGGGTTCTAAAGTATATAAATCAGATG
CTTATGGAAGAGGATATTGAAGAAAAGACTTGCATGTTTCAAGAATCTGCAGCTCTTCAAGCTGCAGAAA
GATCGTTTTACGAAGTTATTGGAGAGAAGTATCCTTCTTCCACAAATCATGAGAAATCGTCCACTTTAGGT
CAGATTGAACGTTATGCTATGGGCCACTGTAGTGGAAATGATGGTCGTGATGGTTTACTAGGCCCAAATT
GGATTCTTGATCTTGGTGAGGATGATGTGTCACATGTTCCCGATGATGTTGCTTTGGATTCAACTTCTCGG
TCATCCAACAGTTTGTCAGGCACTGTCCCGGATGTGCCTGTGGATTCTCCTGTGAGCACACTTAGGATTCC
TGACATATTTAGTGGCGGAGAGTCTGTTATGCAGTTTAAGAAAGGGGTGGAGGAGGCAAGTAAGTTCCT
TCCTACAGGCAATAGCTTGCTTGCTGATGTGAGGTATCATGTAGTGGGAAAGGAGCTACGGTATAAAGA
GAGGAAAGATGCAGTCGTAAAGGTTGACAAGTATGGGGAAAAGCGATATACTGAAAGGTCGAGGGGGA
AGAAAAATACCTTTCATGAAGATATAGTAGATTTAACGGAAGGGAGAAATAACAAGCAGTCTGCAGTGTT
TTCTGAATCAACTGTCCGATCGGAAATGTTTGATAGGGTGCTGCTATGCAGTGCAGGGAAAAATGAATCT

GCTCTTCGTGAAGCCTTGCAGGCTATATCAAGGCAAAATGCATCGAAGAATGGCCCTTCAAAGGGATCTA
ACGGTAAGAAGTTACAACGAAAGAAAAAAGGGGGTAAAAGAGATGTGGTAGACTTGAGAACACTTTTGA
CGCTCTGTGCACAAGCTGTTGCTGTAGGTAATCAACGGACCGCAAATGAGCTTCTGAAGAAGATAAGGGA
AAGTTCTTCTCCTATGGGGGATGGAATGCAGAGGTTGGCACATTATTTTGCTGACGGTCTTGAGGCACGG
ATGGCTGGTTCCGGTACTCATATCTATAAAGCCCTTATTACACGGCCTGTATCAGCAGCTGATGTCTTGAA
AGCGTACCATCTACTGCTTGCTGCCTGCCCATTCAGGACTATGTCTAGCTTTTTCTCAAATAAGACAATTAT
GAATCTAGCTGAAAAGGCTTCAACAGTACATATTATAGATATTGGCATTATGTGGGGTTTCCAATGGCCTG
GCCTCATACAACGTCTCGCTTCTAGACCAGGTGGACCTCCCAAGCTTCGTATAACTGGGATTGATTTTCCA
AATCCTGGTTTCCGACCAGCAGAAAGGGTTGAGGAAACTGGTAGGCGGTTAGCAAATTATGCTGAGAGC
TTCAAAGTTCCTTTTGAGTTCAATGCTATAGCGCAAAAGTGGGAAACAGTTAAACTCGAGGATCTTAAGAT
CAATAAGGGTGAGGTTCTTGTGGTAAACTGTCTGTACCGGTTTAGGAATCTGCTTGATGAGACTGTGGTG
GTCAATAGTCCAAGAGATGTTTTTCTGAATCTTATCCGGAGGTTGAATCCAGATGTTTTCATACTGGGAAC
TGTAAATGGTGGTTATAATGCCCCATTCTTTATCTCACGATTCCGTGAGGCTCTTTTTCACTACTCATCCTTG
TTTGATATGCTTGAAACTATTATTCCCCGGGAAGTGCACGAGAGAATGCTGGTTGAAAAGAATATAATTG
GTCAGGAGGCAATGAATGCCATCGCGTGCGAAGGTGCTGAGAGAATTGAGAGACCAGAAACATACAAG
CAATGGCAAGTTAGAATTCTGAAGGCTGGTTTTCGGCAGCTTCCTTTGGATGAGGAGATTATGAGAATGA
CAACAGAACGATTCAAGGTGTACGACAAGAATTTCATTATTGACGTAGATAGTGAATGGCTACTGCAGGG
CTGGAAGGGTCGTATTGCATCTGCACTTTCAATGTGGAAGGCAGCATATTGA

Amino acid sequence:
MGQYHEAGSAVKLEDEDCSFFADPNLINNLRVNDYFHDDYDPNLINNLRVSDNLVNISSPFQSDVERNALVPLT
ADDFHEDYDFSDGVLKYINQMLMEEDIEEKTCMFQESAALQAAERSFYEVIGEKYPSSTNHEKSSTLGQIERYA
MGHCSGNDGRDGLLGPNWILDLGEDDVSHVPDDVALDSTSRSSNSLSGTVPDVPVDSPVSTLRIPDIFSGGES
VMQFKKGVEEASKFLPTGNSLLADVRYHVVGKELRYKERKDAVVKVDKYGEKRYTERSRGKKNTFHEDIVDLTE
GRNNKQSAVFSESTVRSEMFDRVLLCSAGKNESALREALQAISRQNASKNGPSKGSNGKKLQRKKKGGKRDVV
DLRTLLTLCAQAVAVGNQRTANELLKKIRESSSPMGDGMQRLAHYFADGLEARMAGSGTHIYKALITRPVSAAD
VLKAYHLLLAACPFRTMSSFFSNKTIMNLAEKASTVHIIDIGIMWGFQWPGLIQRLASRPGGPPKLRITGIDFPN
PGFRPAERVEETGRRLANYAESFKVPFEFNAIAQKWETVKLEDLKINKGEVLVVNCLYRFRNLLDETVVVNSPRD
VFLNLIRRLNPDVFILGTVNGGYNAPFFISRFREALFHYSSLFDMLETIIPREVHERMLVEKNIIGQEAMNAIACEG
AERIERPETYKQWQVRILKAGFRQLPLDEEIMRMTTERFKVYDKNFIIDVDSEWLLQGWKGRIASALSMWKAA
Y-

SlGRAS31 Solyc05g053420.1.1

Nucleotide sequence (ORF):
ATGTGTGATCATTCTCATCAACAATCTCCATTGCCTTCATCATTCAAGATCCTGAAAGATTATGACACAACA
AGGTTCATGAAAAGATCAAAAACAGAACAGAACATCATTGTTAGTGCTGAATGTATCAAGGAAAACAATG
GATCCATGTTATCTACAGACGAAATCCTTCGTTTAGGTGCTGAAAGGTTTATCCAATCGTTTACCCTTCGTG
GTGATCAAGAACCATACATGTTTAACCATCCATTCGCGAGCTCATTGCTTGACCTTCACGAGGAAGACACT
AGAAATGTGTCACTAGTCGAAAATCTTCTTGCTTCTGCTGAGAAAGTAGGCCAGGAACAGTTTGATCGCG
CTAGAAGGATTCTCAACGAGTTCTGTGATAAGCTGTGTTCCAACACTGGAAATGCAGTTCAGAGATTAGT
ATACTACTTTTCTCAAGCACTTCATGAGAGAATAGATAGGGAAACGGGGAGAGATACATCGAAAGGAAA
TGAAATGAAACGATTAGTACAGCATATCGAGCATTCATTGATGAACTTAAACCTCACTATGATTGCAGCAC

ATCAGAACATACCTCTGTCTCAGGTGTCTCAGTTTGCTGCTGTTCAAGCCATTATAGATCATGTAGGAGAA
TCGAAAAAGGTTCATATAATCGATCTCAAGATAGGTAGTGGTCTGCAATGGACAATCTTGATGCAAGCTCT
GGTCTCACATCAAATCAAACATCTCAAGATAACTGCTTTATGTACCAACCTAAAACATAAAATTGAGGAAG
CACGCGAACGATTGATGGACTTTGCAAAGTCCTTGAACTTGCCCTTCTCTTTCAACATTGTGATGGTTAAG
GATATGACAGAGCTAAAGAAAGAAGACTTTGAGATAGATGACGATGAATCAATTGCTATCTTCGCGCAAT
ACATCCTTATGTGGATGTTAGCGCGACCAGACAAGCTTGACTCTTTGATGAGAGTTATCAAAGGTATAAAT
CCTCGAGCATTGATCGTTGTAGAAGTGGAAGCAAACCATAACTCACCAGTATTCGTGGACCGGTTTGTGG
AAGCCCTGTTTTTCTATGGTGCATTTTTCGACTCGTTGGAGGATTGCATGAAGAACGACGAACGAAACAG
GACAGCTACAGAACTGGAACACTTGAGCCAGGGAATAAGAAGCATTGTGGCAACTGAGGGTGAGGAAA
GGACCATTAGACATGTAAAAGTAGACGTATGGAGGGCGTTTTTCGCGCGATATCGTATGGAGGAAATGG
AGCTAAGTATGTCATCATTGTATCAGGCAAATTTGGTGCTCAAGAATTTTGCTTGTGGGAGTTGTTGCACA
CTGGAAATGAACAAAAATTGCCTACTAATTGGATGGAAAGGAACCCCTCTTAGTTCACTTTCTGCTTGGAA
ATTTTCTTGA

Amino acid sequence:
MCDHSHQQSPLPSSFKILKDYDTTRFMKRSKTEQNIIVSAECIKENNGSMLSTDEILRLGAERFIQSFTLRGDQEP
YMFNHPFASSLLDLHEEDTRNVSLVENLLASAEKVGQEQFDRARRILNEFCDKLCSNTGNAVQRLVYYFSQALH
ERIDRETGRDTSKGNEMKRLVQHIEHSLMNLNLTMIAAHQNIPLSQVSQFAAVQAIIDHVGESKKVHIIDLKIGS
GLQWTILMQALVSHQIKHLKITALCTNLKHKIEEARERLMDFAKSLNLPFSFNIVMVKDMTELKKEDFEIDDDESI
AIFAQYILMWMLARPDKLDSLMRVIKGINPRALIVVEVEANHNSPVFVDRFVEALFFYGAFFDSLEDCMKNDER
NRTATELEHLSQGIRSIVATEGEERTIRHVKVDVWRAFFARYRMEEMELSMSSLYQANLVLKNFACGSCCTLEM
NKNCLLIGWKGTPLSSLSAWKFS-

SlGRAS32 Solyc05g054170.2.1

Nucleotide sequence (ORF):
TGATTCATGATTTTTTTTGGCAGTTGGTTATGACATTCGACGTATCTGCTGAAAGCTTCTGAGACTTTGTGA
CTACTTTTAACGAAGAGACTTCGTTAGCTTGGAGCAGTTGTTCAGCATTTTAGCCAATTTGAAGAGAGTGC
GTGACTGATCCCAATATGCGCGCATCCCAGATACCTCAATCATCAGGCGGAGTTCACAAGTTGTGCCATCA
GCCAACGGCGAACTTTGAGCAATATTATAGCCCTTACCATGTAGTTAACAACAATTCCAGCGATACTAGCA
GCTCGGGGACACAGCTCTCTTATCAGACACAGAATGATAAGTTTTTCACTCTTGACTCACTTCCTGATGCTG
GTTACGTTAGCTATGATTCGCCTCCTGCTGTAAGCGTCTCGTCCAACTGGAGTCCGTTCTCTCCTCAGTGTT
CTCAGTCGTACATATCGGATCAGCACCATTCCTCAGACAACACTTATGGTTCACCTTTGAGCGGGTGTTCA
GTAATTAATGATGGGAATGAACTGAAGCATGTGCTAAGGGAGATGGCGAATAATTTGCTAGGGCCTGGT
TTTGATATTGATGAAGACAGCAGTTGTTCTTTCAATGGTGAGGTCTCAAAACCTTCAAAGTGGAATCGCGT
ATTGGAAATTGCACCGAGCTTGGACATGAAAGAGCTGCTTCTTGCGTGTGCTGAAGCAATATCGGATGTC
GAAGTCACAGCTAGAGATGCTCAGATGAATGTCTTAGAGCAAAAGGTATCGGATGCTGATGTGACAGCT
AGAGATGCTCTGATGAATGTCTTGGAGCAAAAGGTATCAGTTTCCGGGGAACCTATGCAACGATTAAGCG
CATACATGTTGGAAGGACTCAAAGCAAGAATATATTCTTCGGGAAGTAACATCTACAAAATGCTCAAGTG
CAAGGAACCAACTGGTTCAGAATTGATCTCTTACATGCAAGTCCTCTATCACATCTGCCCCTACTACAGATT
CGCTTACACATCCGCCAACGTTGTGATTGAAGAAGCCATGAGGAATGAGAGCAGAATCCATATAATTGAT
TTTCAAATTGCACAAGGAAGTCAATGGGTGTTCCTTATGCAAAATCTTGTGTTGATGATTCCCAATCAGCT
CATGCACGCGGTGGTGGACTTCACCTAGTAGGCGAAAGGCTAGCGAAAGCTGCTGCATCATGTGGAGTG

CCTTTTGAATTCCATGCTGCTGCTATATCGGGCTGTGAGGTTCATCTAGAAAACCTTCAAATGAGACATGG
AGAAGCCTTGGCAGTTAACTTCCCGTACGTGCTGCACCACATGCCAGACGAGAGTGTAACCACTGTAAAC
CATCGAGACCGTCTACTCAGATTGGTTAAGAGCTTGTCCCCGGAAATAGTCACCCTTGTTG

Amino acid sequence:
MRASQIPQSSGGVHKLCHQPTANFEQYYSPYHVVNNNSSDTSSSGTQLSYQTQNDKFFTLDSLPDAGYVSYDS
PPAVSVSSNWSPFSPQCSQSYISDQHHSSDNTYGSPLSGCSVINDGNELKHVLREMANNLLGPGFDIDEDSSCS
FNGEVSKPSKWNRVLEIAPSLDMKELLLACAEAISDVEVTARDAQMNVLEQKVSDADVTARDALMNVLEQKV
SVSGEPMQRLSAYMLEGLKARIYSSGSNIYKMLKCKEPTGSELISYMQVLYHICPYYRFAYTSANVVIEEAMRNES
RIHIIDFQIAQGSQWVFLMQNLVLMIPNQLMHAVVDFT-

SlGRAS33 Solyc06g009610.1.1

Nucleotide sequence (ORF):
ATGGGTTCGAGTTCAAAATTCTACGTTTCAGTCTCAAAAGACTATGTTGTGAATATGTTAAGAGAGTCATT
TCAATGCCTTACCATTATCCTCCTCAGCCTTCTCCTTCCTCTCTCCTTTATTGTCCTCGCTAGGCTGTCAGTTA
CTCGTTATCTCATTGCCACATCAGATTACACGGAGCCCAATACACTACTTGTCAAGCTCTTTCTCTATGCAA
ATCCTATATTTCTTCGTCTACTAACTCCACTTGTCACCATTTCTGCTTTGACTCAATGCTTGACAGGACGAAC
TCTAACTGATCACATCATGATTTCAAAGTCTCATCTTTATATGTCGTGGTTAATACTTTTCACATTTCAAATT
TGTGTGAGCATAGGCATAGAAGGAAGTATAGCTGTTGGAATAGATGGTAGTACTAGTTTTAGTCACCAAA
GATTATATTTATTGACGAGAAGTATGTTTTTCATGGGATTGCATGAGACAACGTTGTTTTGGTCCAAGAAA
GTAGTGAAACCTGTGGTTGATGACACAATTTTTGGCGTTGAGAAGGAGGATAGGTATGTTGAGAAGGTG
GCTATGGCAATGAGTTTTGGTATTTTGTGGTGGTGTAAGTTAGGGGATGAAGTTGAGTCTTTAGTGGTTG
TGGCTGAGATGAAAAGAGACTTATTTGGTAGTGTTGGTTTGGTTGATTTTGTTGGTTGGTGGTTGTATTAT
GTGACTGTGGCAATTGGTATGGTTAAGGTTGTGAAGGGACTAATTTGGCTAAATTTTGTGTTGTTTTGTGG
GAATATGGTGGTTGGTTCAGACAATGCAGGCTCTTGTACAAGAAATGATGAAAAGATGGATCAAATCCCA
ATCCCCACCATTGATTTCAGTGTTTTTCAGGATGGTCACAATCATGTTCAAGAGTGTGGGAGAAATGAATT
GATCAACAATGGCGCCGTAGATCCTTGGTTTGAATTGGACAGTGAGGATCTTGATTCTCTTTACACAGTAT
TTGACCTTAAATATCAAGATAATACAGCAGACAAACAAGTACAGTCACTGGAAGATCAGCAACAAAGCAT
TTCAGATCACTGGACCCAAAGTGATGATTCTTCCAACATGGACTTGCCCCTGCAGTTTATCCAAATTAAAAC
GACCCCCGAAGCATCTGATGGGATTCAGCCTCAGGAGCCAAGGCTTGACAATTTCCATGGAGAGAAAACA
AATTCCATTTCTTTAGCATCTTTGGAGCTCTTGAACAATTGTGGGAGATTGTTCAAGAAACCAAGTGAGGA
AAACTTGAGCAACGTATTACTCAGCAACGAGGCTCGTGTAAGTAACATCTCTAAGTTGTCAACAGAAGAA
ATCTTACGAGTTGCAGGAGAAAGATATATCCAGTACTCCACCCAAAGGGTAGATGGTCTTTCTATGTTTAT
ACATCCATATGCTTCATCACTTTCGGGCCTTTCCATAGAGGAAACAAAGGACATGGAACTTGTTCACCTCC
TTCTAGCTGCTGCAGAAGAAGTGGATCAGCAGCAATTTCATTTGGCTAGCCAATCTATTGCACACTGCCTG
TGGAAGGCATCTGCTACAGGTAATCCAATCCAGAGACTTTGTTTCTATTTTGGTGAAGCTTTACAAGAAAG
GATTGATCGAGAAATAGGAAGGTCCCCATGTTTCGAAAGAAAGTTAAGATTTTTAAGCACTCTGGCATTA
GGTAATACTCCTGAATCCTTAACATGCCATACAGAGATTCCCTTCAGTCAAGTAATGCAATTCGCGGGAAA
TCAGGCAATAGTTGAATATGTTAAAGGTGCAACCAAGATCCATTTGGTTGACTTTAATATTAGAAGTGGAA
TTCAGTGGACAGGATTGATGCAAGCTCTTTCCGAACGACGTAACTGTCCAATTGAGCTTCTGAAAATTACA
GCCATTGGACACCAAGAAAAACAGAAAATAGAAGAAACAGGCAAGAGATTACAAAGTTTTGCCAATTCCT
TGAATCTTCCCTTTTCATTTGATATGATCTTTATGTCTGACATGAAAGATCTCAAGGCAGAATCAGTCAATC

TTAAAGCAGATGAAACTGTAGCTGTCTATTGTTACACTGTACTTAGAACAATGATCTGCAGGCAAGATTAT
TTGGACAACACGATGCGAGTAATCAGAGGACTAAGACCATCTGTGGTTGTTGTCTGTGAAGTTGAGGCAA
ACCTTAATTCACCTTCATTTTTGAACCGTTTCGTGGAGGCACTTTTCTTTTATAGTGTGCTTTTTGATTGCTTT
GAAGACTGCATGGATAGAGACAATTTGGTCAGAAAGAGGATTGAAGTATTTCATATTGGTGAAGGAATC
CGGAATATGGTTGCAGCTGAGGGTGCAGAAAGGTTCACTCGGAATGTAAAGCTTGATGTATGGAGAGCA
TACTTTGCAAGGTTTGGAATGGTGGAAATGGAGCTCAGCGAGTCATCTCGATATCAAGCAAATCTGATTC
TAAAGCAATTTTCCCATGGCAGTTCTTGCATTGTTCAAAAGGATGGAAAGGCCCTCCTAGTTGGATGGAA
GGGAACACCCATTGAATCAGTTTCTATTTGGAAGTTCTTGTAA

Amino acid sequence:
MGSSSKFYVSVSKDYVVNMLRESFQCLTIILLSLLLPLSFIVLARLSVTRYLIATSDYTEPNTLLVKLFLYANPIFLRLLT
PLVTISALTQCLTGRTLTDHIMISKSHLYMSWLILFTFQICVSIGIEGSIAVGIDGSTSFSHQRLYLLTRSMFFMGLHE
TTLFWSKKVVKPVVDDTIFGVEKEDRYVEKVAMAMSFGILWWCKLGDEVESLVVVAEMKRDLFGSVGLVDFV
GWWLYYVTVAIGMVKVVKGLIWLNFVLFCGNMVVGSDNAGSCTRNDEKMDQIPIPTIDFSVFQDGHNHVQ
ECGRNELINNGAVDPWFELDSEDLDSLYTVFDLKYQDNTADKQVQSLEDQQQSISDHWTQSDDSSNMDLPLQ
FIQIKTTPEASDGIQPQEPRLDNFHGEKTNSISLASLELLNNCGRLFKKPSEENLSNVLLSNEARVSNISKLSTEEIL
RVAGERYIQYSTQRVDGLSMFIHPYASSLSGLSIEETKDMELVHLLLAAAEEVDQQQFHLASQSIAHCLWKASAT
GNPIQRLCFYFGEALQERIDREIGRSPCFERKLRFLSTLALGNTPESLTCHTEIPFSQVMQFAGNQAIVEYVKGATK
IHLVDFNIRSGIQWTGLMQALSERRNCPIELLKITAIGHQEKQKIEETGKRLQSFANSLNLPFSFDMIFMSDMKDL
KAESVNLKADETVAVYCYTVLRTMICRQDYLDNTMRVIRGLRPSVVVVCEVEANLNSPSFLNRFVEALFFYSVLF
DCFEDCMDRDNLVRKRIEVFHIGEGIRNMVAAEGAERFTRNVKLDVWRAYFARFGMVEMELSESSRYQANLIL
KQFSHGSSCIVQKDGKALLVGWKGTPIESVSIWKFL-

SlGRAS34 Solyc06g035620.2.1

Nucleotide sequence (ORF):
ATGGAGGACTTGCCCTTGCAGGGTGTTCCGTTTAGCAATCTGAAGAGCTGCTTATATGTTGTGTCCGTTAC
TGGAGATCCCATCCAGCGTCTTGGTGCTTACATTGTAGAAGGCCTCGTAGAAAGAAAGGAGTTATCTGGA
ACCACCATATACAGGAGTTTGAAGTGTAAAGAGCCGGCTGAAGCATGCAGAAATGAAGATCGCATCCAC
ATTATAGACTTCCAAATTGCACAGGGGACCCAATGGATGATTCTCTTACAAGCTCTTGCAGCACGACCCGG
TGGTGCCCTCTACGTACGTATTACAGGAATTGATGATTTAGTTTCACAGTACGCTCGGGGAGATGGATTG
GCTGCAGTTGCCAGACGGCTATCAGCAATTTCTGAGGAGTTCAACATTGCTGTTGCGTTTCATGCAGTGCC
AGTTTTTGCTCTGGAAATCACTTGGGATATGCTTGATGTAAGGCCTGGTGAGGCTCAGGCTGTAAACTTTC
CTTTGCAACTTCACCACACCCCTGATGAGAGCGTTGACGCGAATAACCCTAGAGATGGTCTCATTAGGATG
ATAAAGTCACTTTGCCCCAAGATAGTCACTTTGGTGGAGCAAGAATCAAACACGAACACAGCTCCATTTCT
CACTAGGTTTGTAGAAGCTCTAGATTACTACCATGCAATGTTTGAGTCCATAGATGTGACCCTACTAAGGG
ACATGAAGGAGCGGATCACTGTGGAGCAGCACTATTTGGCTAGGGATATAGTGAATGTCATAGCATGTG
AGGGCAAGGAAAGAGTAGAACGTCACGAGCTATTGGGGAAGTGGAAATCCAGGTTCAAGATGGCAGGT
TTTCAGCAATATCCTTTGAGCTCTTTTGTGAATTCAGTGATTAAGGACCTCATGAAGCGTTACTCGGATCAT
TATACACTGGTGGAGCAAGACGGAGCTATGCTGATATGCTGTGGGGGTGGAAGGAGCGGAATCTAGTCT
CTGCGTCTGCTTGGTTTTAACGAATTACTTCAGAAACGAAGCTGTAGTTCATTATGTGGAAACAGATGGTT
AGCCTTCTTTTTTATTACTTATCCAGACAATAGGATTGATCAAGTAATTATACTCGTGTTATATAGTTTCAAT
TTTCTGAAATTTTAGTATATAAAAATGCTTTATGTTTATTGTTCAAGTTATCCTTGATATTATTCGTTAGAAG

TATAAACAATTAGTTCTCGTCCTT

Amino acid sequence:
MEDLPLQGVPFSNLKSCLYVVSVTGDPIQRLGAYIVEGLVERKELSGTTIYRSLKCKEPAEACRNEDRIHIIDFQIAQ
GTQWMILLQALAARPGGALYVRITGIDDLVSQYARGDGLAAVARRLSAISEEFNIAVAFHAVPVFALEITWDML
DVRPGEAQAVNFPLQLHHTPDESVDANNPRDGLIRMIKSLCPKIVTLVEQESNTNTAPFLTRFVEALDYYHAMF
ESIDVTLLRDMKERITVEQHYLARDIVNVIACEGKERVERHELLGKWKSRFKMAGFQQYPLSSFVNSVIKDLMK
RYSDHYTLVEQDGAMLICCGGGRSGI-

SlGRAS35 Solyc06g076290.1.1

Nucleotide sequence (ORF):
ATGGAGTCCTTAATCAGATCTTTTGCAAAGAGGAGAATATCTGCTGCAGACATATTAAAAGCCTTTCAGGT
GTATGTTACAGCATCCCCATTCAGGATGATGTCCAACATAATCGCTAACAAGTCAATTGGGATGCTGACAA
GGGAAGCAACAAGTATACACATTATTGATTTTGGTATTCTGATTACAGGAATTGATTTTCCACAAGCTGGA
TTCAGACCAGCAGAGAGAGTTGAGGAGACAGGGCGTCGACTAGTATATGAAAACAGTCCAAGAGATGAT
GTGCTAAGATTGATTAAGCAAATAAAGCCTGATGTTTTCCTTCATGGAATTGTAAACGGGGCATATAACTC
AAACTTTCTTTGTAATTCGATTTAA

Amino acid sequence:
MESLIRSFAKRRISAADILKAFQVYVTASPFRMMSNIIANKSIGMLTREATSIHIIDFGILITGIDFPQAGFRPAERVE
ETGRRLVYENSPRDDVLRLIKQIKPDVFLHGIVNGAYNSNFLCNSI-

SlGRAS36 Solyc06g082530.1.1

Nucleotide sequence (ORF):
ATGAATACTAGCTCCCATTTCCCAGGTGATCATAGATCATCTGATGGATTTGAATTTGGTCATTGTTGTGGT
GGTGGAGGTGATAACGATGTTGTCTGCTTTTCGAGCAGACGATCATCGGAGGAAGAGGTTGTTATTGGA
AGTGGTAGTGATCGTACAGTAGTCCAAGGAGATTACTTCGATGGAGTTTTTAAGTATATACATCAGATGCT
TATGGAAGATCAAGAAGATTTAGAAAATAGGCCATGTATGTTTCAAGATTGCATAGCTCTTCAAGCAGCT
GAGAAATCGTTTTATGACGCGTTGAATCCACCTACAATTCATCGCGATTTCGTTGATGATAGTGGTAATCA
ATTTCAAGACGATTACTGCGATGCTGTGAATCATCAGTTTGCTGAGACAACTTTCAATTCTGATTTAGATAA
TGACCAATTTTCGTTTTTTTATAACACTAATACTATCTGTCATGGTGTTGGTGATGGTATTAGTGTTGTCAAT
GGCGATACATCGAATGCTATCAATTATCATCAGAAGGGCGAAAATGAAAACAAAAAGCATACTCGCGATG
AAAATAGTGAGAGTTCAAAAGGGCGAAAATCGAAGCAGTTGGCTAGCAATGGAACAGAGGATGGAAAA
ACAGAGGAGCACTATGATAAGGCGCTTCTCTGCCCGGGTTTGAATCCTTCGTTTTACGAAAATCGATCAAA
ATCAGCAACAGCATGGGATAATGCAGCATGGGATAAACAAAAGAGCTCTCATATAAAGCAGTCTAAACGT
GGAAGGCCTCGCGGAAGCAAAAAAGGCGTTAAGACGAATGAAGTAGTAGATCTTACAAGCCTCCTAACG
CGTTGCGCGGAGGCTGCAGCAAGCTACAACACCAAAACATTCATAGAGGTACTCAATAAAATTAGGGAAC
ATTCTTCACCTTTTGGTGATGCTACTTCAAGATTGGCCTATTGCTTTGCCAATGCCCTGGAAGCTCGTTTTG
CTGGCGGAGATACAACATGGATCACAAGTAAGAAAATATCAGCTGCAGATTTCTTAAAAGCTTATCAGGT
CTACATCACTGCTTGTCCATTCAAGAGGATGTCAAACATATTCGCCAACAAGTCTATCGCGAAGCTAACAA

GTGAATCGTCGAGGGTTCATATAATAGATTTTGGTATTCTATATGGATTTCAATGGCCTTGTATAATACAC
GGGATTTCCCTAAGGCCCGGGGGACCTCCAAAGCTTAAGATCACAGGAATAGATTTCCCTCAGCCAGGTT
TTCGACCAGCGGAGAGAGTTGAGGAGACAGGGCGACGTTTGAAGGAGTATTGCAAGAGATTTGATGTTC
CATTTGAGTATAAAGCAATAGCTAAGAAATGGGATGAGATTAAAGTAGAAGATTTGGATATCGATAGAG
ATGAAATCGTCGTGGTGAATTGTTTGTACAGGCTGAAGAATGTCCTTGATGAAACAGTAGAAGTAACACA
CAACAACAAAAATCCAAGAGATGCTGTTCTCAACTTGATCAAGGAGATAAATCCTCATTACTTTGTCCATG
GGATCGTCAACGCTATGTACAATGCATCTTTCTTTACTACAAGATTTCGAGAGGCATTATTCCATTTCTCGT
CCCAATTTGACATGTTTGAGGCCACAATGCCACGAGAAGATGAAGGAAGGATGATGTTTGAGCAGGAAG
TATTCGGAAGAGATATCATGAACGTTATAGCTAGTGAAGGGGCGGAGAGAGTGGAGAGGCCTGAAAGTT
ACAAGAAATGGTCAATGAGGAATCAGATAGCGGGGTTTAGACAACTTCCATTGGATCAAGACATCGTCAA
GGAAGTAAAAACCAAGGTGAAAATGTTCTATCACGGGGATTTTCTGGTGGATGAAGATAGCAATTGGAT
GTTGCAAGGCTGGAAAGGAAGAATTATGTATGCACTCTCAGTTTGGGAGCCTATTCATAAGTAA

Amino acid sequence:
MNTSSHFPGDHRSSDGFEFGHCCGGGGDNDVVCFSSRRSSEEEVVIGSGSDRTVVQGDYFDGVFKYIHQML
MEDQEDLENRPCMFQDCIALQAAEKSFYDALNPPTIHRDFVDDSGNQFQDDYCDAVNHQFAETTFNSDLDN
DQFSFFYNTNTICHGVGDGISVVNGDTSNAINYHQKGENENKKHTRDENSESSKGRKSKQLASNGTEDGKTEE
HYDKALLCPGLNPSFYENRSKSATAWDNAAWDKQKSSHIKQSKRGRPRGSKKGVKTNEVVDLTSLLTRCAEAAA
SYNTKTFIEVLNKIREHSSPFGDATSRLAYCFANALEARFAGGDTTWITSKKISAADFLKAYQVYITACPFKRMSNIF
ANKSIAKLTSESSRVHIIDFGILYGFQWPCIIHGISLRPGGPPKLKITGIDFPQPGFRPAERVEETGRRLKEYCKRFDV
PFEYKAIAKKWDEIKVEDLDIDRDEIVVVNCLYRLKNVLDETVEVTHNNKNPRDAVLNLIKEINPHYFVHGIVNA
MYNASFFTTRFREALFHFSSQFDMFEATMPREDEGRMMFEQEVFGRDIMNVIASEGAERVERPESYKKWSM
RNQIAGFRQLPLDQDIVKEVKTKVKMFYHGDFLVDEDSNWMLQGWKGRIMYALSVWEPIHK-

SlGRAS37 Solyc07g043330.1.1

Nucleotide sequence (ORF):
ATGCTTTTCGTATCTGCAATTCCAATTAATCATACATCTTCGTATTCATCTTCCATGTCTTCTAAGCGCTCAA
TTTCTGAGTTTACTCCGGTCTCAGATGAACCTCAACTTCTTACTAAACGGCCCCGAAATGAAAGGGGAGAG
GAAGAGGAAGAAGAAGGTGAGGAGTTACTTCTCGTTGACGCCGATTCCATAGGTCTTCGCCTTCTCGGCT
TGCTCCTTCAATGCGCTGAGTTTGTTGCCATGGAGAATCTCGATGAAGCTGCTGATTTATTGCCGGAAATC
GCTGAGCTTTCTTCGCCGTTCGGCTCATCAGCTGAGCGAGTCGCTGCTTACTTCGCTGAATCTCTATCGGCC
AGAATTATCAGTTCTCATCTCCGATTTTATTCTCCGCTTAACCTCAAATCCCTAACTCTCACGCATTCCCAAA
AACTCTTCACCGCTCTGCAATCTTACAACACAATAAGTCCGCTCATCAAATTCTCTCACTACACTGCAAATC
AAGCTATCTACCAAGCATTAGAAGGTGAAGATCACGTCCACGTCATCGATCTCGACATCATGCAAGGTCTT
CAATGGCCAGGATTGTTCCAAATCCTCTCCTCTCGATCGAGAAAGCTCCGTTCCATCAGAATCACCGGCGT
CGGATCCTCCATGGAATTACTCGAATCCACCGGCCGTCGACTCACCGAATTCGCCAACTCATTCGGACTTC
CGTTCGAGTTTCAACCTTTTGAAGGCAAAATCGGACACATCACAGACCTGAATCAACTCGGAGTCAAAATT
GGGGAAACTACCGTTGTCAATTGGATGCACCATTGCCTCTACAATATCACAGGGAGTGATTTAGGTACTTT
CCGATTGTTAACTTTGTTAAGGCCGAAATTGATCACACTGGTTGAACAAGATCTGAGTCACGGAGGAAAC
TTTTTGAGCCGATTCGTTGAGGCATTGCATTATTACTCCGCCTTGTTTGATGCATTGGGGGATGGATTGAG
TGAAGAGAGTGCAGAGAGGCATAGAGTGGAACAGCAGTTGTTCGGTAGCGAAATTAGGAATATTGTAGC
GGTAGGTGGGCCCAAGAGGACGGGGGAAGTACCCGTAGAGAGATGGGGCGATGAATTGAAACGAATC

GGGTTTTTACCCGTTTCATTATCGGGTACTCCAGCTGCTCAAGCAAGTTTATTATTAGGGATGTTTCCGAGA
GGGTATACGTTGGTGGAAGAAAATGGGTGTTTAAAATTGGGATGGAAGGATTTATCGTTGTTGACTGCAT
CTGCATGGCAACCTTGCGATTAA

Amino acid sequence:
MLFVSAIPINHTSSYSSSMSSKRSISEFTPVSDEPQLLTKRPRNERGEEEEEEGEELLLVDADSIGLRLLGLLLQCAE
FVAMENLDEAADLLPEIAELSSPFGSSAERVAAYFAESLSARIISSHLRFYSPLNLKSLTLTHSQKLFTALQSYNTISPL
IKFSHYTANQAIYQALEGEDHVHVIDLDIMQGLQWPGLFQILSSRSRKLRSIRITGVGSSMELLESTGRRLTEFAN
SFGLPFEFQPFEGKIGHITDLNQLGVKIGETTVVNWMHHCLYNITGSDLGTFRLLTLLRPKLITLVEQDLSHGGNF
LSRFVEALHYYSALFDALGDGLSEESAERHRVEQQLFGSEIRNIVAVGGPKRTGEVPVERWGDELKRIGFLPVSLS
GTPAAQASLLLGMFPRGYTLVEENGCLKLGWKDLSLLTASAWQPCD-

SlGRAS38 Solyc07g052960.1.1

Nucleotide sequence (ORF):
ATGAACTCTACAACCATTCCCCTCCAAAACCCTAGTTTTTTCAACAACTCTCAAAGTTCTCTAGCAGGAGCA
CTACAAGGATGCATTGGGAGCCTTGATGGAGCATGTTTGGAGAAACTATTACTTCATTGTGCAAGTGCCTT
AGAGAACAATGATGTAACTTTGGCTCAACAAGTCATGTGGGTGCTCAACAATTTGGCTTCTTCTAATGGAG
ATCCAAATCAAAGACTCACATCATGGTTCCTTAGGGCATTAATTTCAAGGGCTTCTAGGGTTTATCCTAAT
GCCACAAATTTACATGGAAGTAGTAACCTTGAAAGGAGATTAATGAGTGTGACTGAGCTAGCAGGGTAT
GTGGATCTCATCCCATGGCATAGATTTGGATTTTGTGCATCAAATAGTGTTATTTATAAGGCCATTGAAAG
GCAAACAAAAGTTCACATATTAGATTTTAGTATCACTCATTGTATGCAATGGCCAACTCTCATTGATGCAAT
TGCTAAGAGGCCTGAGGGTCCTCCTTCTCTCCGAATATCGGTGCCTTCATGGAGACCACAAGTTCCTCCAT
TGCTCAATGTGTCAAGTGAAGAAGTTGGCCAACGTTTGGCAAATTTCGCGAAATTTAGAGATGTTCCTTTT
GAATTCCATGTGATTGAAGACTTGAACTATGACATGTTCTTGAGCCAATTGAATCCTTCAACTCTTCAAATT
AGAGATGATGAAGCTTTGGTTGTGAATTGCCAAAATTGGTTAAGGTACTTACATGATGATGAACAAATTA
AGGGTGCACTTTCGTCTCGCGACATATTTCTTGATAGGGTTAAGGATTTAAACCCTTGTATTGTGACTATTG
TTGATGAAGATTGTGATTTGGGAAACCCAACTACTTTAACATCAAGAATAGCCACTTGTTTTAACTATCTAT
GGATACCTTTTGATGCATTAGAGACATTTTTGCCTAAGGATAGTAAGCAAAGGCTAGATTATGAAGCTGA
GATTGGCCACAAAATTGAAAACATTATTGGATTTGAAGGGAACCAAAGGATAGAGAGATTAGAGAGTTG
CAACAAGTTCTCACAAAGGATGGAAAATAGTGGTTACATGAGTGTACCTTTTAGTGAAGAAACAATAAAG
GAAGTCAAGTCATTGTTGGATGAACATGCAAGTGGATGGGGCATGAAAAAAGAGGAACATGACATGCTT
GTATTAACATGGAAAGGTCATAACTCTGTCTATGCAACATCTTGGGTCATAGTCTCACCACCCCATATGGA
CATGAGTAGCTTATAA

Amino acid sequence:
MNSTTIPLQNPSFFNNSQSSLAGALQGCIGSLDGACLEKLLLHCASALENNDVTLAQQVMWVLNNLASSNGD
PNQRLTSWFLRALISRASRVYPNATNLHGSSNLERRLMSVTELAGYVDLIPWHRFGFCASNSVIYKAIERQTKVH
ILDFSITHCMQWPTLIDAIAKRPEGPPSLRISVPSWRPQVPPLLNVSSEEVGQRLANFAKFRDVPFEFHVIEDLNY
DMFLSQLNPSTLQIRDDEALVVNCQNWLRYLHDDEQIKGALSSRDIFLDRVKDLNPCIVTIVDEDCDLGNPTTLT
SRIATCFNYLWIPFDALETFLPKDSKQRLDYEAEIGHKIENIIGFEGNQRIERLESCNKFSQRMENSGYMSVPFSEE
TIKEVKSLLDEHASGWGMKKEEHDMLVLTWKGHNSVYATSWVIVSPPHMDMSSL-

SlGRAS39 Solyc08g014030.1.1

Nucleotide sequence (ORF):
ATGCAAAACCCTAATTATCATGAGAATTCATCATCATCAAGAAGTGAATCACCGGAAGGCGTTGAGCCTG
TTTCCGATGCCAAGTGGGCTCCTAAGCTTCTCCATGAGTGTGCTACCGCGATTTCTAATAAGGATTCAACA
AAGATCCATCACTTGTTATGGATGTTGAATGAACTTGCTTCTCCCTATGGTGATTGCAATCAAAAATTGGC
ATTTTATTTCTTACAAGCCCTTTTTTGTAAGGCTACGGATACTGGCTTAAGGTGTTACAAAACCCTAACTTC
AGCAGCTGAAAAAAGCAAATCATTTGATTCATCTAGGAAATTGATCTTAAAGTTTCAAGAGGTAAGTCCAT
GGACAACTTTTGGTCATGTTGCATCAAATGGTGCAATATTAGAAGCATTAGATGGAGAGAATAAGCTTCA
TATAATTGATATAAGTAACACTTTTTGTACACAATGGCCTACTTTGCTTGAAGCCTTAGCTACACGAAACGA
TGAAACGCCACATCTTAAGCTCACGGTTGTGGTCATGGAGAGCACCCTAGTGAAGTCTCATGTTATGAAA
GAAGTAGCACAAAGGATGGAGAAATTTGCTAGGTTAATGGGAGTGCCTTTTGAGTTTAATGTTATAAGTG
GATTGAACCATTTGAAAGAACTCACAAAAGAGAGTTTGAATATAAAAGAAGAGGAAGCATTGGCTGTGA
ATTGCATTGGAGCACTGAGAAGAGTTGAAGTAGATGAAAGGGGAGCTTTTATAAGAATGATTCAATCACT
AAACCCTAAAGTTGTGACAATAGTAGAACATGAGGCTGATTTTTCTAGCACAAGGAATGACTTTGTTAAGT
GCTTTGAAGAATGTCTTAGGTTCTACACATTGTATTTTGAGATGCTTGATGAAAGCTTTCCAACAACAAGC
AACGAAAGGCTAGTGATCGAAAGGGAATGTTCTAAATGCATAGTTAAGGTTTTGGCTTGTGATAATGATA
TTGGTGATGATCAAGGATATAGTGAAAGAAGAGAAAGAGGAAATCAATGGTGTGAAAGACTTAAGGAAT
GTTTTACCCCATTTACACTAAATGATGATGCTGTTGATGATGTTAAGGCTTTGCTCAAGAGGTATAAAGCT
GGATGGTCACTTGTGCTACCACAAGCAAATCAAGAGTCACCTGGAATTTACTTGACATGGAGAGAACAAC
AAGTGGTATGGGCTTCCTCATGGAAACCCCAGGAAAAATCAATGGAGGTAGAGACATGA

Amino acid sequence:
MQNPNYHENSSSSRSESPEGVEPVSDAKWAPKLLHECATAISNKDSTKIHHLLWMLNELASPYGDCNQKLAFY
FLQALFCKATDTGLRCYKTLTSAAEKSKSFDSSRKLILKFQEVSPWTTFGHVASNGAILEALDGENKLHIIDISNTFC
TQWPTLLEALATRNDETPHLKLTVVVMESTLVKSHVMKEVAQRMEKFARLMGVPFEFNVISGLNHLKELTKESL
NIKEEEALAVNCIGALRRVEVDERGAFIRMIQSLNPKVVTIVEHEADFSSTRNDFVKCFEECLRFYTLYFEMLDESF
PTTSNERLVIERECSKCIVKVLACDNDIGDDQGYSERRERGNQWCERLKECFTPFTLNDDAVDDVKALLKRYKA
GWSLVLPQANQESPGIYLTWREQQVVWASSWKPQEKSMEVET-

SlGRAS40 Solyc08g078800.1.1

Nucleotide sequence (ORF):
ATGGCAAACCAAACTCAATTCCAGAATGCGGCTTGTGTTAACATTTTTGGGTCATCATCATATGACATCAA
TCAAGAACAGCCTCCGCCCAAACGCCACAATTCGGGTACCCTTGGAGGAGCTCTATTGCCTAAAGTTCCTT
TTTTTGATGCCAATTGTGACTTCATGTTGAGGAAACAACCATTGGGACAAATGCAGCCACAAGTTAATTTG
CTGCCTCCTCACCAGTTTCAGCCAAAGCCATTGATTGTACCTAAGCTTGAGGCAGCTGGTGGTGGTGGTAA
TGGTAATTTGATGGTGCCTCGTCATCAATTGCAGGAACAACAGTTCATTTATGACCAGATTTTTCAGGCCT
CTGAACTATTGCTGGCTGGACAATTCTCAAACGCGCAAATGATATTGGCGCGGCTCAATCAACAGCTTTCT
CCCATTGGGAAACCCTCAAGGAGGGCTGCTTTTTATATCAAGGAGGCTCTGCAGTTGCCTTTCCTTTTGCC
TTGCACATCCACATTTTTGCCTCCAAGAAGTCCCACCCCATTTGACTGTGTGCTTAAGATGGATGCTTACAA
GGCCTTTTCCGAAATATCTCCACTTATCCAATTCATGAATTTCACCTCCAATCAGGCTATTCTTGAAGCTCTT

GGGGATGCTGAGCAAATTCACATAATAGATTTTGACATAGGTTTTGGTGCTCAATGGTCCTCGTTTATGCA
AGAGCTCCCAAGTAGCAATAGAAAGGCAACTTCTCTAAAGATTACAGCCTTTGCCTCCCCATCAACCCCACC
ACTCAATCGAAATTGGTATCATGCATGAAAGTTTAACACAGTTTGCTAATGACGCGGGAATACGATTTGA
GCTTGAAGTTATTAACTTGGATTCCTTTGACCCCAAATCTTATCCCTTATCCACCTTGAGGTCATCTGAGTG
TGAGGCAATTGCTATAAATTTCCCTATCTGGTCTATTTCAAGCTGTCCGTTTGCATTTCCTTCACTTCTTCAC
TGCATGAAGCAATTATCTCCTAAAGTTGTTGTATCATTGGAACGCGGATGTGAACGTACTGAACTCCCCTT
AAAGCATCACCTCATCCATGCTCTCCAGTATTACGAGACACTCTTAGCTAGTATTGATGCTGCTAATATAAC
TCCAGACATTGGGAAAAAGATAGAGAGGTCTCTCTTCCAGCCTAGCATTGAGAACATGATCTTGGGGCGC
CTCCGATCCTCTGATCGGATGCCACCATGGAGAAACCTTTTTGCTTCTGCAGGATTTTCGCCTGTTGCATTT
AGTAATATGACTGAAATCCAGGCTGAATGTGTTGTTAAGAGAACTCAGGTAGGAGGGTTTCACGTCCAGA
AGCGACAGACGTCACTTGTGCTATGCTGGAAGCAGCAGGAGCTCTTGTCAGCTGTGACTTGGAGGTGCTG
A

Amino acid sequence:
MANQTQFQNAACVNIFGSSSYDINQEQPPPKRHNSGTLGGALLPKVPFFDANCDFMLRKQPLGQMQPQVN
LLPPHQFQPKPLIVPKLEAAGGGGNGNLMVPRHQLQEQQFIYDQIFQASELLLAGQFSNAQMILARLNQQLSP
IGKPSRRAAFYIKEALQLPFLLPCTSTFLPPRSPTPFDCVLKMDAYKAFSEISPLIQFMNFTSNQAILEALGDAEQIH
IIDFDIGFGAQWSSFMQELPSSNRKATSLKITAFASPSTHHSIEIGIMHESLTQFANDAGIRFELEVINLDSFDPKSY
PLSTLRSSECEAIAINFPIWSISSCPFAFPSLLHCMKQLSPKVVVSLERGCERTELPLKHHLIHALQYYETLLASIDAA
NITPDIGKKIERSLFQPSIENMILGRLRSSDRMPPWRNLFASAGFSPVAFSNMTEIQAECVVKRTQVGGFHVQK
RQTSLVLCWKQQELLSAVTWRC-

SlGRAS41 Solyc08g080400.1.1

Nucleotide sequence (ORF):
ATGTTGGCTGGTTGTTCTTCTTCATTGTTGTCACCTAGGCATAGATTAAGGAGTGAAGCATCTGCACAGTT
TCAAGCTTGTAATTTCCCTTCAATGAGCACACAAAGATTGGACTTGCCATGTAGTTTTGCCAGGAAAGATA
CCTCAAGGTCCCAGTCCATTAGTAGGCCAGTGGGACTTTCTGTTGAGAAGCCAATTGAAGCGAAGAACAG
TACTTGCTCTCTCAAGCAGAACATTCGTCTACCGCCATCCTCGACAAGTGCTCAGACATCATATGTAGAAG
GTAGGAGAGAAAGCAAAGATGAATTTTGGGAAAAAGGTAGGAGCTTGAAGAGGTATGCTGAGCAGGGA
TGTATTGATGATGAGAGCTGCATGAGCAGAGCTAAGAGGAAAAAAAGTAATAGGAAGTCAGGTGATTCT
TCAGAAGATGGCTATGATCTGAGTTTGAGCCAATTGGGTGGTGGCGATTTCTGGTTACAATCAGGTTTCA
ATGCGGCGCGATCAGTTCCCCTAATTGCAGGCCTTCATCCACCTCAAGCAGCACCATTCTCCTTTTCTTGTT
CCGGAGAGGAAGAGAGTGTATGTTATGTGCCAAATGAGGTGATATCACCACCTCTGCCTATGTCAAACAA
TCCTTGGATTGAATCTGTAGTGACTGAGATTACTAATTTTGGCGACAAGAATGTCTCAACAAGTCAAGATC
TTGCCAGAGAGGCCTCAGTATCAAGTGCTTCTTTAGACAGTCATGGCTTGGTTCTTAGGCCTAATGAGAAT
CTCGGGGAGCATGAAGTAGGCAATGGTTCTAGGCTACCTAATCCAAATGACAGGGGTGAAGTTGTAACA
GCACATAATGGGCATAATAACCACAGGGAGGATGATGCAGCTGAGCTGATCAGCTTACTTGTGAGTTGTG
TTGAAGCAATTGGCTCAAGGAATGTTACTGGTGTTAATCAACTGATAGCTAGGCTAGGGCAGCTTGCCTC
TCCAAGAGGGTCTCCAGTAAGTCGGCTAACTGCTTACTTCACCGAAGCTTTAGCTTTGCGTGTTGCGAGGA
TTTGGCCTCATATCTTTCACATTATACCCCCTCGGGACCTTGATCGTTTAGATGATGATAGCAGTACAGCAT
TGAGGCTGTTGAATCAGGTTAGTCCAATTCCAAAGTTCATCCATTTCACATCAAATGAGATTCTGCTTAGA
GCTTTTGAAGGCAAGGACCGGGTTCACATTATTGATTTTGACATTAAGCAAGGGCTGCAGTGGCCTAGCC

TGTTTCAGAGTTTGGCTTCTAGGCCCAACCCTCCTACTCATGTTAGAATTACTGGTATAGGAGAATCAAAG
CAGGATCTTGTTGAAACAGGAGATAGACTTGCTGAGTTTGCTGAGGCGTTGAATCTGGCTTTCGAGTTCC
ATCCAGTTGTTGACAGGTTGGAAGATGTACGGCTATGGATGCTTCATGTAAAAGAGGGAGAAAGTGTTG
CAGTGAATTGTGTGTTGCAGATGCATAGGCTTCTATATGATAGTTCTGGGGGGATCCTGAGAGACTTTTTG
GGGCTGATTAGAAGCACAAATCCCACCATTATTCTGATGGCAGAGCAAGAAGCTGAGCACAATGAGCCTA
GCTTGGAAGCAAGACTTGTCAACTCACTTAGATACTATGCTGCTGTATTTGATTCTATTGCTTTCGGTCTTC
CATTAGACAGTCCTGCCAGGATTAAGATAGAGGAGTTGTTTGCTCGGGATATTAGAAATATCATTGCTTGT
GAAGGACGAGATAGGACTGAAAGGCATGAATGCTTTGGGAAATGGCGGAAGTTGATGGAACAAGGGGG
TTTCAGATGCACTGGGATTACAGAAAGGGAACTGCTTCAGAGTCAAATGCTGTTGAAGATGTACTCATGT
GAAGACTACAGGGTCACAAAGCAGGGGAATGATGATGCTGCACTTACTCTGAGTTGGTTAGACCAGCCTC
TATGCACAGTCTCCGCGTGGACACCCATTGATGCTGCTGGAAGTTCATCCTCTTATTATCAGCCAAGTTGA

Amino acid sequence:
MLAGCSSSLLSPRHRLRSEASAQFQACNFPSMSTQRLDLPCSFARKDTSRSQSISRPVGLSVEKPIEAKNSTCSLK
QNIRLPPSSTSAQTSYVEGRRESKDEFWEKGRSLKRYAEQGCIDDESCMSRAKRKKSNRKSGDSSEDGYDLSLS
QLGGGDFWLQSGFNAARSVPLIAGLHPPQAAPFSFSCSGEEESVCYVPNEVISPPLPMSNNPWIESVVTEITNF
GDKNVSTSQDLAREASVSSASLDSHGLVLRPNENLGEHEVGNGSRLPNPNDRGEVVTAHNGHNNHREDDAA
ELISLLVSCVEAIGSRNVTGVNQLIARLGQLASPRGSPVSRLTAYFTEALALRVARIWPHIFHIIPPRDLDRLDDDSS
TALRLLNQVSPIPKFIHFTSNEILLRAFEGKDRVHIIDFDIKQGLQWPSLFQSLASRPNPPTHVRITGIGESKQDLVE
TGDRLAEFAEALNLAFEFHPVVDRLEDVRLWMLHVKEGESVAVNCVLQMHRLLYDSSGGILRDFLGLIRSTNPTI
ILMAEQEAEHNEPSLEARLVNSLRYYAAVFDSIAFGLPLDSPARIKIEELFARDIRNIIACEGRDRTERHECFGKWR
KLMEQGGFRCTGITERELLQSQMLLKMYSCEDYRVTKQGNDDAALTLSWLDQPLCTVSAWTPIDAAGSSSSYY
QPS-

SlGRAS42 Solyc09g010920.1.1

Nucleotide sequence (ORF):
ATGGATCCGAGGTTTAATGGACATCGGAGTTCTTTGAATAATTTTCGATTTGGGAACCAACCATTACCTTTT
TCTTCAGAGCAGAGGATGATTAATGTACCAAGATTTGGACTCATTTATTCAGATCAGAATCTTCTCAATGG
TCCTAGACTTGAAAATAACTTTGTCCAACACGATTTTGGTGCTGTTGGATTTATAAGTGGTGATCATTTACC
TACTAGTGTAGAATCAAGAACTGACCTTGGTGTTGAGAACGACTTCAATGAAGATATTGATTTCTCGGATG
CAGTGTTGAGCTATATAAATCGGATGCTAATGGAAGAAGACATGGAGGACAAGACAGATATGCTTCAGG
AGTCCTTGGAACTTCAAGCTAAAGAGAAGTCACTTTATGAGGCCCTCGGTAAGAAGTATCCACCATCACTG
GAGCAAAACGTGTTTACTGTTCGGAACAGTGAGAGTCCTGATGACTACGTTGCAGGAAGTATATACAATT
CCACAAGTAATACTGGTGACAGTAGTGGTTACCTAGTCGATCCAAGGGGGGTTAACATTTCTACCGACTG
CAATTCTTCTTACTTCGAAGGCCTTTCATTTCATAATACTTCTTCACTATGCTCATCAAATAGTGGGAGTAAT
GTTGTCGATGGATTCTTAGATTCTCCTGTTAGTTCTTTTCGTATTCCTGATATATACGATGAGAGTCGATCT
ATTTTGAACTTTCAGAAAGGAGTTGAAGAAGCAAGTAAGTTCCTCCCGACTAGTAATAAGTTGTTGAACA
GTATAGATATAAATGGCTTGCCATCTCGGGAGCCCCATAGGCAAACTGCTTATGCAGCTGCTCAGGTGGA
GGAAAAGGATGAGGGAGAGACTTCACCAACTGAGGGGAGAGGGAAGAAAAATCCTCTGAGGGGTGATA
ATAATATCGAAGAAGAAAGAAGTAGCAAACAGGCTGCAGTTTTTACTGAATCAACTCTACGATCCGAGGA
GTTTGATATCGTCTTGCTGAATAGCATGGGGAAGGGAGGAGAAGCATTAGAAGCTTATCAACAAAACCTG
AGGAATGCTAAAAGCAAAACTACAGTGCAGATATCAAAAAAATCAAAAGGAGGAAAGGGCCGGGGGAA

GAAACAGGGTGGAAAGAAGGAAGTCATAGATTTGAGAACTCTCATGATAAATTGTGCGCAAGCTGTTGC
TGCTGACGATTCCAGGATTGCGAATGAACTTTTGAAACAAATCAGACAACATTCATCCCCATTTGGAGATG
GAAACCAGAGATTAGCTCATTGCTTTGCAGATGGCCTCGAGGCGCGTTTGGCTGGTACTGGTAGCCAGAT
ATATAAAGCTCTTGTCAATAAACGAACATCTGCAGCTGATCTTTTGAAGGCCTACCATTTGTATCTTGCATC
TAGCCCATTCAGAAAGATCTCAAGTTTTGCTTCAAACAAGACAATCATGCTAAAGGCAGAGAATGCAACA
AGGGTTCATGTTATTGACTTTGGCATCCTGTATGGCTTCCAATGGCCTACATTCATCCAACGTATTGCAGAA
AGAGAAGGGGGGCCACCAAGGCTTCGTATTACTGGCATAGAGTTTCCGCAACCAGGCTTCAGGCCAGCG
GAACGGATTGAAGAAACAGGTCGCCGTTTAGCTGATTATGCTCGATCCTTTAATGTTCCATTTGAATACCA
TGCAATTGCAAAGAAATGGGAGAGCATTACAGTTGAGGATCTAAAACTCGACAAAGATGAATTTCTTGCT
GTCAACTGCTTGTATCGTTTTAAGAACCTGCATGATGAGACTATTGCAGTTGAAAGTTCAAGAACTATCGT
ACTCAACCTCGTAAGGAAGATCAATCCAGATATCTTCGTGCATGGTATTGTCAATGGGGCTTATAGTGCCC
CATTTTTCGTCACACGGTTTCGTGAGGCCTTGTTCCACTTTTCTGCACTTTTTGATATTCTTGAAACTATTGT
TCCCCGTGAGATTCCAGAAAGACGATTAATTGAGAGAGAGATATTTGGGAGGGAAGCCCTGAATGTCAT
AGCTTGTGAGGGGTGGGAAAGAGTTGAACGTCCAGAGACCTACAAGCAGTGGCAAGCTCGTATTATGGG
GGCAAGGTTTACACAGATACCTTTTGACCGGGAGGAATTTGTGAATAAGGCAATAGAAAAAGTGAGGTT
AGGATATCATAGAGACTTTGTAATAGATGAAGATAGCCAGTGGTTGTTGCTGGGGTGGAAAGGAAGAAC
AATTTACGCCTTATCTTGTTGGAAACCTGTGTAA

Amino acid sequence:
MDPRFNGHRSSLNNFRFGNQPLPFSSEQRMINVPRFGLIYSDQNLLNGPRLENNFVQHDFGAVGFISGDHLPT
SVESRTDLGVENDFNEDIDFSDAVLSYINRMLMEEDMEDKTDMLQESLELQAKEKSLYEALGKKYPPSLEQNVF
TVRNSESPDDYVAGSIYNSTSNTGDSSGYLVDPRGVNISTDCNSSYFEGLSFHNTSSLCSSNSGSNVVDGFLDSP
VSSFRIPDIYDESRSILNFQKGVEEASKFLPTSNKLLNSIDINGLPSREPHRQTAYAAAQVEEKDEGETSPTEGRGK
KNPLRGDNNIEEERSSKQAAVFTESTLRSEEFDIVLLNSMGKGGEALEAYQQNLRNAKSKTTVQISKKSKGGKGR
GKKQGGKKEVIDLRTLMINCAQAVAADDSRIANELLKQIRQHSSPFGDGNQRLAHCFADGLEARLAGTGSQIYK
ALVNKRTSAADLLKAYHLYLASSPFRKISSFASNKTIMLKAENATRVHVIDFGILYGFQWPTFIQRIAEREGGPPRL
RITGIEFPQPGFRPAERIEETGRRLADYARSFNVPFEYHAIAKKWESITVEDLKLDKDEFLAVNCLYRFKNLHDETIA
VESSRTIVLNLVRKINPDIFVHGIVNGAYSAPFFVTRFREALFHFSALFDILETIVPREIPERRLIEREIFGREALNVIA
CEGWERVERPETYKQWQARIMGARFTQIPFDREEFVNKAIEKVRLGYHRDFVIDEDSQWLLLGWKGRTIYALS
CWKPV-

SlGRAS43 Solyc09g066450.1.1

Nucleotide sequence (ORF):
ATGATGAAAGGAGAGTCTGAATTAATCCATCAACATACTCCTCTTGATCAACCCAATGAACAATGGGATTA
TTGTTCCCCTAATCATCCCTCCAAGACATTAATCACCGAAAATCGCGCTAATAATCCTGATCAAAGAAATGA
ACTTTCCCAATGGGTTGAGCAAGTCACTAGGCAACTACTCATTGAAGATTTGCCTGAAAATGAATTTAGTG
ATACAGACACAGCGACATATCATGTCTCTGCATTATTAGGAGAGCTAAGGCCTAAGAAAATTGCGCGAAG
AGATTGTAACTCTGAGGGGATTGGAGGACAACAACAACATGAATGGAACTCTAGTACTCATGACGATCAT
CATGTAGCGAGAGATGAGATGGGTGTTAAAGGAATGAGTGGACTAGATGAACAAGGACTAAACTTGATC
ACACTTCTTTTGGAAGGTGCTGTTGCTATATCAGTTGATAATTTGGGAGAAGCTCATAGAGTGTTACTTGA
GTTAACACAAGTGGCATCACCATATGGACCCTCTTGTGCTGAAAGAGTAGTGGCTTATTTTGCCAAAGCAA
TGGCAAGTAGAGTCATCAACTCATGGTTAGGTATATGTTCCCCTTTGATCAATTACAAGACTGTTCATACT

GCTTTACAAGCCTTCAACAACATCTCACCATTCATAAAATTTGCACATTTCACCTCTAACCAAGCAATCCTTG
AGGCATTTCATCGTCGAGACAGAGTCCATATCATTGATGTAGACATCATGCAAGGTCTACAATGGCCGGC
TTTGTTTCACATCCTCGCCACTCGTATGGAAGGGCCTCCACATGTTACAATGACAGGAGTAGGTACATCAA
TGGAACTATTGATTGAGACAGGAAAACAACTTTCCAATTTCGCCAAACGCCTTGGTATGTCATTTGAGTTC
CATCCAGTTGTGGGGAAAACAGGGGAAATCGACATATCAACGTTCAAAATTAGCAGGGGAGAGGCTATA
GCCATACATTGGGTGCAACACTCTTTGTATGATGCCACTGGCCCTGATTGGAAAACAATGAGACTCCTCCA
ACAACTATCACCAAGAGTTGTGACACTAGTAGAACAAGAAATAGCACTAGGGGGTAGTTTCTTGGACCGG
TTCGTGGGATCCCTCCATTACTACTCAACCATATTCGACTCGTTAGGAGCCTTCTTGGAGAGTGATGATTCA
AGCAGGCATAGTGTCGAACACGGCCTACTTTACAGAGAAATCAATAACATATTAGCAATTGGAGGGCCAG
CAAGAAATGGGGAGGATAAGTTTAGGCATTGGAGGAGTGAACTATCAAAAAATGGATTCATTCAAGTGC
CAATGAGTACAAATTCAATGGCACAAGCTCAATTGATACTAAATATGTTCCCACCAGCTCATGGTTATAGC
CTAGTACAAGGTGATGGAACATTAAGGCTAGGGTGGAAGGATACTAGTTTGTTTACTGCATCTGCATGGA
CCTCTCCAAATTCTAGATAA

Amino acid sequence:
MMKGESELIHQHTPLDQPNEQWDYCSPNHPSKTLITENRANNPDQRNELSQWVEQVTRQLLIEDLPENEFSD
TDTATYHVSALLGELRPKKIARRDCNSEGIGGQQQHEWNSSTHDDHHVARDEMGVKGMSGLDEQGLNLITLL
LEGAVAISVDNLGEAHRVLLELTQVASPYGPSCAERVVAYFAKAMASRVINSWLGICSPLINYKTVHTALQAFNNI
SPFIKFAHFTSNQAILEAFHRRDRVHIIDVDIMQGLQWPALFHILATRMEGPPHVTMTGVGTSMELLIETGKQLS
NFAKRLGMSFEFHPVVGKTGEIDISTFKISRGEAIAIHWVQHSLYDATGPDWKTMRLLQQLSPRVVTLVEQEIAL
GGSFLDRFVGSLHYYSTIFDSLGAFLESDDSSRHSVEHGLLYREINNILAIGGPARNGEDKFRHWRSELSKNGFIQ
VPMSTNSMAQAQLILNMFPPAHGYSLVQGDGTLRLGWKDTSLFTASAWTSPNSR-

SlGRAS44 Solyc10g086370.1.1

Nucleotide sequence (ORF):
ATGAGTTCACGAGAGATATCCATGGATACAATTATGGAAGATGAAGATTCCTTTGTTTCTAATACAAATGC
GATCATCTACGCTGCTTCAGATATCTCAGGCTGGACCCACAGTTTGTTCTCAGATAACAATATTCCTAATTC
ATCCTCTTCTACCAGTGTAGACGAGCACCATCAGCAGATTTCAACTGCTGCCGGTGACGGTGGTAAAGAG
GATGATTCGATGATTGTATCATCAAGTGCATCTTCCAGCTGGTTTAATGATAATAAGCAAATTGATGATCA
CCAAGAGATTAGGATATTTAACGTTGATTTCAGTTCACTTTGCAACAACACTTGCAGTGGATTTGAATCTC
ATGGTGGTTTATTGCCGGATACTGGTCGTACAGTGAACGGAGACGCTAGTGTTTTGCTTGTCAATATCATA
GTAGCTTGTGCCAAGGCAGACAAAAAGAACTACTCATGTCTAGCGGACAGATTAATTCGTAACATAATAA
GGAGATTTGAAGTTGCACAATTTGGAGGAGCAATGAAGAAAGTGGCGACACATTTTGCAGATGCTTTCGA
TAATAAGATTCACAGATTGATTCCACAAGATATTGTTGGATTATCCTATAACCATACGAGTGTCTACGGGA
ACTGCCTCTTCCTCAAATTTGCCCACTTCACTGCCAATCAATCAATTTTGGAGGCCTTTGCCAATTTCAATAG
AGTACATGTCATTGATTTCAGCTTCAATCAAGGTTTGCAATGGCAGGCACTTTTGCAGGCTCTTGCTTTGC
GTCCTGGCGGTCCACCAGCTTTTCGGCTCTCAGGAATTAGTGGCCAACCTAAGTCTGATGATGGTAGCGA
TCCGTTACAGGAAGTTGGTTTTAAGCTCGCCCAATTTGCAGAGTCAATTGGTGTCGAATTTGAATTCTGTG
GATTTATGGCCTATGCTTTAGCTGATCTTGAAGCATCGATGTTGAACATTAGACCTAGTAATGAGGAAGCA
GTGGCTGTGAACTCTGTTTTCGAGCTTCAACCTCTGTTTTCCATTCCAGGGGCAATTGAGAAAGTGATGGA
TTTAATAAAACAGATAGAGCCCAAGGTTGTGACCATCACCGAACAAGAAGTGAATCACAATGGGAGAGT
CTTTAGTAGCAGGATTATTGAAGCATCACGTTACTATTCAACAATGTTTGATTTTCTGGAGAATTTAGAATC

AATTAGTCCCAACAATTTAGACACGGTCATGGAAAAGCAGTGCCTAGATGTAATGGTGTCAGAGATCTAT
AATTTGGTGGCTTGCGAAGGGACTAAGCGAATTGTGAGACGTGGGACTCTGGGTCAATGGCAAGTGAGG
ATCTACTCTGCAGGGTTCAACCTGGTTCCCCTGACTTCAGATACTTACAAACAGGGAGCTATGTTATTGGC
CTCTTTCCCAAATGCAGAGGGATATAAAGTAGAAGTGAAAGATGGGTCTCTTATGCTGAGCTGGAATAGT
CTCCCTCTCATAGCCAACTCCACTTGGCGGCTATGTAGCCAAGTCTAG

Amino acid sequence:
MSSREISMDTIMEDEDSFVSNTNAIIYAASDISGWTHSLFSDNNIPNSSSSTSVDEHHQQISTAAGDGGKEDDS
MIVSSSASSSWFNDNKQIDDHQEIRIFNVDFSSLCNNTCSGFESHGGLLPDTGRTVNGDASVLLVNIIVACAKAD
KKNYSCLADRLIRNIIRRFEVAQFGGAMKKVATHFADAFDNKIHRLIPQDIVGLSYNHTSVYGNCLFLKFAHFTAN
QSILEAFANFNRVHVIDFSFNQGLQWQALLQALALRPGGPPAFRLSGISGQPKSDDGSDPLQEVGFKLAQFAES
IGVEFEFCGFMAYALADLEASMLNIRPSNEEAVAVNSVFELQPLFSIPGAIEKVMDLIKQIEPKVVTITEQEVNHN
GRVFSSRIIEASRYYSTMFDFLENLESISPNNLDTVMEKQCLDVMVSEIYNLVACEGTKRIVRRGTLGQWQVRIYS
AGFNLVPLTSDTYKQGAMLLASFPNAEGYKVEVKDGSLMLSWNSLPLIANSTWRLCSQV-

SlGRAS45 Solyc10g086380.1.1

Nucleotide sequence (ORF):
ATGAGTTCACTTGAGATATCCATGGATACAATTATGGAAGATGAAGATTCCTTTGTTTCTAATACAGAAGC
AATCATCTACGCTGCTTCTGATATTTCAGGCTGGACTCACAGTTTGATCTCTGATCACAATATTCCTAATTC
GTCGTCTTCTACCAGTTTAGACGAACACCACCACCATCACCAGCAGATTTCGACTGCTGCTGGTGGCGGTG
GTAATGAGGATGATTCAATGATTGTATCATCATGTGCTTCTTCCAGATGGTTTAATAATAATAAGCAAATT
GATGATCAGCAAGAGATTAGGATATTTAATGTTGATCTCAGGGCACTTTGTGAAAACAGACTGAAATCTT
GTGGTGGATCTGAATCTCGTGACTGTTTAGTATACGAAGAGAGTAGTGTTAGGCTTGTCAATACGTTGAT
GGCTTGTGCCGAGGCGATCCAAGATAACAACTTAAGTCTAGCGGATGAACTAATCAGTGATATAAGAAGA
ATTTCAGTTTCACAAATGGGAGGAGCAATGAAGAAAGAGGCTACTTATTTTGCAGACGCTTTGTATCATAA
GATTCACAGAACAAATTCGGAAGATATTGATGAATCATCCTATCCCAAGGATCAAGTCTTGAGTATGAGCT
TCTACGATAGCTGCCTCTTCCTCAAATTCGCTCACTTCATTGCCAATCAATCCATTTTGGAGGCCTTTGCAG
ATTCCAAGAGAGTACATGTAATTGATTTCAGCTTAAATCAAGGTTCACAATGGCCAGCACTTTTACAGGCT
CTCACTTTGCGTCCTGGTGGTCCACCGGCTCTTCGGCTCACAGGAATTCGTGGCCACTCTCAGCCTGAGGA
CACTACCGATGCCTTACAAGAAGTTGGTCGGGAGCTCGCCCAACTTGCAGAGTCAACAGGTGTAGAATTT
GAGTTTCGTGGATTCGTGGTCCATACTTTAGCTGATCTTGAAGCACCAATGTTGAATATTAGACCCAGTAA
TGTGGAATCAGTGGCCGTGAACTCTGTTTTCAAGCTTCACCGTCTGTTTTCCATTCCAGGAGCAATTGAGA
AAGTGCTGGATTTGATAAAACAAATAGACCCGAAGATTGTGACCATTGCTGAACGTGAAGTGAATCACAA
TGAGACTGTTTTTATGAACAGGATTAAGGAAGCATGGTATTACTATTCAACAATGTTTGATTTGCTGGAGA
ACTCAGAGTGGACTAAGCGCAGCACTATAGACTTGGAGATTGCGGCAGAGCACCTAGGGAGGGAGATCT
ATAATTTGGTGGCTTGTGAAGGGACTAAGCGAGTAGTGAGACACGAGACGTTTGGTCAATGGCGAGTGA
GGTTTAATTCTGCAGGGTTCAACCTGGTTCCCCTGGGTTCAAATACGTACAGACACGCAAACATGTTGTTG
GCCTTGTACACAAATGGAAAAAGATATAGAGTAGAAGAGAAAGATGGGTGTCTTATGTTGAGTTGGCAT
AGTCGCCCTCTCATCACCACATCTGCTTGGCGTGGCTATGTCGCCAATTCTAGGGATTAG

Amino acid sequence:
MSSLEISMDTIMEDEDSFVSNTEAIIYAASDISGWTHSLISDHNIPNSSSSTSLDEHHHHHQQISTAAGGGGNED

DSMIVSSCASSRWFNNNKQIDDQQEIRIFNVDLRALCENRLKSCGGSESRDCLVYEESSVRLVNTLMACAEAIQ
DNNLSLADELISDIRRISVSQMGGAMKKEATYFADALYHKIHRTNSEDIDESSYPKDQVLSMSFYDSCLFLKFAHF
IANQSILEAFADSKRVHVIDFSLNQGSQWPALLQALTLRPGGPPALRLTGIRGHSQPEDTTDALQEVGRELAQLA
ESTGVEFEFRGFVVHTLADLEAPMLNIRPSNVESVAVNSVFKLHRLFSIPGAIEKVLDLIKQIDPKIVTIAEREVNH
NETVFMNRIKEAWYYYSTMFDLLENSEWTKRSTIDLEIAAEHLGREIYNLVACEGTKRVVRHETFGQWRVRFNS
AGFNLVPLGSNTYRHANMLLALYTNGKRYRVEEKDGCLMLSWHSRPLITTSAWRGYVANSRD-

SlGRAS46 Solyc10g086530.1.1

Nucleotide sequence (ORF):
ATGGATCCAAGGTTCAATAGATTTCCAACAACTGTAAATGGATTCAATCTGGAGAACCAATCCTTACTAAG
CTTTTCGGATAATTGGAAGAATAATGAACCTATAATTGGGGCTATTTATCCAGATCAGAAAATAGTCAATG
CTCCAACATTTGAAAATAGTTTTATCCAACAGACTGTTCCATCTCTTTCAAATGACCCTTCCACAACAAGCA
ATATAGCATTGACTTCTGAGATGGGTACTGAGGATGAATACAATGAAGATTTTGATTTCTCCGATACGGTT
TTGAGCTATATAAATCAGATGCTCATGGAAGAAGATATGGAGGACAAGACACATATGCTTCACGAGTCGT
TGGAACTTCAAGCAAAAGAGAGATCATTTTATGAGGCCCTTGGCAAGAAGTATCCGCCTTCACCTCACCA
GAACCTGGCCATTACTGAACAGAACGGTGAGATACCGGAGGACTATTACTCAGCAAGTCTGTATAGCTCT
ACAAGTAATGTCATTGACACTAGTGGTTACCTAATTGATCCAAGCATTTCAAATGATCATAATTACTCTTAC
GAACAAGGCCTTTTTATTTGTAATGGTCCTTACTCTTCGATTAGCTCATCGAATAGCATAAATAATCTTGGA
GATGGGTTCTTGGACTCTCCTGTAAGTCCTCTTCATATACCTGATATATACAATGATAGTCACCCAATTTGG
AAATTTAGGAAAGGAGTGGAAGAAGCGAGCAAATTCCTACCTACTTATAGTAAGTTGTTGGACAATGTGG
TTATCAACGACTTGCTACCTCAGGCGAAAAGAGGAGAAAGTGGTTGTGCGGCAGCTCAAGTGGAGAAAC
GGGATGTGGGAGCGACTTCACCTACCGGACCGAGAGGGACGAAAAATCCTCATAGAGATGATAGGGATT
CAGAAGAAGAAGAAGAAAGAAGAAGCAAGCAAGCTGCAGTTTATGCAGAATCTACAGTTCGCTCAGATG
AGTTTGATGTAATTTTGTTGCATAGCATGGGGGATGGAAGGGAAGCATTGACAGCTTATCGTGAGAGCTT
GAAGAACGCTAGAACAAAACCTACAGTGCAATCAAAAGGATTTGCTGTAGGGAGAGGAGGCCGCGGGA
AGAAACAGTCTGGTAAAAAGGAAGTCATAGATTTAAGGTCTCTTTTAATAAATTGTGCACAGGCTGTTGCT
GCTGATGACTGCAGGAGTGCAACTGAACTGCTGAAACAGGTTAGACTCCATTCATCTCCTTTTGGAGATG
GCAACCAGAGATTAGCTCATTGTTTTGCAGATGGTCTGGAAGCACGTTTGGCAGGCACTGGTAGCCAGAT
TTATAAGGCCCTCGTCAATAAACGAACATCAGCAGCTGATTTTTTAAAGGCCTATCATTTGTATCTTGCATC
ATGCCCATTCAGAAAGATTTCAGGTTTTACCTCAAACAAGACGATCATTAGAAAATCAAAGCATGCTACAA
GGGTCCACATCATTGACTTTGGCATCCTCTATGGCTTTCAATGGCCTACGCTTATTCAACGTATTGCAGCAA
GAGAAGGTGGACCACCAAATCTTCGTATAACTGGTATAGAATTTCCTCAACCTGGCTTCAGGCCAGCAGA
AAGGATTGAGGAAACAGGACGCCGTTTGAGCGATTATGCCAAGTCCTTTAATGTTCCATTTGAGTACCAA
GCAATAGCAAAGAAATGGGAAACCATCAGAGTTGAGGATCTAAAGCTTGAAAAGGATGAGTATCTTGTT
GTCAATTGTTTGTATAGATTTAAGAACTTGCACGATGAGACCGTGTTATCTGACAGTTCGAGAACTCTCGT
TCTCAATCTAATAAGGGAGATCAATCCAGACATTTTCATCCATGGGATTGTAAATGGGGCCTATAGTGCCC
CATTCTTTGTCACACGGTTCCGTGAGGTCCTGTTTCATTTTTCAGCACTTTTTGATATGCTGGAAGCTAATG
TGCCCCGGGAGTTTCCGGAAAGACTGTTAATTGAGAGAGAGATCTTTGGGAGGGAAGCCCTCAATGTCAT
AGCTTGTGAGGGATGGGAAAGAGTTGAAAGGCCAGAGACATACAAGCAGTGGCAAGTTCGTCATCTAAG
GGCAAGGTTTACACAAACACCTTTTGAGCAAGAGGAGATCATGAATATGGCAGTTAAAAAGGTGAGAAC
AAGCTATCACAAAGACTTTATAATCGATCAAGATAACAAGTGGATGTTGCTGGGGTGGAAAGGGAGAAC
GATCTACGCCTTATCTTGTTGGACACCTATTTAA

Amino acid sequence:
MDPRFNRFPTTVNGFNLENQSLLSFSDNWKNNEPIIGAIYPDQKIVNAPTFENSFIQQTVPSLSNDPSTTSNIAL
TSEMGTEDEYNEDFDFSDTVLSYINQMLMEEDMEDKTHMLHESLELQAKERSFYEALGKKYPPSPHQNLAITE
QNGEIPEDYYSASLYSSTSNVIDTSGYLIDPSISNDHNYSYEQGLFICNGPYSSISSSNSINNLGDGFLDSPVSPLHIP
DIYNDSHPIWKFRKGVEEASKFLPTYSKLLDNVVINDLLPQAKRGESGCAAAQVEKRDVGATSPTGPRGTKNPH
RDDRDSEEEEERRSKQAAVYAESTVRSDEFDVILLHSMGDGREALTAYRESLKNARTKPTVQSKGFAVGRGGRG
KKQSGKKEVIDLRSLLINCAQAVAADDCRSATELLKQVRLHSSPFGDGNQRLAHCFADGLEARLAGTGSQIYKAL
VNKRTSAADFLKAYHLYLASCPFRKISGFTSNKTIIRKSKHATRVHIIDFGILYGFQWPTLIQRIAAREGGPPNLRIT
GIEFPQPGFRPAERIEETGRRLSDYAKSFNVPFEYQAIAKKWETIRVEDLKLEKDEYLVVNCLYRFKNLHDETVLSD
SSRTLVLNLIREINPDIFIHGIVNGAYSAPFFVTRFREVLFHFSALFDMLEANVPREFPERLLIEREIFGREALNVIAC
EGWERVERPETYKQWQVRHLRARFTQTPFEQEEIMNMAVKKVRTSYHKDFIIDQDNKWMLLGWKGRTIYAL
SCWTPI-

SlGRAS47 Solyc11g005610.1.1

Nucleotide sequence (ORF):
ATGGAGTGTGAAAATTTTCAACCAAATTACATTAATCCATATATTGAGTATAGCAACTTAGAGACCACATT
TTCTTGGAATAATCCTAGTATTATTAACCCTCAATGTTCAATCCAAGATTTTGTAACTATTGTTAATGATGTT
GACATACTAGACTATTTCCCAAATGACTTCGATTTCATCGATGGAGATGTAGACATCCCCCTTCAAAACAC
GCAACAAAAAATTATTCAGGGAGATACTGAACTCTCGAGAGAGGGTCAAAAATCACGTGTCTGCAATCCT
CGTCTCAAATTCCTTCAAGATCACCTCATGGAGGAAACAAGTGTAACTGATCTTCTTCTTATGGGAGCTGA
GGCTATTGAAGCTAGAAATCTTGATCTTGCTTCTATAATTGTTTTGAGGCTAAATACAATACTTCCTAATCA
AGAAAACAGAGAAAATTCCCCTGTTGAAAGATTGGCTTTGTATTTTACACAAGCCTTGTTATGCAAGACCT
TGAATAATTCATCACATGAATTGTTGAATCTAAATCAATATCAATCAGAGTTCACATCTTCAATGACCGCGT
TTCAAATGCTTCAAGAAATCTCTCCTTATGTTAAGTTTGCTCATTTCACCGCGAATCAAGCAATTCTTGAAG
CAACAAAAGGGAGTCAACAAGTTCATATATTGGATTTTGACATCATTGAAGGCATTCAATGGCCTCCTTTG
ATGGTTGATTTGGTTGAAAGAGGAAATACAAATTCTTCTCTAAGGATCACATCACTTGTTAGTGATCATTC
AAACTCATGTCATGTAGAAAAAACAGGCCAAAGACTTCAAGAATTTGCTAATTCAATCAATCTTCCTTTCAT
GTTTGATCAAATCCTCTTAGAGGACTTAGAAAAGTTACAAGTTGTTGTTGAGGGTCATAACAACTTAATAG
CTAATGTTATGATCCACCAACTTCACATGCCACAAAGGGGAAGTTCACTAGTCAAGACATTTTTCAATGGA
CTAAGAAAATTGTCCCCTAAACTTGTTGTCTTAGTTGAAGAAGAGCTCTTCAATTTATCAAAAATCTCATCT
ATGCCATTTGTTGAGTTCTTTTGTGAGGCTTTACATCATTACACAACAATATATGATTCAATTCTTGGAGGT
TTTGGTGGAGGATACAAGTTAGCATTAAGGGTCATTGAGAAGGAATTTTTAAGAGTTAGAATTTTGGATT
CATTAAGACAATTCCCTAGTGATAAATTAGAAAGGGAAAGATGGAGTAAAGGGCTATATTCATTAAAAGG
TTTTAGACAAATTCCAATGAGTTCAAGTAATGTGAGACAAGCCAAGCATTTGGTGAGTTTGTTTAGTGGA
GGGTATTGGGTGCAAAATGAACAAAGCAAATTGGCTTTGTGTTGGAAATCAAGGCCTTTGACAAGTGCTT
CCATTTGGGTTCCTATATCATCAACTTCTAGTCCTTCAAGCTCAATATCTTTTTAA

Amino acid sequence:
MECENFQPNYINPYIEYSNLETTFSWNNPSIINPQCSIQDFVTIVNDVDILDYFPNDFDFIDGDVDIPLQNTQQKI
IQGDTELSREGQKSRVCNPRLKFLQDHLMEETSVTDLLLMGAEAIEARNLDLASIIVLRLNTILPNQENRENSPVE
RLALYFTQALLCKTLNNSSHELLNLNQYQSEFTSSMTAFQMLQEISPYVKFAHFTANQAILEATKGSQQVHILDFD

IIEGIQWPPLMVDLVERGNTNSSLRITSLVSDHSNSCHVEKTGQRLQEFANSINLPFMFDQILLEDLEKLQVVVE
GHNNLIANVMIHQLHMPQRGSSLVKTFFNGLRKLSPKLVVLVEEELFNLSKISSMPFVEFFCEALHHYTTIYDSIL
GGFGGGYKLALRVIEKEFLRVRILDSLRQFPSDKLERERWSKGLYSLKGFRQIPMSSSNVRQAKHLVSLFSGGYW
VQNEQSKLALCWKSRPLTSASIWVPISSTSSPSSSISF-

SlGRAS48 Solyc11g013150.1.1

Nucleotide sequence (ORF):
ATGGCTATGATGGTTGATGAAACCATCACGGATTTTGATTGTTATGGTAGTTTAAGCATCGCCACTACAAC
TATCACTACTACTACAGCAACAACCTCTTCATCTTCATGGAATGACTGGTCGCCGGTTTTTGATTGGGATGT
GTTTTCCGGTGGTGATAATTTTCAAGATCTAATTGAATCTATGGTGCAGGGATCGGGTAATTGCTTTTACA
AATCCGAACATCAAGATTGTGCTGAGTTTATGGAGGAAGAAGAAGAATCAGGTGACTCCAATAACAATCA
ATCGGAAGATATTAACGGGCTGAAGCTTGTTCACCTTCTCATGGCAGCAGCGGAGGCATTAACCGGCGTT
AACAAAAGCCGTGAGTTGGCTCGAGTGATATTGGTTCGGCTCAAGGAATTAGCGTCCCCAAACGGCGCTA
CTAATATGGAGAGATTGGCTGCGTATTTCACTGACGCCTTGCAGGCGTTGATGGCTTCCGGTACGTTACAC
ACTAAACACATGTTTTCCCCAAATTACAAAGACGAACACCGTCAGGGAGATGTACTTGCAGCTTTTCAGCT
ATTACAGGATATGTCTCCTTTTGTTAAATTTGGGCATTTTACAGCAAATCAAGCTATATTCGAATCGGTAAC
ACACGATCGGAGAGTTCATATTATCGATTACGATATAATGGAAGGAATTCAATGGGCATCGTTAATGCAA
GCTTTGGTGTCTAGAAAAGATGGACCTCCAACTCCGCATCTTCGGATCACAGCATTGTCAAGAAGCGGCA
GCGGCCGTCGGTCGTTCGGGACGGTCCAAGAGACCGGCCGTCGGTTAACTGCTTTCGCTGCTTCTATCGG
TCAGCCATTTTCATTTCATCACTGCAGATTGGACTCAGACGAAATATTTAAGCCAACCAATGTCAAGTTAGT
TAGAGGCGAAGCTCTAATTATTAATTGCATGCTTCACTTGCCTCATTTCAGCTATCGAGCCTCGGATTCAGT
CGCTTCGTTCCTATCTGGATCGAAGACACTGAATCCGAGACTTGTGACCTTAGTCGAAGAGGAACTAAGG
CCGATAGGAGACGAAGAAGGGTTCGTGGGTCGGTTCATGGACACTTTACATCACTACTCAGCACTTTATG
ATTCGCTTGAGGCAGGATTCCCTTTGCAAAGCCGAGCTCGGGCATTGGTGGAGCGGGTATTCCTCGGGCC
CAGAATAACCGGGTCATTGGCTCGGATATACCGGGCCCGAGGAGAAGGGGAAAGGTGCTCATGGGGGG
AGTGGTTGAGTGGGGTGGGGTTTTGTGAGAGTAATATAAGCTTTGCAAATCATTGTCAAGCAAAATTGTT
GTTGGGGCTATTCAATGATGGGTATAGGATGGAGGAAATTGGGACCAATAAACTGGTTTTAGGATGGAA
GTCCCGGCGTCTACTTTCTGCTTCTATTTGGACCACAATGGATATTGACTTGTAA

Amino acid sequence:
MAMMVDETITDFDCYGSLSIATTTITTTTATTSSSSWNDWSPVFDWDVFSGGDNFQDLIESMVQGSGNCFYK
SEHQDCAEFMEEEEESGDSNNNQSEDINGLKLVHLLMAAAEALTGVNKSRELARVILVRLKELASPNGATNME
RLAAYFTDALQALMASGTLHTKHMFSPNYKDEHRQGDVLAAFQLLQDMSPFVKFGHFTANQAIFESVTHDRR
VHIIDYDIMEGIQWASLMQALVSRKDGPPTPHLRITALSRSGSGRRSFGTVQETGRRLTAFAASIGQPFSFHHCR
LDSDEIFKPTNVKLVRGEALIINCMLHLPHFSYRASDSVASFLSGSKTLNPRLVTLVEEELRPIGDEEGFVGRFMDT
LHHYSALYDSLEAGFPLQSRARALVERVFLGPRITGSLARIYRARGEGERCSWGEWLSGVGFCESNISFANHCQA
KLLLGLFNDGYRMEEIGTNKLVLGWKSRRLLSASIWTTMDIDL-

SlGRAS49 Solyc11g017100.1.1

Nucleotide sequence (ORF):

ATGGGAGATATTGTAGATCATGAAGAAGAGTTGTTGAGTCTTAGACTTGCTATAGTCAATGATTCATCATG
TTGTGATAATCATATCAATAAGAAGATCATCAAGAAGAGAAAAAGGAGGGAATTAATGATGAATTTCATC
AATACTTGGGATTTAAATGAAAGTAATTGTGAAGGAAAAATATTAAGTCTTTTAGAGTTGAGAGAAATGA
TGTTAAAAAATGATGTAAAGAAAAAAGGTCATATTATTAATGATGGAAAAGGTCTACACCTAATCCATTCA
TTGCTTATTTCAGCCACATCTATTGATGAAAATAGCATGAATTTAGCTATTGAAAATCTACATGAATTGTAC
CAAAATGTTACCTTATTTGGTGATTCAATCCAAAGAATGGCTGCCTATTTTGCTGATGGATTAATAGCAAG
ACTATTAACGCGAAAATCGCCATTTTATGACATGATAATGAAGCCACCAACACAAGAAGAAGAGTTCTTG
GCTTTCACTCAATTCTACAAGGTTTCTCCTTTTTATCAATTTGCTCATTTCACATCTAACCAAGCCATTCTTGA
AGTCTTTGAGAAAGAATTAGAATATAACAATGGATTATTGCATGTTATAGACTTTTTTGACATATCTTATGG
ATTTCAATGGCCTTCTCTAATTCAATCTCTATCAGAAAACTCAACCACATTGAATAGAATTTCTCTCAAGATC
ACTGGATATGGAAGAACTATCGATGAATTGAGAGAAACTGAGACAAGACTTGTTAGTTTTGCAAAGGGTT
TTCGAAATTTATCTTTTGAGTTTCAAGGGGTTTTAAGTGGATATAAACTGAGTAACCTAACAAAAAGGAAA
AATGAAACATTAGCTATAAATTTGATCTTTCATTTGAACTCATTAAGTACTTATTCCAAGATTTCAAAGACA
TTGAAAGAAGTACATGATCTTTGTCCTTCTATAGTTACTATAGTGGAACAAGAAGGATGTAAAATCCCTAA
AACAAATTTCATGCCAAGATTCATGGATTCATTGCATTATTTCGCGGCTATGTTTGATTCATTAGATGATTG
TTTACCTATTGATAGTATTGAAAGATTGAGTATTGAAAAAAACCATCTTGGGAAAGAGATCAAGAATGTG
TTGAATTATGATAATTCATCATCAAGATATAATGATGATAATGAACAAATGGAGACATGTAAAGGGAGAA
TGGAAAGTCATGGATTTATAGGAGTTCCATTGAGTTGTAAGAACATAATGCAAGCAAAGTTGCTTATGAA
AATTAGAAGTTATTCTAATTCTACAATACAAATTGATGGAGGAATAAATGGAGGGTTTAGGGTTTTTGAAA
TTGAGGATCCAAGAGCTATTTCTCTAGCATGGCAAGATAGGTCTATTGTAACTGCATCTGCATGGCATTGT
GTATTGTAA

Amino acid sequence:
MGDIVDHEEELLSLRLAIVNDSSCCDNHINKKIIKKRKRRELMMNFINTWDLNESNCEGKILSLLELREMMLKN
DVKKKGHIINDGKGLHLIHSLLISATSIDENSMNLAIENLHELYQNVTLFGDSIQRMAAYFADGLIARLLTRKSPFY
DMIMKPPTQEEEFLAFTQFYKVSPFYQFAHFTSNQAILEVFEKELEYNNGLLHVIDFFDISYGFQWPSLIQSLSEN
STTLNRISLKITGYGRTIDELRETETRLVSFAKGFRNLSFEFQGVLSGYKLSNLTKRKNETLAINLIFHLNSLSTYSKISK
TLKEVHDLCPSIVTIVEQEGCKIPKTNFMPRFMDSLHYFAAMFDSLDDCLPIDSIERLSIEKNHLGKEIKNVLNYD
NSSSRYNDDNEQMETCKGRMESHGFIGVPLSCKNIMQAKLLMKIRSYSNSTIQIDGGINGGFRVFEIEDPRAISL
AWQDRSIVTASAWHCVL-

SlGRAS50 Solyc12g049320.1.1

Nucleotide sequence (ORF):
ATGACCATTGATGAAACTACTTCTGATCCCATCTCAGAGTGGCTGGATAGTCCCTTATCATACTTTCCGTCGTT
ACTCGATGATGAGTCATATAGCATCGATGATTTACTCGATGAGTCATATAGCCTCGATGATATTTACGAGGACT
CATGGTGGGCTCCTAATGAGAGCATAAACCAAGAAATACATATCAATAACAATAACGATAATAGTTTACCGTG
CTTCGATACTAGCTTCCCTGTCACAACCGCGAGCACTGTCTCTCTGGAGCCGGTCATTTCAAGTCATCTTCCG
CCATTGGATTCATCCAAGAAGAGGAAAGGGAATGATTCTGGTTACAATCCCAAGGCGTCCCGGAAGAATCA
GAACCGTAGGATCAATGATGCAGATACAAGAAGAACAACAGGACACAAGAAAGCAACACACAAGTCTAGT
ACAGGAAATAATGGTAACAACAGAGAAGGAAGATGGGCAGAGCAGTTACTTAACCCTTGTGCTGCTGCAAT
TACAGCAGGAAATCTGAACCGTGTGCAGCACTTGTTGTACGTTCTGCGCGAGCTCTCCTCACTAAACGGAGA
TGCCAACCATCGGTTGGCTGCTCGTGGACTCCAGGCGCTGACGTATTATCTTTCTACTCCATGCCCAACTTCA

CTATCTTCTGCTTCTGCTGCTACTTTTGCTTCAACAAATCCGAAGTTCTTCAAGAACTCACTGATCAACTTCAA
TGAGATCAGTCCCTGGTTCCGCATACCCAATAGCATTTTTCTCATGGTGTTCAATGGCCAACATTGCTCGAGG
AGTTGA

Amino acid sequence:
MTIDETTSDPISEWLDSPLSYFPSLLDDESYSIDDLLDESYSLDDIYEDSWWAPNESINQEIHINNNNDNSLPCFD
TSFPVTTASTVSLEPVISSHLPPLDSSKKRKGNDSGYNPKASRKNQNRRINDADTRRTTGHKKATHKSSTGNNG
NNREGRWAEQLLNPCAAAITAGNLNRVQHLLYVLRELSSLNGDANHRLAARGLQALTYYLSTPCPTSLSSASAA
TFASTNPKFFKNSLINFNEISPWFRIPNSIFLMVFNGQHCSRS-

SlGRAS51 Solyc12g099220.1.1

Nucleotide sequence (ORF):
ATGGAAGATGCTTCTGGAACTAGTGAATTCTTTCTGGTTAAAGAATCCCTTTCTAGTAGCTCTGGAACAAT
TAGGAGATTCTATACGGGGTTTTGCTTCTGGCGGCAACATGTATGTTGTTGTTTAGCTTCGAAATTGATTC
AAACTAATATGGAAAAATCAATCATCTGGCCGTTTTACGAGCCAGAAGAAAAAACAAATAGTATGCAAGA
ACAAAACGAACAATATTCTGTTAAATTAGATGATTGGGATTTCGACGTTGTAGATAAAACAACAACTATTC
TTTCTATTAAAGAAGCAACAGACGATAACAAAAGAGCGAATAATTCATTGATTTCTTCGCTCAACATATTG
AAAGACTTTGGAAATGGATTTAGAAAACTTAAAGGGCAAAAGATTGATGTTGCTCAAATTAGTGAAAATG
AATGCACATCAAGTGTTTGTAAATTGTCCGCAGAGGAAATTCTTCGATTTGGCGGACAAAAGTTTATGCAA
CGTATTGAATTTGATCATACATTTGTCGACTCATTGTGTAGTCTTTCGGAGGAAGAGTCTAGAAGTGTAAT
GCTCGTCGAACGACTACTTGCTTCTGCAGAGAAAGTCGGGGAGAAGGTTTATGATATCGCGATTGATCTT
TTAAATGAATGCGATAAGTTGAGTTACAAAACAGGGCACCCTGTTGAGAGATTGGTGTACTATTTCTCTCG
TGCTCTACGTGAACGAGTTGATATTGAAACGGGGAAGAATTGCACGAAAGGCTTAGGGATACGTAGAAT
GAGAGATCTTCAGGAGACGTTAAGGAGTATAAACGCGTGCACGATTGCAGTTCAGGACATGCCAATGTG
TGAAGTCGTGAAATTTGCTGGAATACAAGCTATACTTGAAAATGTAGAGAGCTCGAAAAAGATTCATATA
GTTGATCTCGAGATCAAAATGGGAGTGCAATGGACAATCCTAATGCAAGCACTCGCAACTCAATGCAGTA
ACCTTGAATATCTTAAGGTTACTGCATTGATAGATGTTCAATCAAGGACTAACGTCGAGGAGACAGGTAA
ACGACTAATGAGTTTTGCTAAGTCCTTACACTTTCCGTTATGTTTCAAGATAGTTATGGTGGAAGATATCTT
TGATCTAAAGCGAGAAGACTTAGACATCGATCCTGAGGAATCCCTTGCAGTTTACTCGCAATTTTTACTCT
CTAATATGATAACACATCAAGATAGACTCGATTTCTTGATGGGATTCATCAAAGGTTTGAATCCTCGTATA
ATGGTTGTTGCAGAAGTCGAGGCAAATCTTAACTCTCCTGTTTTTGTTAACCGTTTCATTGAAGCTCTATTC
TATCACGGTGCTTACTTTGATTTGTTCGAGGATTGTATGAGAAACAACGAATCAACTAGGAACGCTATGG
AGGAAGAAGTAATGTGGCATGGAATAAGGAACATTATCGCGAATGAGGGTGAGGAAAGGACAATGAGA
CATGTAACGATCGAGTTATGGAGAGAGTATTTCAAGCGATTTGGAATGGTGGAGATGAAAATGAGTGAC
TCGTCGTTGTACCAAGCTAAGACGGTGGTAGAAAAATTTACTGGTAAGAACTCTTTCACGCTCGAGATGA
ATGAAAATTTTGTTACAATTGGATGGAAAGGAACTCCTTTGAACTCACTATCTGCATGGAAGTTCCACAAA
AGAAGCACTTTTCATATAGCTAATTACCTCACTCTTTGA

Amino acid sequence:
MEDASGTSEFFLVKESLSSSSGTIRRFYTGFCFWRQHVCCCLASKLIQTNMEKSIIWPFYEPEEKTNSMQEQNEQ
YSVKLDDWDFDVVDKTTTILSIKEATDDNKRANNSLISSLNILKDFGNGFRKLKGQKIDVAQISENECTSSVCKLS
AEEILRFGGQKFMQRIEFDHTFVDSLCSLSEEESRSVMLVERLLASAEKVGEKVYDIAIDLLNECDKLSYKTGHPV

ERLVYYFSRALRERVDIETGKNCTKGLGIRRMRDLQETLRSINACTIAVQDMPMCEVVKFAGIQAILENVESSKKI
HIVDLEIKMGVQWTILMQALATQCSNLEYLKVTALIDVQSRTNVEETGKRLMSFAKSLHFPLCFKIVMVEDIFDL
KREDLDIDPEESLAVYSQFLLSNMITHQDRLDFLMGFIKGLNPRIMVVAEVEANLNSPVFVNRFIEALFYHGAYF
DLFEDCMRNNESTRNAMEEEVMWHGIRNIIANEGEERTMRHVTIELWREYFKRFGMVEMKMSDSSLYQAKT
VVEKFTGKNSFTLEMNENFVTIGWKGTPLNSLSAWKFHKRSTFHIANYLTL-

SlLS Solyc07g066250.1.1

Nucleotide sequence (ORF):
ATGTTAGGATCCTTTGGTTCTTCATCATCTCAATCTCACCCTCATCATGATGAAGAATCTTCTGATCATCATC
AACAGCGTAGATTCACCGCTACTGCTACAACTATCACCACCACCACCATCACTACCTCACCAGCTATTCAAA
TCCGCCAGCTACTCATTAGCTGTGCGGAGTTGATTTCGCAGTCCGATTTCTCGGCCGCGAAAAGACTCCTT
ACTATATTATCAACTAACTCATCTCCTTTTGGTGATTCAACTGAACGGTTAGTCCATCAATTTACTCGCGCA
CTTTCCCTTCGTCTCAACCGCTATATATCGTCAACCACCAATCATTTCATGACACCTGTTGAAACAACTCCAA
CTGATTCTTCTTCTTCGTCATCATTAGCTCTAATTCAATCATCATATCTATCTCTAAACCAAGTTACCCCTTTC
ATAAGGTTTACTCAATTAACCGCTAATCAAGCGATTTTAGAAGCGATTAACGGTAATCATCAAGCAATCCA
CATCGTTGATTTCGACATTAATCACGGGGTTCAATGGCCACCGTTAATGCAAGCACTAGCTGATCGTTACC
CTGCTCCCACTCTTCGAATCACCGGTACTGGAAATGACCTTGATACCCTTCGTAGAACAGGTGATCGTTTA
GCTAAATTTGCTCACTCATTAGGGTTGAGATTTCAATTCCATCCTCTTTATATAGCCAATAATAACCACGAT
CACGATGAAGATCCTTCTATTATTTCCTCCATTGTACTACTCCCTGATGAAACCCTAGCTATCAACTGTGTTT
TCTACCTCCACCGCCTTTTAAAAGACCGCGAAAAGTTAAGGATTTTTTTGCATAGGGTTAAGTCAATGAAC
CCTAAAATTGTTACAATCGCGGAGAAGGAAGCAAATCATAACCATCCTCTTTTTTTACAAAGATTCATCGA
GGCGTTGGATTATTATACAGCTGTGTTTGATTCACTGGAAGCTACATTGCCACCGGGTAGTCGAGAGAGG
ATGACAGTTGAACAAGTGTGGTTTGGGAGAGAGATTGTTGATATCGTTGCGATGGAAGGAGATAAAAGG
AAAGAAAGACATGAAAGGTTTAGATCATGGGAAGTTATGTTGAGGAGTTGTGGATTTAGTAATGTTGCTT
TAAGCCCTTTTGCATTATCACAAGCTAAGCTTCTTTTGAGACTTCATTATCCTTCTGAAGGCTATCAACTCG
GAGTTTCGAGTAATTCTTTCTTCTTAGGTTGGCAAAATCAACCCCTTTTCTCCATCTCGTCTTGGCGTTGA

Amino acid sequence:
MLGSFGSSSSQSHPHHDEESSDHHQQRRFTATATTITTTTITTSPAIQIRQLLISCAELISQSDFSAAKRLLTILSTNS
SPFGDSTERLVHQFTRALSLRLNRYISSTTNHFMTPVETTPTDSSSSSSLALIQSSYLSLNQVTPFIRFTQLTANQAI
LEAINGNHQAIHIVDFDINHGVQWPPLMQALADRYPAPTLRITGTGNDLDTLRRTGDRLAKFAHSLGLRFQFH
PLYIANNNHDHDEDPSIISSIVLLPDETLAINCVFYLHRLLKDREKLRIFLHRVKSMNPKIVTIAEKEANHNHPLFLQ
RFIEALDYYTAVFDSLEATLPPGSRERMTVEQVWFGREIVDIVAMEGDKRKERHERFRSWEVMLRSCGFSNVAL
SPFALSQAKLLLRLHYPSEGYQLGVSSNSFFLGWQNQPLFSISSWR-

SlDELLA Solyc11g011260.1.1

Nucleotide sequence (ORF):
ATGAAGAGAGATCGAGATCGAGATCGAGAAAGAGAGAAAAGAGCATTCTCTAATGGTGCTGTTTCTTCA
GGGAAAAGTAAGATTTGGGAAGAAGATGAAGAAGAAAAACCAGATGCTGGAATGGATGAGCTTTTAGC
TGTTTTGGGTTATAAAGTGAAGTCGTCTGATATGGCGGATGTTGCTCAAAAACTTGAACAGCTTGAGATG

GCTATGGGTACAACGATGGAAGATGGTATTACTCATCTTTCTACTGATACCGTTCATAAAAACCCATCTGA
TATGGCTGGTTGGGTACAAAGTATGTTATCTTCGATTTCGACAAACTTTGATATGTGTAATCAGGAAAACG
ATGTGCTTGTATCTGGTTGTGGTTCTTCTTCTTCTATAATCGATTTCTCACAAAATCATCGAACAAGTACCAT
TTCTGATGATGATTTAAGAGCTATACCTGGTGGTGCTGTTTTCAATTCGGATAGTAATAAAAGACACAGAT
CAACAACTTCTAGTTTTTCAACTACATCCTCATCTATGGTGACAGATTCATCAGCAACGAGACCTGTTGTAC
TAGTTGATTCACAAGAAACTGGGGTTCGTCTTGTTCATACTTTAATGGCGTGTGCTGAAGCTGTACAACAA
GAAAATTTAACTTTAGCGGATCAACTTGTTAGACATATTGGTATTCTTGCGGTTTCACAATCTGGTGCTATG
AGAAAAGTTGCTACTTACTTTGCTGAAGCATTAGCAAGAAGAATCTACAAAATTTATCCACAAGATTCAAT
GGAATCATCATATACAGATGTTTTACAAATGCATTTCTATGAAACTTGCCCTTATCTCAAATTCGCTCATTTT
ACTGCTAATCAAGCCATTCTTGAAGCGTTTACAGGTTGTAACAAAGTTCATGTAATTGATTTCAGCTTAAAA
CAGGGTATGCAATGGCCTGCACTTATGCAAGCTTTAGCTTTACGCCCCGGTGGACCTCCGGCATTTAGACT
CACCGGAATCGGACCTCCACAGCCGGATAACACAGATGCCTTGCAACAAGTTGGATGGAAGTTAGCTCAG
TTAGCGGAAACTATTGGGGTTGAATTTGAATTCAGGGGATTTGTTGCTAATTCATTAGCAGATCTTGATGC
GACTATACTTGATATAAGGCCAAGTGAAACTGAAGCAGTAGCTATAAACTCTGTTTTTGAGCTTCATCGAT
TGTTATCCCGGCCGGGAGCAATTGAAAAAGTGTTGAACTCTATTAAACAGATTAACCCGAAGATTGTTACT
CTTGTTGAGCAAGAAGCGAATCATAACGCAGGGGTTTTTATTGATAGATTTAACGAAGCTTTGCATTATTA
CTCAACCATGTTTGATTCGTTAGAAAGCTCTGGGTCTTCGTCTTCAGCTTCACCAACTGGGATTCTTCCTCA
ACCTCCGGTGAACAATCAAGATTTGGTGATGTCGGAGGTTTATTTAGGGAGACAGATTTGTAACGTGGTG
GCTTGTGAAGGTTCAGATCGAGTTGAACGACATGAAACACTGAATCAATGGAGGGTTAGGATGAACTCAT
CTGGGTTCGATCCGGTTCATCTGGGTTCAAATGCGTTCAAACAAGCTTCCATGCTTTTAGCTCTGTTCGCCG
GCGGCGATGGTTACAGGGTGGAAGAAAACGATGGGTGTCTTATGTTGGGGTGGCATACACGGCCACTTA
TAGCTACCTCCGCCTGGAAGCTATTGCCGGACTCCGGCACCGGCGCCGGAGAAGTCGAGTTGTAA

Amino acid sequence:
MKRDRDRDREREKRAFSNGAVSSGKSKIWEEDEEEKPDAGMDELLAVLGYKVKSSDMADVAQKLEQLEMAM
GTTMEDGITHLSTDTVHKNPSDMAGWVQSMLSSISTNFDMCNQENDVLVSGCGSSSSIIDFSQNHRTSTISD
DDLRAIPGGAVFNSDSNKRHRSTTSSFSTTSSSMVTDSSATRPVVLVDSQETGVRLVHTLMACAEAVQQENLTL
ADQLVRHIGILAVSQSGAMRKVATYFAEALARRIYKIYPQDSMESSYTDVLQMHFYETCPYLKFAHFTANQAILE
AFTGCNKVHVIDFSLKQGMQWPALMQALALRPGGPPAFRLTGIGPPQPDNTDALQQVGWKLAQLAETIGVE
FEFRGFVANSLADLDATILDIRPSETEAVAINSVFELHRLLSRPGAIEKVLNSIKQINPKIVTLVEQEANHNAGVFID
RFNEALHYYSTMFDSLESSGSSSSASPTGILPQPPVNNQDLVMSEVYLGRQICNVVACEGSDRVERHETLNQW
RVRMNSSGFDPVHLGSNAFKQASMLLALFAGGDGYRVEENDGCLMLGWHTRPLIATSAWKLLPDSGTGAGE
VEL-
