# Supplementary material for: Genome-wide identification, phylogeny and expression analysis of GRAS gene family in tomato
Source: BMC Plant Biol. 2015 Aug 25;15:209. doi: 10.1186/s12870-015-0590-6 (PMC4549011; doi:10.1186/s12870-015-0590-6)
Supplement: Additional file 2: — Tandem duplication events in the 53 SlGRAS genes. (PDF 111 kb) [file 12870_2015_590_MOESM2_ESM.pdf]

Additional file 2: Tandem duplication events in the 53 *SlGRAS* genes.

Cluster number	Gene ID	Chromosome	Start site	End site
1	SlGRAS20	1	69269146	69272222
	SlGRAS21		69280067	69281794
2	SlGRAS22	1	78455070	78458816
	SlGRAS23		78461991	78463478
3	SlGRAS17	2	48257032	48258861
	SlGRAS8		48430009	48431619
4	SlGRAS25	2	53456887	53458410
	SlGRAS26		53622718	53624034
5	SlGRAS30	5	63211136	63213367
	SlGRAS31		63511853	63513271
6	SlGRAS13	6	47399479	47401725
	SlGRAS35		47403325	47404076
7	SlGRAS44	10	65218718	65220250
	SlGRAS45		65222229	65223770
	SlGRAS46		65322155	65324440
