# Supplementary material for: Genome-wide identification, phylogeny and expression analysis of GRAS gene family in tomato
Source: BMC Plant Biol. 2015 Aug 25;15:209. doi: 10.1186/s12870-015-0590-6 (PMC4549011; doi:10.1186/s12870-015-0590-6)

Additional file 3: Multiple sequence alignment showed the other 4 most prominent motifs of GRAS domain: LR I, LR II, PFYRE, SAW, respectively.

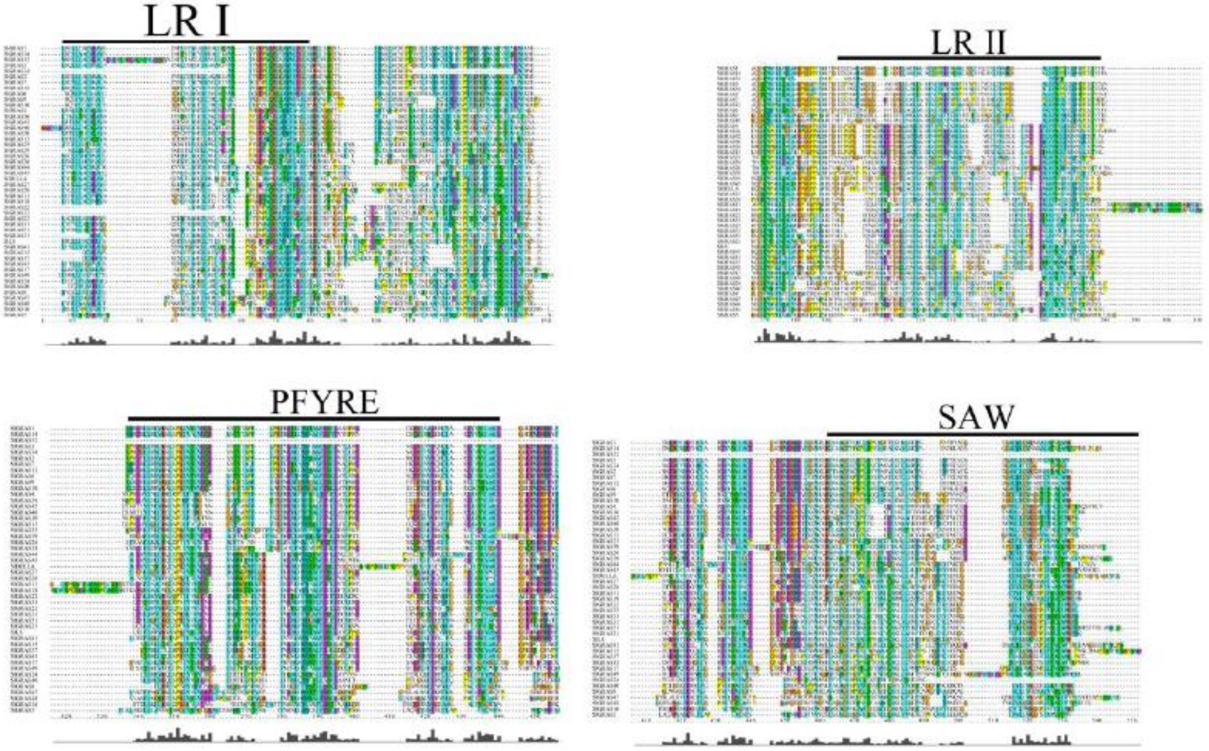

Supplement: Additional file 3: — Multiple sequence alignment showed the other 4 most prominent motifs of GRAS domain: LR I, LR II, PFYRE, SAW, respectively. (PDF 202 kb) [file 12870_2015_590_MOESM3_ESM.pdf]
