# Supplementary material for: Genome-wide identification, phylogeny and expression analysis of GRAS gene family in tomato
Source: BMC Plant Biol. 2015 Aug 25;15:209. doi: 10.1186/s12870-015-0590-6 (PMC4549011; doi:10.1186/s12870-015-0590-6)

Additional file 4: Phylogenetic analysis of GRAS proteins of tomato, potato, and pepper. The phylogenetic tree was generated by Neighbor-Joining method derived from ClustalX alignment of 48, 50, and 30 GRAS amino acid sequences from tomato, potato, and pepper, respectively.

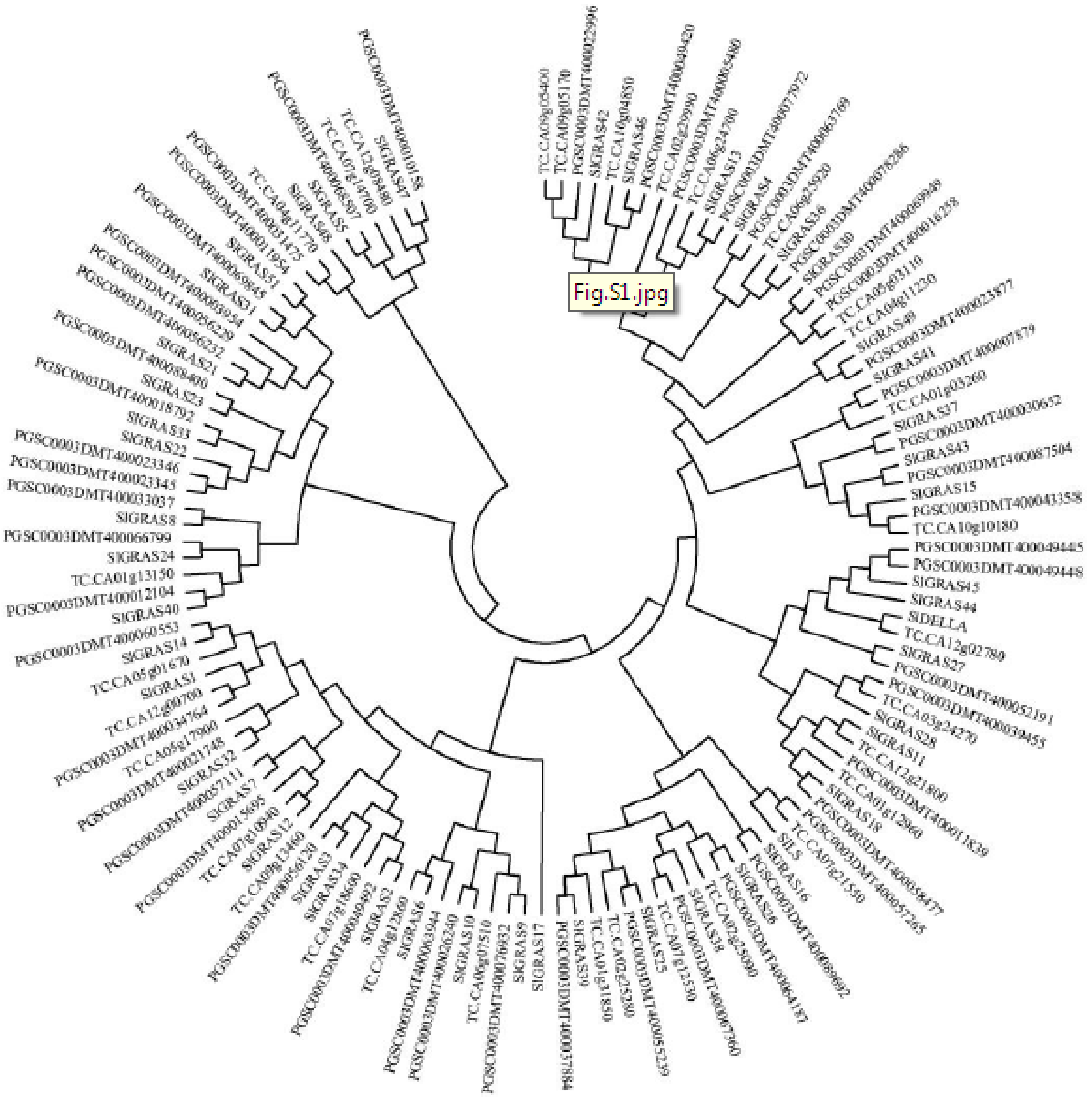

Supplement: Additional file 4: — Phylogenetic analysis of GRAS proteins of tomato, potato, and pepper. The phylogenetic tree was generated by Neighbor-Joining method derived from ClustalX alignment of 48, 50, and 30 GRAS amino acid sequences from tomato, potato, and pepper, respectively. (PDF 291 kb) [file 12870_2015_590_MOESM4_ESM.pdf]
