# Supplementary material for: Genome-wide identification, phylogeny and expression analysis of GRAS gene family in tomato
Source: BMC Plant Biol. 2015 Aug 25;15:209. doi: 10.1186/s12870-015-0590-6 (PMC4549011; doi:10.1186/s12870-015-0590-6)
Supplement: Additional file 5: — Primers used in this work. (PDF 159 kb) [file 12870_2015_590_MOESM5_ESM.pdf]

Additional file 5: Primers used in this work.

Primer name		sequence 5' to 3'	Use
SlGRAS1	F	TCTTCACGCTGGACTCGTCAC	qPCR
	R	CCCACTCAAAGGCGAACCA	
SlGRAS2	F	TCCTTGCAGTAATTCCTCGCG	qPCR
	R	TGGCCCCATTAAAGCGGTCTC	
SlGRAS3	F	GGGCACAAACATCAGGTCAGA	qPCR
	R	CAAGGGCAAGTCCTCCATTACA	
SlGRAS4	F	TTAGGCACCCTTCCATTCCA	qPCR
	R	AAGAATCTCCCACTCCAACAACA	

SlGRAS5	F	TGTCGGTGTATGGAGAGGCAAT	qPCR
	R	GCTGGAATAAGTTTGAAGCAACG	
SlGRAS6	F	TGATGTATTTGCTCGTGGCG	qPCR
	R	ATCTGCCGATGTAGACTCCTTCG	
SlGRAS7	F	TCCCGTGTCTGCTTCTTGTCC	qPCR
	R	GAGTTCCAACGCTCTCATCAGG	
SlGRAS8	F	ACAAACGCTTTCCAACTCGC	qPCR
	R	CGTCGGAGGAACTTGAAGAAC	
SlGRAS9	F	CAGCAGCAACAAGGTTTGGG	qPCR
	R	AATCGGGAGAGGATGGCGT	
SlGRAS10	F	GGATGCTACAGTTCCACGAGACA	qPCR
	R	CCACGATTTGACTAACGGGCT	
SlGRAS11	F	TGAAGGTGGCGTTTGTGGT	qPCR
	R	AAGCAACGCACGCCATTG	
SlGRAS12	F	TAATGGCAGCCTTGGAGGTAATC	qPCR
	R	GGTGAGTGGTAAACAGCGTTGC	
SlGRAS13	F	CCCAAGCCCTGACAGCATTT	qPCR
	R	GGATGACTCTCCACAGGCAAA	
SlGRAS14	F	ATGATGGTAGCAACTCCCAGGC	qPCR
	R	CGTAGATGGCACAGTCAGTAGCC	
SlGRAS15	F	TTCCACGAACTTGTGCGGT	qPCR
	R	TGGCTGATGATGCGATGTTATG	
SlGRAS16	F	TGAACCGCGTGCAACATCTG	qPCR
	R	GCGGAGTCCATGAGCAGCTA	
SlGRAS17	F	TCCCGAGTTGTCCGAATCG	qPCR
	R	AGATGTCTGTGGTTGTAGTGGGC	
SlGRAS18	F	TGGAATGGGATTTACAAGGCG	qPCR
	R	CCACTGTAAAGGCTCGGAAGC	
SlGRAS19	F	GAGGGGGAAAATGTCGCAGTC	qPCR
	R	CGTCCTTCACGGGCGATAATG	
SlGRAS20	F	GCCGAGACAATCAGCACAGG	qPCR
	R	CAAAGCCCTCCATACTTCCAGC	
SlGRAS21	F	GCAGTTGGAACCTCTTTGATTCG	qPCR
	R	TTGCCACAACTTCACCAACCTT	
SlGRAS22	F	TGTGAAGCCAGTGGTGGATG	qPCR
	R	CAATCTCCACCACCAAAGTCCA	
SlGRAS23	F	CATCAAGAAGTTCCATTCACCCA	qPCR
	R	TTCTCTCTGCTGCTGCTTGGAT	
SlGRAS24	F	TGGTCCAGACAGGGAATCCG	qPCR
	R	TTCAAGTTGTTGGTGTTGGCA	
SlGRAS25	F	TGGGCGTTGATGGTTTCG	qPCR
	R	CATTGACTCTGATGGTGGGCA	
SlGRAS26	F	GAACCAACGCCTAACTTGTGGA	qPCR
	R	AACCCGAACCGATGCCAA	

SlGRAS27	F	CATCAAACTGCTTCCAGAGGACT	qPCR
	R	GGATTTCAACATCATCATCGTCG	
SlGRAS28	F	TTCCAGCGTGTAGCGTCCTG	qPCR
	R	GGGTAGCAACATAACCAACCGTG	
SlGRAS29	F	TGTCGGTGTATGGAGAGGCAAT	qPCR
	R	GCTGGAATAAGTTTGAAGCAACG	
SlGRAS30	F	GGAAATGATGGTCGTGATGGTT	qPCR
	R	CGGGACAGTGCCTGACAAA	
SlGRAS31	F	AGTTTGCTGCTGTTCAAGCCA	qPCR
	R	TGTTTGATTTGATGTGAGACCAGAG	
SlGRAS32	F	GCTTGGAGCAGTTGTTCAGCAT	qPCR
	R	TCAAAGTTCGCCGTTGGC	
SlGRAS33	F	CACATCAGATTACACGGAGCCC	qPCR
	R	AAGCAGAAATGGTGACAAGTGGA	
SlGRAS34	F	AGCTATGCTGATATGCTGTG	qPCR
	R	ACTACAGCTTCGTTTCTGAA	
SlGRAS35	F	TTGGGATGCTGACAAGGGA	qPCR
	R	CCTCAACTCTCTCTGCTGGTCTG	
SlGRAS36	F	CAATGAGGAATCAGATAGCGGG	qPCR
	R	TAGGCTCCCAAACTGAGAGTGC	
SlGRAS37	F	TCTCGTTGACGCCGATTCC	qPCR
	R	AGCGAAGTAAGCAGCGACTCG	
SlGRAS38	F	TTGGGAGCCTTGATGGAGC	qPCR
	R	GCCCTAAGGAACCATGATGTGA	
SlGRAS39	F	CCTGTTTCCGATGCCAAGTG	qPCR
	R	CACCATAGGGAGAAGCAAGTTCA	
SlGRAS40	F	TGGTCCACAAGTAATGGCA	qPCR
	R	TCAGCACCTCCAAGTCACAG	
SlGRAS41	F	ATTCGTCTACCGCCATCCTCG	qPCR
	R	TCATCAATACATCCCTGCTCAGC	
SlGRAS42	F	CCGTGAGATTCCAGAAAGACG	qPCR
	R	GCTTGCCACTGCTTGTAGGTC	
SlGRAS43	F	TCGCCACTCGTATGGAAGGG	qPCR
	R	CCCACAACTGGATGGAACTCAAA	
SlGRAS44	F	GCAACAACACTTGCAGTGGA	qPCR
	R	CGAATTAATCTGTCCGCTAG	
SlGRAS45	F	GAGGCGATCCAAGATAACAA	qPCR
	R	CTCAAGACTTGATCCTTGGG	
SlGRAS46	F	GCGGGAAGAAACAGTCTGGTAA	qPCR
	R	TCATCAGCAGCAACAGCCTG	
SlGRAS47	F	GAGTTCACATCTTCAATGACCGC	qPCR
	R	CAAATCAACCATCAAAGGAGGC	
SlGRAS48	F	TTGTTCACCTTCTCATGGCAG	qPCR
	R	TGGGGACGCTAATTCCTTGAG	

SlGRAS49	F	GGCTGCCTATTTTGCTGATGGA	qPCR
	R	ACTCTTCTTCTTGTGTTGGTGGC	
SlGRAS50	F	CACTAAACGGAGATGCCAACCA	qPCR
	R	TGCGGAACCAGGGACTGAT	
SlGRAS51	F	GCACGAAAGGCTTAGGGATACG	qPCR
	R	TCACGACTTCACACATTGGCA	
SlLS	F	CGGTAATCATCAAGCAATCCACA	qPCR
	R	GAAGAGTGGGAGCAGGGTAACG	
SlDELLA	F	GCAAGAAGCGAATCATAACGCA	qPCR
	R	TGAGGAAGAATCCCAGTTGGTG	
SlUBI	F	GCCGACTACAACATCCAGAAGG	qPCR
	R	TGCAACACAGCGAGCTTAACC	
SlGRAS24 GSP1		GAGGCAGTGACCATGACGTTGAATT	5'-RACE for miR171 maping
SlGRAS24 GSP2		CCCATTGCCCGCCATAACCGATA	5'-RACE for miR171 maping
SlGRAS40 GSP1		TTGCCTCACACTCAGATGACCTCAA	5'-RACE for miR171 maping
SlGRAS40 GSP2		GCATAAACGAGGACCATTGAGCACC	5'-RACE for miR171 maping
